# Thermoresponsive Nanogel Incorporating *Duguetia stelechantha* Essential Oil: Biological Activity against *Leishmania* and *Aedes aegypti* Larvae

**DOI:** 10.1021/acsomega.6c04271

**Published:** 2026-08-26

**Authors:** Raiene Lisboa Rocha, Arthur Girardi Francalin Laureano, Thaíssa Gabrielle Corrêa, Melissa Pires Souza, Anderson Ferreira Sepulveda, Agatha Maria Pelosine, Clenilma Marques Brandão, Gustavo Braga, Carlos Alexandre Holanda, Daniele Stéfanie Sara Lopes Lera-Nonose, Daniele Ribeiro de Araujo, Emmanoel Vilaça Costa, Renato Sonchini Gonçalves

**Affiliations:** † Laboratory of Chemistry of Natural Products, Department of Chemistry, 37892 Federal University of Maranhão (UFMA), São Luís 65080-805, Brazil; ‡ Department of Clinical Analysis and Biomedicine, 42487 State University of Maringá (DAB/UEM), Maringá 87020-900, Brazil; § Postgraduate Program in Chemistry, Federal University of Amazonas (UFAM), Manaus 69080-900, Brazil; ∥ Department of Biophysics, 67892 Federal University of São Paulo (UNIFESP), São Paulo, SP 04021-001, Brazil; ⊥ Department of Chemistry, 74362 Federal Institute of Maranhão (IFMA), São Luís 65075-441, Brazil; # University College (COLUN), Federal University of Maranhão (UFMA), São Luís 65080-805, Brazil; ∇ Natural Sciences Degree Coordination, 74353 Federal University of Maranhão, Bom Jesus Campus, Imperatriz, MA 65915-060, Brazil; ° Department of Engineering and Exact Sciences, Palotina Sector, Federal University of Paraná (UFPR), Palotina, PR 85950-000, Brazil

## Abstract

The search for sustainable and effective bioactive compounds from plant sources has intensified due to their potential applications in both healthcare and vector control. In this study, a thermoresponsive nanogel based on Pluronic F127 and Carbopol 974P was developed to incorporate the essential oil of *Duguetia stelechantha* (EODs; essential oil of *D. stelechantha*), aiming to improve its physicochemical stability and evaluate its biological activity in a nanostructured delivery system. The optimized formulation containing 1% (w/w) EODs (nGDs) exhibited suitable physicochemical stability and pronounced thermoresponsive behavior, with a reversible sol–gel transition occurring between 19 and 26 °C. Rheological analysis revealed increased viscosity and enhanced structural organization upon oil incorporation, while FTIR and DLS results confirmed the successful encapsulation of EODs and its influence on intermolecular interactions and nanostructural dynamics. Biological assays demonstrated that free EODs exhibited higher immediate leishmanicidal activity against *Leishmania amazonensis* promastigotes, whereas nGDs showed a time-dependent response, suggesting a possible modulation of essential oil availability within the nanogel matrix. Morphological analysis confirmed significant parasite damage following treatment. Additionally, nGDs exhibited strong larvicidal activity against *Aedes aegypti*, with LC_50_ and LC_90_ values of 97.7 ± 6.4 and 177.7 ± 18.3 μg/mL, respectively. Overall, the developed thermoresponsive nanogel provides a stable platform for incorporating EODs and may contribute to modulating its biological response, although specific release and temperature-dependent biological assays are still required to confirm the functional role of the sol–gel transition. This system represents a promising eco-friendly platform for dual antiparasitic therapy and vector control applications.

## Graphical abstract

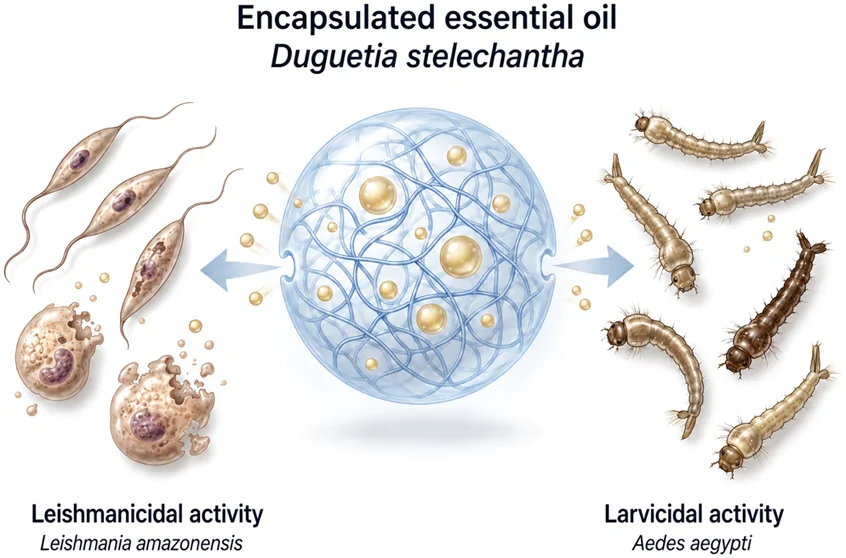

## Introduction

Neglected tropical diseases remain a major global health burden, disproportionately affecting populations in low- and middle-income countries. Among these, leishmaniasis is a severe parasitic disease caused by protozoa of the genus *Leishmania*, with an estimated 700,000 to 1 million new cases annually worldwide.^1,2^ Current therapeutic options, including pentavalent antimonials and amphotericin B, are often associated with high toxicity, elevated costs, and increasing drug resistance, highlighting the urgent need for safer and more effective alternatives.^3^

In parallel, vector-borne diseases transmitted by *Aedes aegypti*, such as dengue, Zika, and chikungunya, have expanded significantly in recent decades, representing a major public health challenge.^4^ The control of mosquito populations remains the most effective strategy to reduce disease transmission. However, the widespread use of conventional insecticides has led to environmental concerns and the development of resistance in mosquito populations, reducing their long-term efficacy.^5–7^ These limitations reinforce the need for alternative and sustainable strategies for vector control.^8,9^

In this context, plant-derived essential oils have emerged as promising candidates due to their wide spectrum of biological activities, including antiparasitic, insecticidal, antimicrobial, and anti-inflammatory properties.^10–12^ Several studies have demonstrated the effectiveness of essential oils against *Leishmania* species, as well as their larvicidal activity against *A. aegypti*, supporting their potential as natural bioactive agents.^13–17^

Despite their promising biological activity, the practical application of essential oils is limited by physicochemical drawbacks, such as high volatility, low water solubility, and chemical instability, which compromise their bioavailability and effectiveness.^18,19^ To overcome these limitations, nanotechnology-based delivery systems have been widely explored, offering improved stability, protection of active compounds, and controlled release profiles.^20–22^

Among these systems, nanogels have attracted particular attention due to their unique structural properties, including high water content, tunable mechanical strength, and ability to respond to environmental stimuli.^23–25^ Thermoresponsive nanogels based on Pluronic F127 are especially relevant because they exhibit reversible sol–gel transitions in response to temperature changes. This behavior has been explored in delivery systems as a physicochemical strategy to improve formulation stability and may contribute to modulated release, depending on the experimental conditions and the incorporated bioactive compound.^26,27^ The incorporation of additional polymers, such as Carbopol, further enhances structural stability and mechanical properties, resulting in robust systems suitable for biomedical applications.^28^

Although the biological potential of essential oils and nanostructured systems has been extensively reported, their combination into thermoresponsive nanogel platforms for dual antiparasitic applications remains underexplored. In particular, essential oils from Amazonian species have shown significant chemical diversity and bioactivity, representing an important source of novel compounds for pharmaceutical and environmental applications.^29^

*Duguetia stelechantha* (Diels) R.E.Fr., a species belonging to the Annonaceae family and native to the Amazon region, has attracted increasing scientific interest due to its rich chemical composition and biological potential.^29,30^ Species of the genus *Duguetia* are known to produce essential oils predominantly composed of monoterpenes and sesquiterpenes, compounds frequently associated with antiparasitic, insecticidal, and antimicrobial activities.^10,12^

A previous study reported the chemical composition and biological activity of *D. stelechantha* essential oil, including its antileishmanial and cytotoxic effects, as well as the evaluation of an essential oil microemulsion.^31^ Although this work demonstrated the biological potential of the free essential oil and its microemulsion, the development of a thermoresponsive nanogel incorporating *D. stelechantha* essential oil, together with its physicochemical characterization and larvicidal evaluation, has not yet been explored.

Previous studies have demonstrated that nanotechnology-based systems can improve the applicability of essential oils by increasing their physical stability, protecting volatile constituents, improving dispersion in aqueous media, and enabling modulation of release or biological responses. Essential oil-loaded nanoemulsions and encapsulated systems have been widely investigated for antimicrobial, pesticidal, and controlled-delivery applications.^32,33^ In addition, micellar nanocarriers are well-established platforms for improving the delivery of poorly water-soluble bioactive compounds.^34^ In this context, thermoresponsive nanogels represent an attractive strategy because they combine polymeric network organization with temperature-dependent physicochemical behavior. However, the application of this approach to *D. stelechantha* essential oil remains unexplored, particularly regarding its incorporation into a Pluronic F127/Carbopol 974P nanogel and its evaluation against both *Leishmania amazonensis* and *A. aegypti*.

In particular, the essential oil of *D. stelechantha* is characterized by a monoterpene-rich profile, with major constituents such as sabinene and terpinen-4-ol, which have been reported to exhibit significant biological effects.^11,35^ This chemical diversity, combined with its natural origin, highlights the potential of this species as a promising source of bioactive compounds for pharmaceutical and vector control applications.^8,9^

Therefore, the present study aimed to develop and characterize a thermoresponsive nanogel incorporating essential oil from *D. stelechantha*, and to evaluate its physicochemical properties and biological activity against *L. amazonensis* promastigotes and *A. aegypti* larvae. Additionally, the influence of essential oil incorporation on the structural, rheological, and diffusion properties of the nanogel was investigated, providing insights into its physicochemical behavior and biological activity as a candidate platform for antiparasitic and vector control applications.

## Materials and Methods

### Materials

Pluronic F127 (poly­(ethylene oxide)–poly­(propylene oxide)–poly­(ethylene oxide) triblock copolymer) was obtained from Sigma-Aldrich (St. Louis, MO, USA). Carbopol 974P NF was kindly provided by IMCD Brasil (São Paulo, Brazil). Anhydrous sodium sulfate (Na_2_SO_4_), XTT [2,3-bis­(2-methoxy-4-nitro-5-sulfophenyl)-5-[(phenylamino)­carbonyl]-2*H*-tetrazolium hydroxide], and *N*-methyl dibenzopyrazine methyl sulfate (PMS) were purchased from Sigma-Aldrich (St. Louis, MO, USA). RPMI 1640 and Medium 199 culture media, as well as fetal bovine serum (FBS), penicillin, and streptomycin, were obtained from Gibco (Thermo Fisher Scientific, NY, USA). Amphotericin B was used as a reference drug in antiparasitic assays. Vectobac (*Bacillus thuringiensis* subsp. *israelensis*) and Temef’os Fersol 1G (temephos) were used as positive controls in larvicidal assays. Ultrapure water was obtained from a Milli-Q purification system (Millipore, Bedford, MA, USA) and used in all experiments.

### Plant Material

Leaves of *D. stelechantha* were collected in September 2023 in the Baixada Maranhense region, municipality of São Bento, Maranhão State, Brazil (02°43′56.8” S; 044°51′27.9” W), in a nonflooded area locally classified as terra firme, characterized by well-drained soil and vegetation not subject to seasonal inundation. The species was taxonomically identified, and a voucher specimen (SLUI 005612) was deposited at the Rosa Mochel Herbarium (SLUI), State University of Maranhão (UEMA), São Luís, Brazil. All procedures were conducted in accordance with Brazilian biodiversity regulations (SisGen registration AA3D4EE).

### Essential Oil Extraction

Leaves were air-dried at room temperature for 48 h prior to extraction. The dried material (300 g) was subjected to hydrodistillation using a Clevenger-type apparatus for 2.5 h after the onset of boiling. The obtained distillate was centrifuged at 3500 rpm for 10 min at 25 °C to facilitate phase separation. The essential oil was collected, dried over anhydrous sodium sulfate (Na_2_SO_4_), and stored in amber vials at 4 °C until further analysis. Moisture content was determined using an IV 2500 infrared moisture analyzer (GEHK), and the extraction yield was calculated based on the dry weight of the plant material (Figure 1).

**Figure 1:**
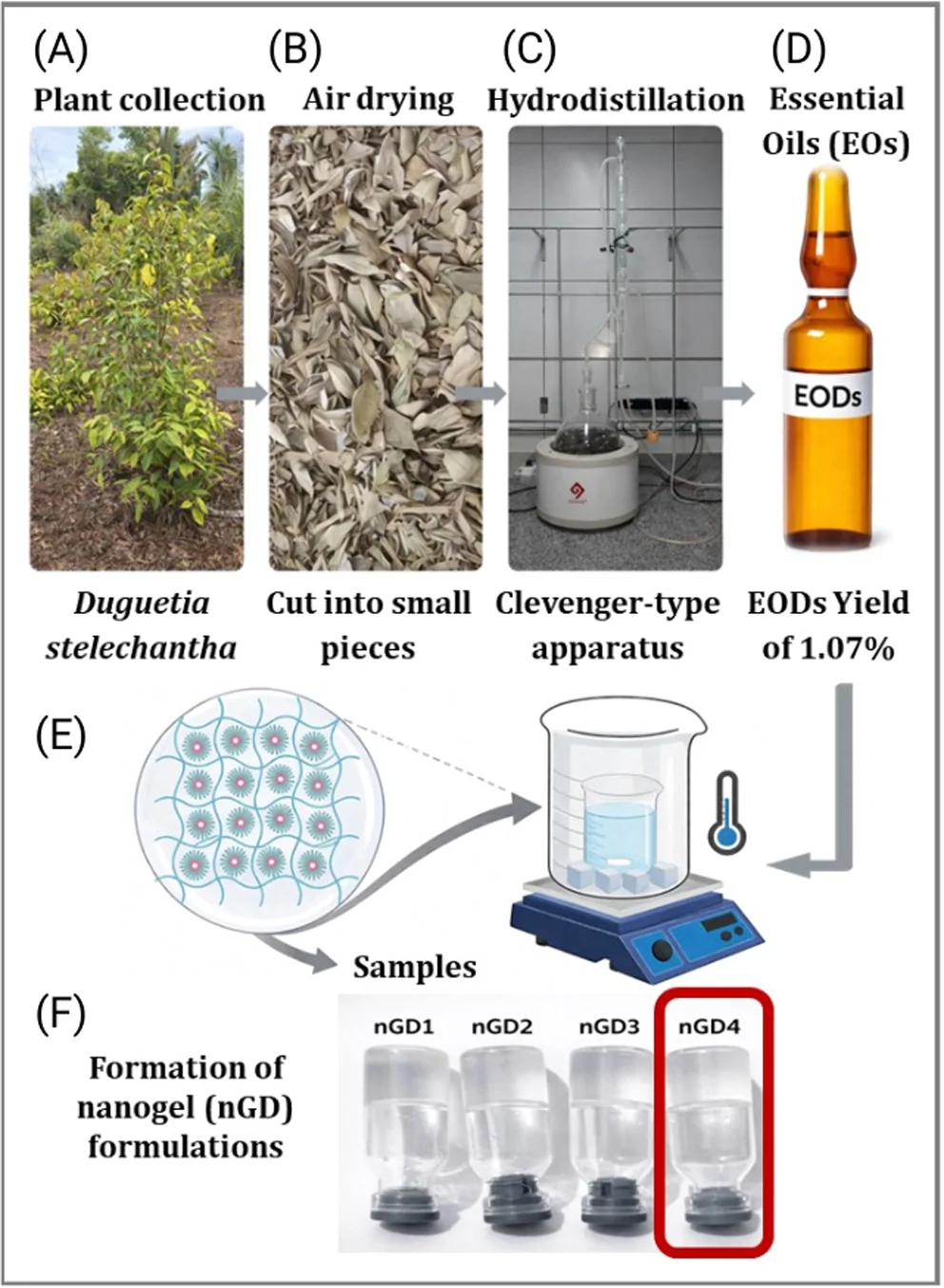
Integrated workflow illustrating the extraction of *D. stelechantha* essential oil (EODs) and its incorporation into nanogel systems. (A–D) Hydrodistillation process and oil recovery; (E) schematic representation of the polymeric nanogel network; (F) macroscopic appearance of the formulations (nG and nGDs). Photographs taken by the authors. Copyright 2026 by the authors.

### Chemical Composition Analysis of the Essential Oil

The chemical profile of EODs was analyzed by GC–MS using a Shimadzu QP2010 Plus system equipped with a ZB-5MS capillary column (30 m × 0.25 mm × 0.25 μm). Helium was used as the carrier gas at a constant flow rate of 1.0 mL min^–1^. The oven temperature was programmed from 40 to 280 °C, with a heating rate of 2 °C min^–1^. The injector and ion source temperatures were maintained at 250 and 280 °C, respectively. Samples of EODs were prepared in CH_2_Cl_2_ at 10 mg mL^–1^ and injected using a split ratio of 1:50.

Relative quantification of the essential oil constituents was carried out by GC–FID using a Shimadzu QP5050A instrument under the same chromatographic conditions described for GC–MS analysis. Compound identification was based on comparison of the obtained mass spectra with the NIST database and on linear retention indices (LRI), calculated using a homologous series of *n*-alkanes according to the Van den Dool–Kratz equation. Compounds showing mass spectral similarity above 90% and LRI values consistent with literature data were considered identified. In the present work, the chemical profile was further confirmed by nuclear magnetic resonance (NMR) spectroscopy to support the structural elucidation of the major constituents. NMR analyses were performed on a Bruker Avance III HD spectrometer (11.75 T), using deuterated chloroform (CDCl_3_) as solvent and tetramethylsilane (TMS) as internal reference. Chemical shifts were expressed in ppm, and coupling constants (*J*) were reported in Hz.

### Nanogel Development and Stability

Nanogel formulations were prepared based on previously reported approaches for the incorporation of essential oils into thermoresponsive nanogel systems.^36^ Pluronic F127 and Carbopol 974P were dissolved in cold distilled water (5–10 °C) at a ratio of 20:0.2% (w/w) under constant stirring Subsequently, the essential oil of *D. stelechantha* (EODs) was incorporated dropwise into the system over 30 min at different concentrations (w/w): 0.125%, 0.25%, 0.5%, 1%, 2.5%, 5%, 10%, and 20%.

The resulting formulations were maintained at 5 °C for 12 h to ensure complete solubilization of all components. The formulations were then subjected to accelerated stability testing at 5 and 25 °C for 14 days, in accordance with guidelines established by the Brazilian Health Regulatory Agency (ANVISA) for cosmetic products and the United States Pharmacopeia (USP).^37,38^ Based on preliminary stability results, the formulation containing 1% EODs was selected for long-term stability evaluation and stored at 5 °C for 180 days. This formulation was designated as nGDs, while the base formulation without EODs was referred to as nG. The composition of the developed formulations is presented in Table 2.

### Dynamic Light Scattering (DLS) Analysis

The hydrodynamic diameter (*D*_h_) and polydispersity index (PDI) of nG and nGDs samples were determined by dynamic light scattering (DLS) using a Litesizer 500 analyzer (Anton Paar GmbH, Graz, Austria), equipped with a BM10 module. Measurements were performed in ultrapure water at 25, 32, 37, and 45 °C (298, 305, 310, and 318 K), using a 40 mW semiconductor laser operating at a wavelength of 658 nm and disposable quartz cuvettes (3.0 mL).

Samples were analyzed under dilute conditions after maximum dilution in ultrapure water to avoid multiple scattering effects and interparticle interactions. The refractive index and viscosity of the dispersant were set to 1.333 and 0.8872 mPa·s (at 25 °C), respectively. All measurements were performed in triplicate, and results were expressed as mean ± standard deviation (SD). The translational diffusion coefficient (*D*) was obtained from the autocorrelation function, and the hydrodynamic diameter (*D*_h_) was calculated using the Stokes–Einstein equation (eq 1), which describes the Brownian motion of spherical particles in a fluid medium^39,40^

1 $$D=\frac{k_{B}T}{6\pi \eta R_{h}}$$

where *k*_B_ is the Boltzmann constant (1.3806503 × 10^–23^ J K^–1^), *T* is the absolute temperature (K), η is the dynamic viscosity of the medium (Pa·s), and *R*_h_ is the hydrodynamic radius (m).

### Rheological Analysis

Rheological measurements were performed using an oscillatory rheometer (Kinexus, Malvern Instruments, UK). A parallel plate geometry was employed for oscillatory tests, while a cone–plate configuration was used for flow measurements. The storage modulus (*G*′), loss modulus (*G*″), and viscosity (η) were evaluated over a temperature range of 0–50 °C at a fixed frequency of 1 Hz and shear stress of 1 Pa. Frequency sweep analyses were conducted from 0.1 to 10 Hz at 32.5 °C.

The viscoelastic properties were described using a power-law model

2 $$G^{\prime }=S\cdot f^{m}$$

where *S* represents the gel strength, *f* is the oscillation frequency, and *m* is the exponent describing the frequency dependence of the elastic modulus.

Flow behavior was further assessed through shear rate-controlled experiments. Samples were subjected to shear rates (γ̇) ranging from 0.1 to 100 s^–1^ at 32.5 °C, and the corresponding shear stress (σ) and apparent viscosity (η) were recorded.

The consistency index (*K*) and flow behavior index (*n*) were obtained using the power-law model

3 $$\sigma =K\cdot {\mathrm{\gamma ̇}}^{n}$$

All measurements were performed in triplicate, and data analysis and model fitting were performed as described in the Statistical and Regression Analyses section.

## In Vitro Larvicidal Assay Against *A. aegypti*

The larvicidal activity of the nGDs nanogel was evaluated against *A. aegypti* larvae following a protocol adapted from the standardized guidelines of the World Health Organization (WHO) for laboratory testing of mosquito larvicides.^41^ Mosquito eggs (*A. aegypti*) were supplied by Biofábrica MOSCAMED Brasil, and hatching and larval rearing procedures were conducted according to a previously described adapted methodology.^42^

Third-instar larvae were used in all bioassays and transferred to polystyrene cups containing 20 mL of test solution. The assay was adapted from WHO guidelines by using 10 larvae per replicate and 10 replicates per concentration, resulting in 100 larvae per tested concentration. Although this design differs from the standard WHO recommendation of 25 larvae per replicate in approximately 100 mL of test solution, the increased number of replicates was used to maintain the total number of larvae per concentration and to allow the evaluation of the nanogel formulation under controlled laboratory conditions. A stock dispersion of nGDs was prepared in mineral water to obtain final concentrations of 5, 50, 150, 250, 350, and 500 μg/mL.

The negative control consisted of the base nanogel (nG) without essential oil droplets (EODs), tested under identical experimental conditions and concentrations. Positive controls included the larvicides temephos (100 μg/mL) and *B. thuringiensis* var. *israelensis* (Bti) (10 μg/mL), each evaluated with ten replicates and 100 larvae per treatment. Mineral water was used as an additional control during larval hatching and development.

Larval mortality was recorded and expressed as percentage values. LC_50_ and LC_90_ values were estimated as described in the Statistical and Regression Analyses section.

### In Vitro Antipromastigote Activity against *Leishmania (L.) amazonensis*

Promastigotes of *Leishmania (Leishmania) amazonensis* (Lla), strain PH8, were cultured in Medium 199 (Gibco, NY, USA) supplemented with 10% heat-inactivated fetal bovine serum (FBS) and antibiotics (100 IU/mL penicillin and 0.1 mg/mL streptomycin). Cultures were maintained at 27 ± 2 °C with periodic subculturing. For experimental assays, parasites were expanded in RPMI 1640 medium (pH 6.8) supplemented with 10% heat-inactivated FBS and incubated for 4 days at 27 ± 2 °C until reaching the late exponential growth phase.

Promastigotes were seeded in 96-well plates containing RPMI 1640 medium (pH 6.8) at a final density of 2 × 10^7^ parasites/mL. The essential oil of *D. stelechantha* (EODs) and the thermoresponsive nanogel containing EODs (nGDs) were evaluated at EODs-equivalent concentrations of 505, 336.5, and 224.4 μg/mL, calculated based on the essential oil content in the formulation. Plates were incubated at 27 ± 2 °C for 24 and 48 h. Promastigote viability was determined using the XTT colorimetric assay [2,3-bis­(2-methoxy-4-nitro-5-sulfophenyl)-5-[(phenylamino)­carbonyl]-2*H*-tetrazolium hydroxide].

The reaction mixture consisted of 20% XTT, 20% PMS (*N*-methyl dibenzopyrazine methyl sulfate), and 60% 0.9% NaCl solution. After incubation for 4 h at 37 ± 2 °C under 5% CO_2_, absorbance was measured at 450 and 620 nm using a microplate reader. The percentage of growth inhibition was calculated relative to untreated controls. Amphotericin B was used as a positive control. All experiments were performed in triplicate and protected from light. Growth inhibition, parasite mortality, and IC_50_ values were calculated as described in the Statistical and Regression Analyses section.

### Statistical and Regression Analyses

Data are expressed as mean ± standard deviation (SD). For rheological measurements, statistical significance was assessed by one-way analysis of variance (ANOVA), followed by Tukey’s post hoc test for multiple comparisons, with differences considered significant at *p* < 0.05. Nonlinear regression was applied for model fitting, and the goodness of fit was evaluated using the coefficient of determination (*R* ^2^).

For the larvicidal assay, larval mortality was recorded as percentage values and analyzed by calculating mean values and standard deviations. The median lethal concentration (LC_50_) and 90 percent lethal concentration (LC_90_) were determined by nonlinear regression analysis of the concentration–response data. Sigmoidal concentration–response curves were fitted, and the adjusted coefficient of determination (*R* ^2^) was calculated using OriginPro software, adopting a 95% confidence interval.

For the antileishmanial assay, the percentage of growth inhibition was calculated relative to untreated controls. Parasite mortality was estimated by logarithmic regression based on a control growth curve generated from untreated promastigotes. IC_50_ values were estimated and reported with 95% confidence intervals. Data were organized using Microsoft Excel 365 and analyzed with GraphPad Prism 8. Statistical significance was considered at a 95% confidence level (*p* < 0.05), when applicable.

## Results and Discussion

### Chemical Composition and NMR Confirmation

The chemical composition of the essential oil of *D. stelechantha* (EODs) was characterized using complementary chromatographic and spectroscopic approaches. Compound identification was primarily performed by GC–MS, based on mass spectral comparison with the NIST library and linear retention indices calculated from a homologous *n*-alkane series. Relative percentages were obtained from GC–FID peak area normalization under the same chromatographic conditions. NMR spectroscopy was used as an additional tool to support the structural assignment of the major constituents.

A total of 38 compounds were identified, accounting for 97.67% of the total chromatographic composition. Monoterpenes were the predominant class (83.11%), followed by sesquiterpenes (14.56%), a profile consistent with that reported for other species of the *Duguetia* genus. Sabinene was identified as the major constituent (41.44%), followed by terpinen-4-ol (17.61%) and spathulenol (9.21%). Other relevant components included *p*-cymene (3.70%), γ-terpinene (3.08%), α-pinene (2.27%), and α-thujene (2.25%). Minor sesquiterpenes, such as caryophyllene oxide (2.90%), (*E*)-caryophyllene (0.82%), and germacrene D (0.45%), were also detected. The GC–MS chromatogram, mass spectra, and NMR spectra are provided in the Supporting Information.

The complete chemical composition of EODs, including experimental and literature retention indices, retention times, relative percentages, and identification criteria, is provided in Table S1. The total ion chromatogram and mass spectra of the identified constituents are shown in Figures S1–S39.

To further support the structural assignment of the major constituents, the chemical profile was confirmed by ^1^H and ^13^C NMR spectroscopy (Table 1). In the ^1^H NMR spectra (Figure S40), sabinene exhibited characteristic signals at δ 4.65–4.83 (H-10), attributed to olefinic protons, along with doublets at δ 0.98 and 0.92 corresponding to methyl groups coupled to vicinal hydrogens. Terpinen-4-ol showed a signal at δ 5.71 (H-2) and a singlet at δ 1.28 (H-7), associated with its methyl group. In contrast, *p*-cymene displayed aromatic proton signals at δ 7.41 and 7.23, as well as a singlet at δ 2.39 (H-7), corresponding to the methyl group attached to the aromatic ring.

**Table 1:** ^1^H and ^13^C NMR Data for the Major Constituents of *D. stelechantha* Essential Oil, Recorded in CDCl_3_ (δ in ppm, *J* in Hz), and Comparison with Literature Values^a^ ^,^ ^b^

compound	position	Exp. δ^1^H (ppm)	Exp. δ^13^C (ppm)	literature data δ^1^H; δ^13^C (ppm)/ref
Sabinene	1	–	37.6	–; 37.4/^46^
	2	1.72–1.78 (2H, m)	27.5	1.75 (m); 27.5/^46^
	3	2.00–2.21 (2H, m)	28.9	2.15 (m); 30.2/^46^
	4	–	154.4	–; –
	5	1.62 (1H, m)	30.1	1.60 (m); 30.2/^46^
	6	0.68–0.69 (2H, d)	16.0	0.69 (d); 16.6/^46^
	7	4.65–4.83 (2H, s)	101.5	4.72 (s); 101.8/ ^46,47^
	8	1.52 (1H, m)	32.6	–; –
	9	0.98 (3H, d)	19.8	0.95 (d); 21.0/^46^
	10	0.92 (3H, d)	20.1	0.93 (d); 21.0/^46^
Terpinen-4-ol	1	–	71.9	–; –
	2	1.20–1.80 (2H, m)	27.1	–; –
	3	5.71 (1H, t)	132.1	5.45 (t); 133.9/^48^
	4	–	69.6	–; 71.8/^48^
	5	1.20–1.80 (2H, m)	34.9	2.11 (t); 34.6/^48^
	6	1.20–1.80 (2H, m)	27.1	2.03 (t); 27.1/^48^
	7	1.28 (3H, s)	29.1	1.24 (s); 29.1/^48^
	8	1.54 (1H, m)	37.3	1.68 (m); 36.9/^48^
	9	0.95 (3H, d)	16.5	0.92 (d); 16.9/^48^
	10	0.89 (3H, d)	17.6	0.95 (d); 17.4/^48^
*p*-Cymene	1	–	137.2	–; 134.8/ ^49,50^
	2	7.41 (1H, d)	129.2	7.15 (d); 126.3/^49^
	3	7.23 (1H, d)	125.4	7.11 (d); 126.3/^49^
	4	–	141.6	–; 145.1/ ^49,50^
	5	7.23 (1H, d)	125.4	7.11 (d); 126.3/^49^
	6	7.41 (1H, d)	129.2	7.15 (d); 126.3/^49^
	7	2.39 (3H, s)	21.0	2.32 (s); 20.9/ ^49,50^
	8	1.63 (1H, m)	26.1	–; 33.7/ ^49,50^
	9	0.95 (3H, d)	22.2	–; 23.0/^49^
	10	0.84 (3H, d)	21.6	–; 23.9/^49^

a Compounds include sabinene, terpinen-4-ol, and *p*-cymene. Signal assignments follow the corrected atom numbering shown in Figure 2. Literature values are included when unambiguously assigned in the cited references.

b CDCl_3_: deuterated chloroform; δ: chemical shift; *J*: coupling constant; m: multiplet; d: doublet; t: triplet; s: singlet. Dashes indicate unavailable or nonunambiguously assigned data.

The ^13^C NMR spectra (Figures S41 and S42) revealed, for sabinene, an olefinic carbon signal at δ 154.38 (C-4) and aliphatic carbons in the range of δ 19.79–28.96. Terpinen-4-ol presented signals at δ 132.1 (C-2, olefinic) and δ 71.9 (C-4, hydroxyl-bearing carbon). For *p*-cymene, aromatic carbons were observed at δ 129.2 and 125.4, while the methyl group attached to the ring appeared at δ 21.0. These assignments are consistent with previously reported data.^43–45^

The chemical profile observed in the present study differs from that previously reported for *D. stelechantha* essential oil, in which β-pinene, α-pinene, and spathulenol were described as the major constituents.^31^ In contrast, the oil analyzed here was characterized by sabinene, terpinen-4-ol, and spathulenol as the main components. These differences may be associated with variations in plant collection site, environmental conditions, phenological stage, and extraction or analytical parameters. Importantly, while the previous study focused on the free essential oil and a microemulsion system, the present work advances this topic by incorporating EODs into a thermoresponsive nanogel and evaluating its physicochemical behavior, larvicidal activity, and time-dependent antileishmanial response (Figure 2).

**Figure 2:**
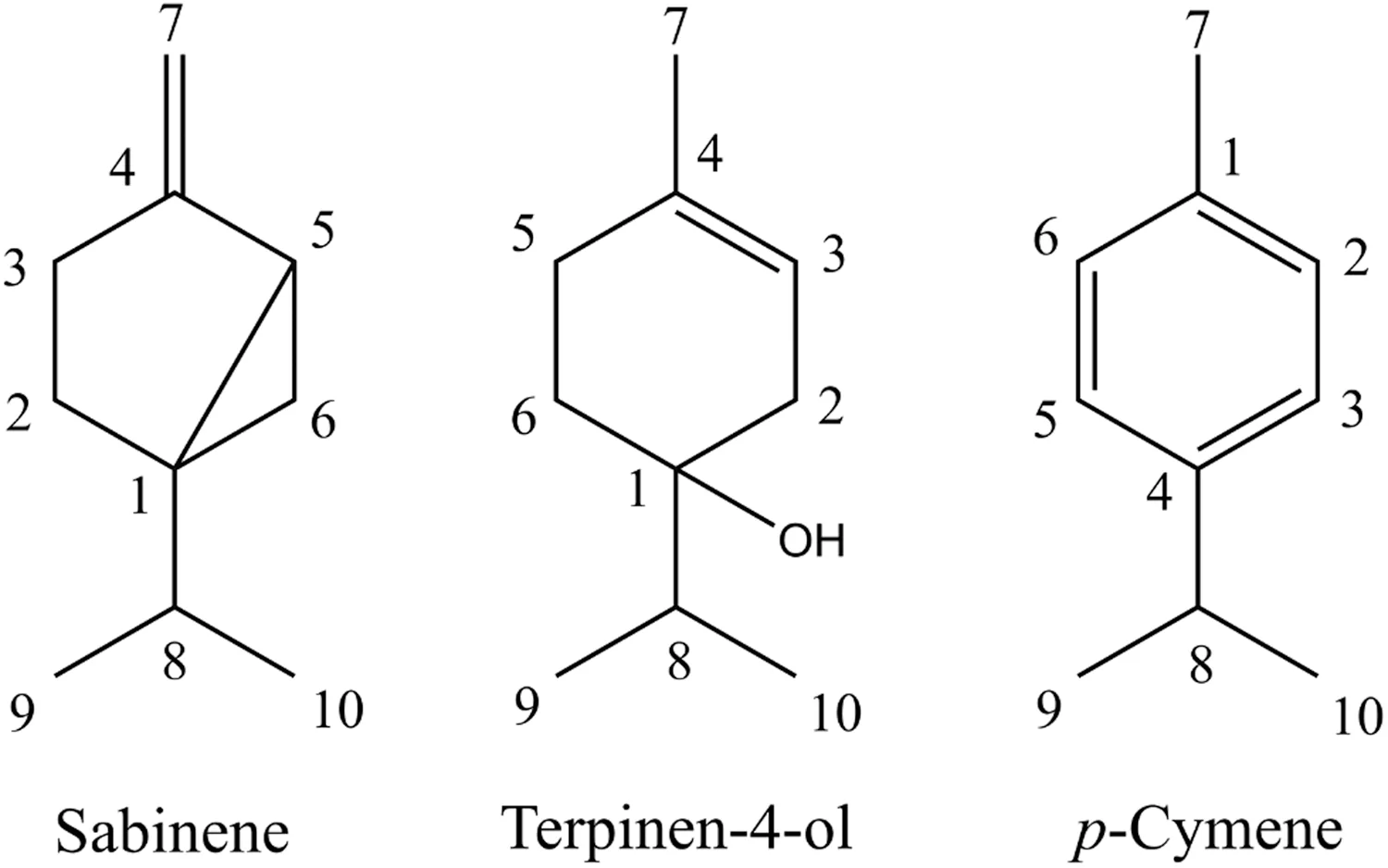
Chemical structures and atom numbering of the major constituents of *D. stelechantha* essential oil (EODs). From left to right: sabinene (41.44%), terpinen-4-ol (17.61%), and *p*-cymene (3.70%), identified as the predominant components by GC–MS analysis. These compounds belong to the monoterpene fraction, which represents the major chemical class in the oil.

The formulation development and stability results are described in the following subsection.

### Nanogel Formulation and Stability Evaluation

The base formulation (nG) was developed based on previous studies that established an optimal polymer ratio of 20:0.2% (w/w) between Pluronic F127 and Carbopol 974P to achieve the desired temperature-responsive behavior.^36^ Based on this matrix, a series of formulations (F1–F8) was prepared by incorporating increasing concentrations of EODs (Table 2). Formulations F1–F4, containing 0.125–1% (w/w) EODs, exhibited adequate homogeneity and physical stability during preparation and after 24 h of storage. In contrast, formulations F5–F7, containing higher oil loadings (2.5%, 5%, and 10%), showed clear signs of instability, including initial turbidity followed by progressive phase separation and formation of an upper oily layer.

**Table 2:** Composition (% w/w) and Physical Behavior of Nanogel Formulations (F1–F8) Loaded with *Duguetia* Essential Oil Derivatives (EODs)^a^

formulation	water	F127	carbopol 974P	EODs	physical behavior
F1	79.68	20.0	0.20	0.125	Stable nanogel^b^
F2	79.55	20.0	0.20	0.25	Stable nanogel^b^
F3	79.30	20.0	0.20	0.50	Stable nanogel^b^
F4	78.80	20.0	0.20	1.00	Stable nanogel^b^
F5	77.30	20.0	0.20	2.50	Phase separation
F6	74.80	20.0	0.20	5.00	Phase separation
F7	69.80	20.0	0.20	10.00	Phase separation
F8	59.80	20.0	0.20	20.00	Stable nanoemulsion-like^c^

a F127 corresponds to Poloxamer 407, and Carbopol 974P was used as the gelling agent. The formulations were evaluated in terms of physical stability, distinguishing stable nanogel systems, phase-separated systems, and nanoemulsion-like systems as a function of EODs concentration.

b Transparent, homogeneous nanogel system.

c Opaque nanoemulsion-like system.

Although formulation F8, containing 20% EODs, initially appeared stable, its high lipophilic content impaired the homogeneous dispersion of EODs within the aqueous phase. This behavior suggested the formation of a system more consistent with an oil-in-water (O/W) nanoemulsion-like formulation rather than a true nanogel, highlighting the limitations associated with excessive oil loading. Based on these preliminary observations, formulations F1–F4 were selected for accelerated stability studies, following ANVISA guidelines.^51^ The formulations were subjected to alternating temperature cycles (5 and 25 °C) over a 14-day period.

No visual changes, phase separation, or alterations in organoleptic properties were observed throughout the testing period, as confirmed by the images provided in the Supporting Information. These findings demonstrate the physicochemical robustness of the selected formulations under thermal stress and light exposure. Among the evaluated systems, formulation F4, which contained 1% EODs and represented the highest essential oil loading while maintaining nanogel stability, was selected for subsequent experiments and is hereafter referred to as nGDs.

### FTIR Analysis

Fourier-transform infrared (FTIR) spectroscopy was employed to investigate the molecular interactions and structural organization of the nanogel systems, as well as to evaluate the incorporation of EODs. The FTIR spectra of the blank nanogel formulation (nG) and the EODs-loaded nanogel formulation (nGDs) are provided in the Supporting Information as Figure S43, allowing verification of the spectral changes discussed below. The main FTIR bands and their corresponding assignments are summarized in Table 3.

**Table 3:** Main FTIR Bands and Assignments for EODs, Polymers, and Nanogel Formulations^a^

sample	wavenumber (cm^–1^)	assignment	interpretation
Essential Oil			
EODs	2954–2831 ^52^	C–H stretching	Aliphatic chains (terpenes)
	1639 ^52^	C=C stretching	Unsaturated compounds
	1458, 1369 ^53^	C–H bending	Methyl/methylene groups
	875 ^52^	C=C deformation	Out-of-plane bending
Polymers			
F127	3564 ^52,53^	O–H stretching	Hydrogen bonding (PEO)
974P	3475–3414 ^52,54^	O–H stretching	Carboxylic groups/dimers
	1720 ^54^	C=O stretching	Intermolecular H-bonding
Nanogels			
nG	3384 ^53,54^	O–H stretching	F127–974P interactions
	3564 ^53^	O–H stretching	Residual F127 micelles
nGDs	3465 ^52^	O–H stretching	Weakened H-bond network
	∼2900 ^53^	C–H stretching	Hydrophobic interactions (EODs–PPO)
	∼1730 ^54^	C=O stretching	Modified polymer interactions

a FTIR spectra of the essential oil (EODs) were recorded using attenuated total reflectance (ATR), whereas polymer and nanogel samples were analyzed as lyophilized powders using the KBr pellet method.

The FTIR spectrum of EODs exhibited characteristic bands in the region of 2954–2831 cm^–1^, corresponding to symmetric and asymmetric C–H stretching vibrations of aliphatic chains.^52^ A weak band observed at 1639 cm^–1^ was attributed to C=C stretching, while bands at 1458 and 1369 cm^–1^ were assigned to bending vibrations of C–H bonds. Additionally, an intense band at 875 cm^–1^ was associated with out-of-plane deformation of C=C bonds, confirming the presence of unsaturated terpenoid constituents.

For Pluronic F127, the absorption band around 3564 cm^–1^ was associated with O–H stretching vibrations, reflecting hydrogen bonding involving the poly­(ethylene oxide) segments.^52,53^ The spectrum of Carbopol 974P displayed O–H stretching bands between 3475 and 3414 cm^–1^, characteristic of carboxylic groups, including hydrogen-bonded dimers. A prominent band at 1720 cm^–1^ was assigned to C=O stretching, indicative of intermolecular hydrogen bonding.^52,54^

The FTIR spectrum of the nanogel (nG) revealed a broad absorption band centered at 3384 cm^–1^, resulting from overlapping contributions of F127 and 974P. This shift, relative to the individual polymer spectra, indicates the formation of intermolecular hydrogen bonds between F127 and 974P. The coexistence of a band near 3564 cm^–1^ suggests that micellar domains of F127 remain partially preserved within the nanogel matrix.

Upon incorporation of EODs (nGDs), a shift of the O–H stretching band to 3465 cm^–1^ was observed, suggesting modifications in the hydrogen-bonding environment of the polymeric network after oil incorporation. Additionally, an increase in intensity and a slight shift in the C–H stretching region around 2900 cm^–1^ were observed, consistent with the contribution of hydrophobic terpene constituents from EODs and possible interactions with the PPO domains of Pluronic F127. A subtle shift in the carbonyl band of 974P, from 1724 to approximately 1730 cm^–1^, further supports modifications in the local interaction environment of the polymeric matrix.

Overall, these spectral changes support the incorporation of EODs into the nanogel matrix and suggest interactions among Pluronic F127, Carbopol 974P, and the essential oil components. These FTIR findings are consistent with the DLS results, which showed that EODs incorporation modified the temperature-dependent nanostructural behavior of the system, and with the rheological data, which indicated increased viscosity, gel strength, and structural organization for nGDs. Therefore, the combined FTIR, DLS, and rheological analyses support that EODs modulates intermolecular interactions and nanogel organization. However, these data should be interpreted as physicochemical evidence of formulation interactions and not as direct measurements of encapsulation efficiency, release kinetics, or biological performance.

### Dynamic Light Scattering (DLS) Analysis

Dynamic light scattering (DLS) was employed to determine the hydrodynamic diameter (*D*_h_) and polydispersity index (PDI) of the nanogels, based on fluctuations in scattered light intensity resulting from the Brownian motion of particles. This technique provides valuable insights into colloidal stability and thermoresponsive behavior under varying environmental conditions, such as temperature changes.^55^

The formulations nGF and nGDs exhibited distinct temperature-dependent behaviors, reflecting differences in their structural organization and thermal responsiveness (Table 4). When the concentration of Pluronic F127 exceeds the critical micellar concentration (CMC), spontaneous micelle formation occurs, with hydrophobic poly­(propylene oxide) (PPO) cores encapsulating lipophilic compounds and hydrophilic poly­(ethylene oxide) (PEO) chains stabilizing the system.^45^

**Table 4:** Dynamic Light Scattering (DLS) Measurements as a Function of Temperature for Nanogels nG and nGDs^a^ ^,^ ^b^

	nG		nGDs	
temperature (°C)	*D*_h_ (nm)	PDI	*D*_h_ (nm)	PDI
25	661 ± 6	0.34	270 ± 73	0.29
32	124 ± 5	0.24	293 ± 16	0.44
37	17 ± 3	0.25	268 ± 31	0.42
45	17 ± 9	0.26	208 ± 10	0.23

a Data are expressed as mean ± SD (*n* = 5).

b DLS measurements were performed after maximum dilution of nanogel samples in ultrapure water to avoid multiple scattering effects and interparticle interactions.

This process is further enhanced above the critical micellization temperature (CMT), promoting copolymer self-assembly.^56^ For the base formulation (nGF), increasing the temperature from 25 to 32 °C induced the formation of smaller and more homogeneous micelles, resulting in a marked reduction in D_*h*_ (from approximately 661 to 124 nm) and a decrease in PDI (from 0.34 to 0.25).

At higher temperatures (37 and 45 °C), a further reduction in particle size was observed, with hydrodynamic diameters approaching 17 nm and stable PDI values around 0.25. This behavior indicates enhanced diffusion and structural rearrangement into compact micellar assemblies, driven by stronger hydrophobic interactions within the micellar core. Such thermoresponsive contraction demonstrates the temperature-dependent physicochemical behavior of the system. However, its direct impact on biological performance was not evaluated in the present study and should be investigated in future release and temperature-dependent biological assays.

In contrast, the nGDs formulation exhibited a more controlled response to temperature variation. Hydrodynamic diameters ranged from approximately 208 to 293 nm across the evaluated temperature range, indicating increased structural stability. The presence of EODs likely interferes with micellar packing, reducing the extent of thermal contraction.

Between 25 and 32 °C, a moderate increase in *D*_h_ (270 to 293 nm) and PDI (0.29 to 0.44) was observed, suggesting thermally induced swelling of the micellar structure. From 32 to 37 °C, partial structural reorganization occurred, with a slight decrease in *D*_h_ and stabilization of PDI values. At higher temperatures (37 to 45 °C), a more pronounced contraction was observed (*D*_h_ approximately 208 nm; PDI approximately 0.23), accompanied by increased diffusion coefficients (Figure 3).

**Figure 3:**
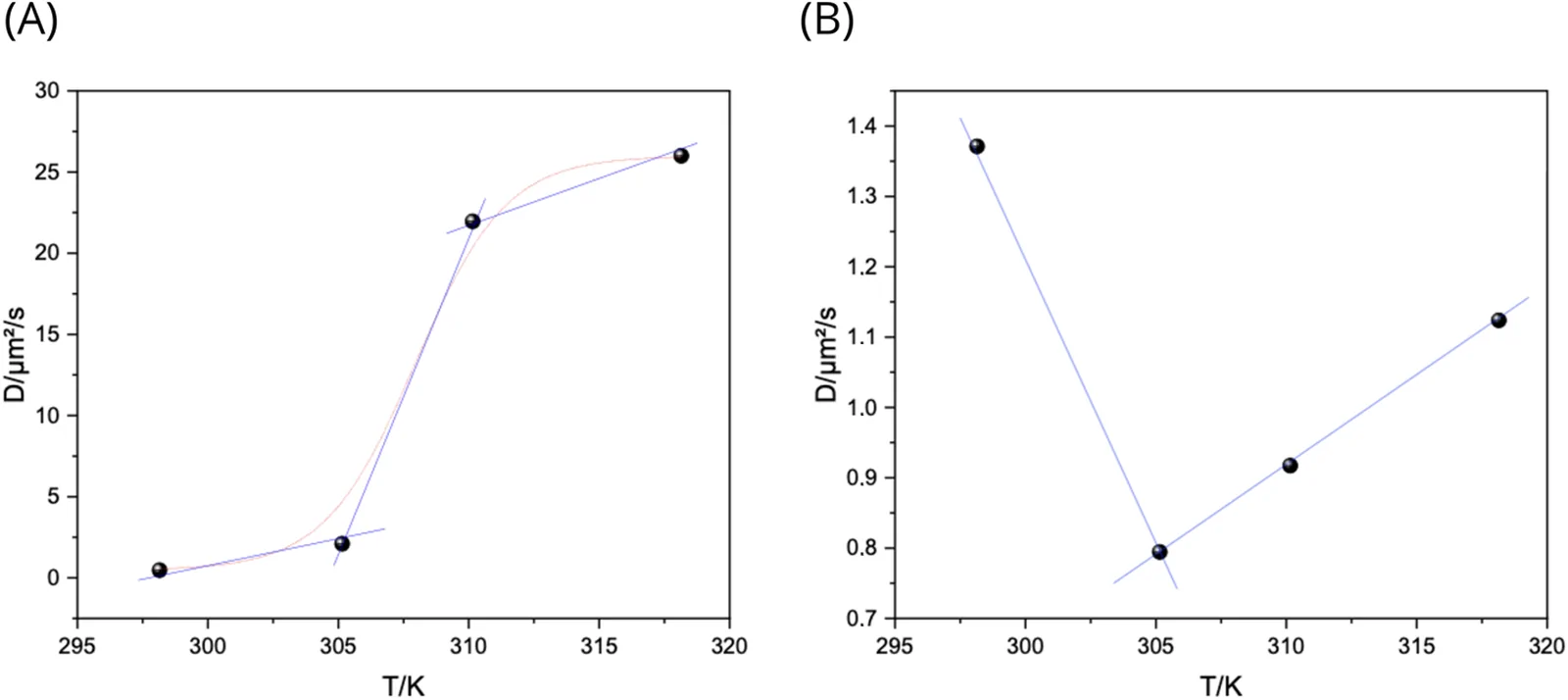
Variation of the diffusion coefficient (Dif) as a function of temperature (*T*, K) for nanogels (A) nG and (B) nGDs, obtained from DLS measurements.

This behavior is characteristic of thermoresponsive nanogels and indicates that the incorporation of EODs modulates micellar dynamics, promoting structural rearrangements that enhance diffusion while maintaining colloidal stability.

Overall, the incorporation of essential oil modified the physicochemical behavior of the nanogel by balancing structural stability and thermoresponsive properties. These characteristics may be relevant for future controlled-release applications, but specific release assays are required to confirm this functionality.

### Rheological Behavior

Rheological analysis was performed to investigate the viscoelastic properties, structural organization, and flow behavior of the nanogel systems (Figure 4). Both nG and nGDs exhibited a typical thermoresponsive sol–gel transition, characterized by the crossover between the viscous modulus (*G*″) and the elastic modulus (*G*′), occurring within the temperature range of approximately 19–26 °C.

**Figure 4:**
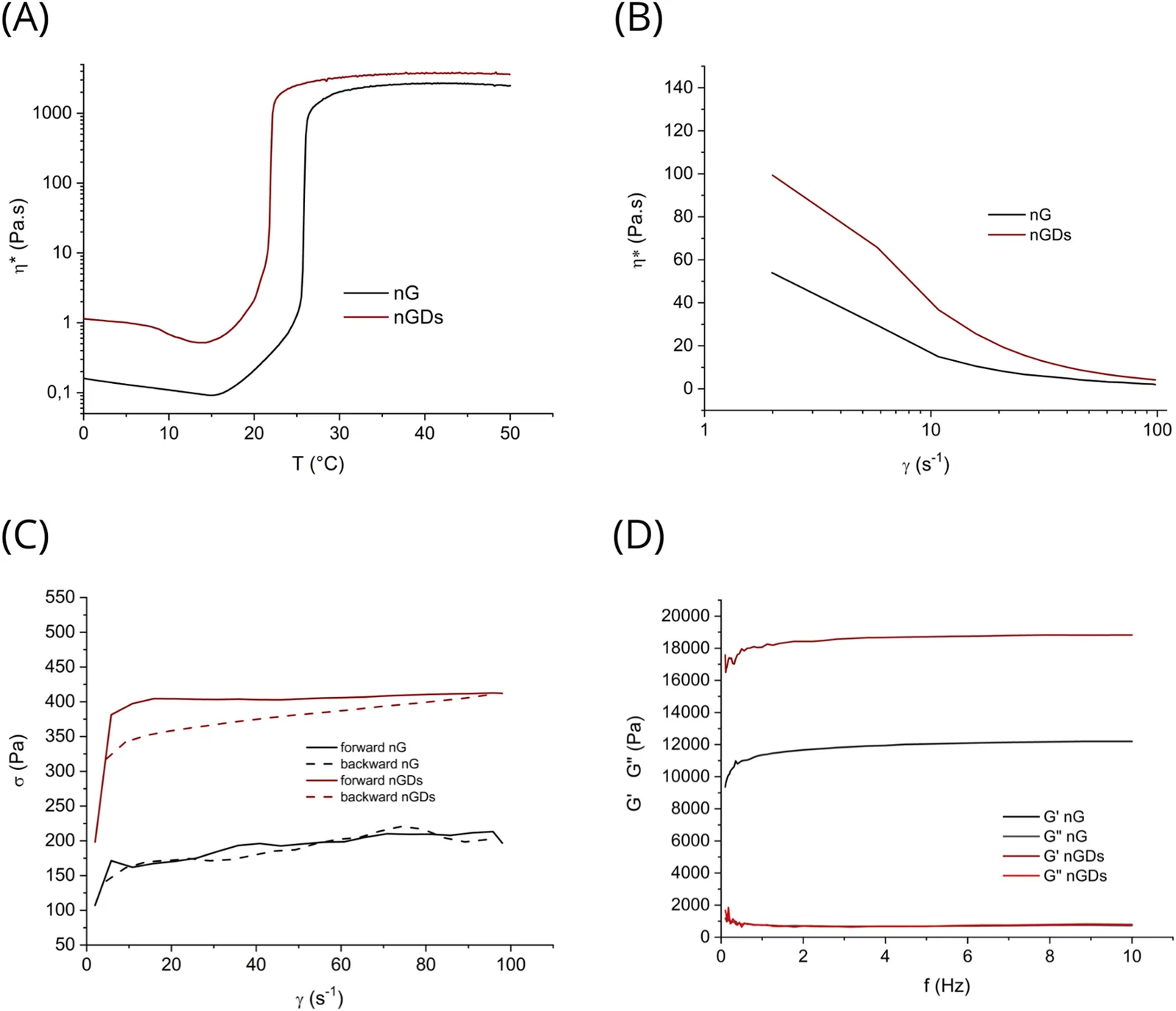
Rheological characterization of nG and nGDs systems. (A) Temperature-dependent viscoelastic moduli (*G*′ and *G*″) as a function of temperature. (B) Frequency sweep analysis showing viscoelastic behavior over a range of frequencies. (C) Apparent viscosity as a function of shear rate. (D) Flow behavior curves illustrating the rheological response of the systems.

Below the transition temperature, the systems behaved predominantly as viscous fluids (*G*″ > *G*′), with low structural organization and reduced viscosity. As temperature increased, a transition to an elastic-dominated regime (*G*′ > *G*″) was observed, indicating the formation of a three-dimensional polymeric network driven by temperature-induced micellization of Pluronic F127. This transition was accompanied by a significant increase in viscosity, reaching values between 2.0 and 4.0 kPa·s.

The nGDs formulation exhibited a lower sol–gel transition temperature (approximately 22 °C) and higher viscosity compared to nG, suggesting that the incorporation of EODs enhances intermolecular interactions within the polymeric network. This effect is likely associated with hydrophobic interactions between essential oil components and the poly­(propylene oxide) (PPO) domains, leading to improved network stabilization and gel structuring.

At temperatures above 40 °C, both systems showed a slight decrease in viscosity, indicating partial structural rearrangement or weakening of the network under thermal stress.

Frequency sweep analysis demonstrated that both formulations exhibited weak frequency dependence, with *G*′ remaining higher than *G*″ across the evaluated frequency range, indicating the ability of the systems to maintain structural integrity under oscillatory deformation. The power-law analysis revealed values of *m* < 1 for all systems, confirming the presence of structured viscoelastic networks. Notably, nGDs exhibited higher gel strength (*S*) and lower *m* values, indicating a more cohesive and organized internal structure.

Flow curve analysis further showed that both formulations behave as non-Newtonian, pseudoplastic fluids, characterized by shear-thinning behavior. The nGDs system presented higher consistency index (*K*) and lower flow behavior index (*n*), indicating greater resistance to flow at low shear rates and enhanced structural organization. A slightly higher hysteresis observed for nGDs suggests partial structural disruption under shear, likely associated with rearrangement of oil-loaded domains.

Overall, the rheological results demonstrate that the incorporation of EODs significantly modifies the viscoelastic and flow properties of the nanogel, promoting enhanced structural organization and mechanical strength. These findings are consistent with FTIR and DLS analyses, confirming that EODs modulates intermolecular interactions and nanostructural dynamics within the system (Table 5).

**Table 5:** Rheological Parameters of Nanogels nG and nGDs Obtained from Temperature, Frequency, and Flow Analyses^a^

parameter	nG	nGDs
	temperature-dependent analysis	
*T*_sol–gel_ (°C)	26.2 ± 0.2	22.1 ± 0.1
*G*′/*G*″	0.065 ± 0.001	0.026 ± 0.001
η (Pa·s)	2151.5 ± 140.1	3313.5 ± 90.7
	frequency sweep (power-law model)	
*S* (Pa·s^*n*^)	10533.0 ± 23.8	18000.0 ± 33.8
*m*	0.052 ± 0.002	0.026 ± 0.001
	flow behavior (power-law model)	
*K* (Pa·s^*n*^)	115.7 ± 6.4	233.0 ± 1.7
*n*	0.13 ± 0.01	0.08 ± 0.01

a Data are expressed as mean ± SD.

### Larvicidal Activity of nGDs against *A. aegypti*

The larvicidal efficacy of the nanogel formulation (nGDs) was evaluated against third-instar *A. aegypti* larvae after 24 h of exposure, and the results are summarized in Table 6. A clear dose-dependent response was observed, with progressively higher mortality rates as the concentration increased. At lower concentrations, larval mortality remained limited, whereas higher concentrations induced pronounced lethality, indicating strong biological activity.

**Table 6:** Larvicidal Efficacy of nGDs against *A. aegypti* Larvae after 24 h of Exposure^f^

	nGDs^a^	
treatment (μg/mL)	larvae (*n*)	mortality (% ± SD)
5	100	0.0 ± 0.0
50	100	7.0 ± 10.6
150	100	80.0 ± 20.0
250	100	89.0 ± 9.9
350	100	92.0 ± 11.3
500	100	95.0 ± 8.5
Mineral water (blank)^b^	100	0.0 ± 0.0
nG (negative control)^c^	100	0.0 ± 0.0
Temephos (positive control 1)^d^	100	100.0 ± 0.0
Bti (positive control 2)^e^	100	100.0 ± 0.0

a Concentration expressed as EODs-equivalent concentration in the nanogel formulation (nGDs).

b Blank control used during larval hatching and development.

c Nanogel base without essential oil.

d Chemical larvicide positive control.

e Biological larvicide positive control.

f Concentrations are expressed as EODs-equivalent concentration (μg/mL). Results are expressed as mortality (% ± standard deviation, SD). Experiments were performed in 10 replicates (n = 100 larvae per treatment).

Importantly, no mortality was observed in the negative control (nG), confirming that the larvicidal effect is directly associated with the encapsulated essential oil rather than the polymeric matrix. This finding highlights the role of the nanogel as a functional carrier system rather than an active agent itself. In contrast, conventional chemical larvicides such as temephos have been associated with increasing resistance in mosquito populations and environmental toxicity toward nontarget organisms.^57,58^ In this context, the development of alternative systems based on natural bioactive compounds represents a relevant and sustainable strategy for vector control.

The concentration–response profile (Figure 5A) exhibited a typical sigmoidal behavior, indicating a well-defined dose–response relationship. The calculated lethal concentrations further support the high efficacy of the system, with LC_50_ and LC_90_ values of 97.7 ± 6.4 μg/mL and 177.7 ± 18.3 μg/mL, respectively, and an adjusted coefficient of determination (*R* ^2^ = 0.9992), demonstrating excellent model fitting.

**Figure 5:**
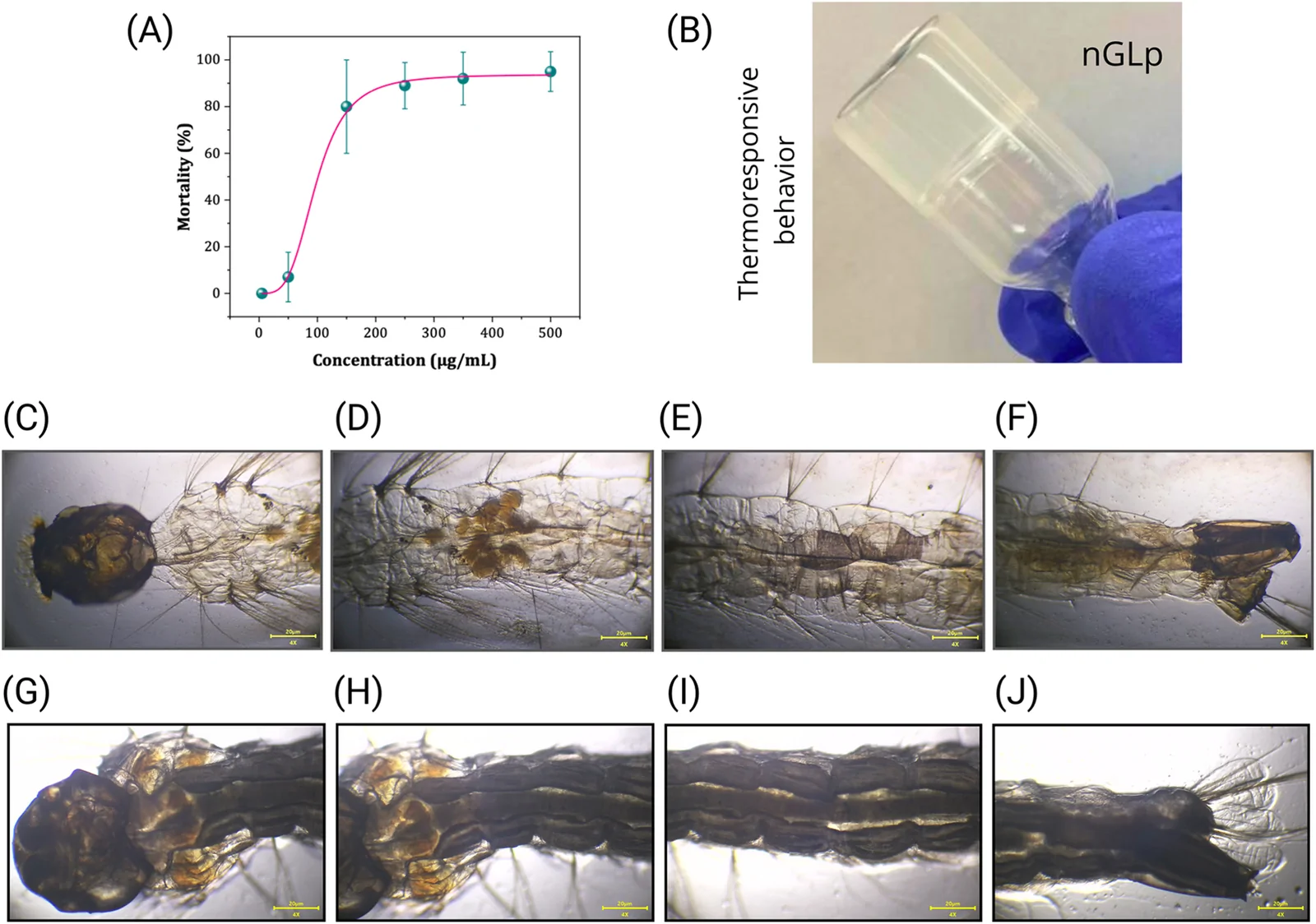
Larvicidal activity and morphological effects of the thermoresponsive nanogel (nGDs) against *A. aegypti* larvae. (A) Dose–response curve showing larval mortality (%) as a function of EODs-equivalent concentration after 24 h of exposure. (B) Visual demonstration of the temperature-responsive behavior of the nanogel, illustrating the sol–gel transition upon temperature increase. (C–F) Optical microscopy images of third-instar *A. aegypti* larvae exposed to nGDs, showing structural disorganization, deformation of anatomical regions, and damage to internal tissues. (G–J) Control larvae (mineral water) exhibiting preserved anatomical structures, including head (G), thoracic region (H), abdominal segments (I), and anal segment with siphon (J). Scale bar = 20 μm. Photograph and optical micrographs acquired by the authors. Copyright 2026 by the authors.

The larvicidal activity can be attributed to the bioactive compounds present in the essential oil, particularly monoterpenes, incorporated into the nanogel matrix. The nanogel matrix may favor the dispersion of the essential oil in the aqueous medium, which could contribute to the observed larvicidal response. However, since free EODs were not included as a direct comparator in this assay, no conclusion can be drawn regarding improved larvicidal activity or enhanced bioavailability. Moreover, the thermoresponsive behavior of the system (Figure 5B) demonstrates an important physicochemical property of the formulation. Nevertheless, its direct contribution to larval exposure, essential oil release, or larvicidal efficacy was not specifically evaluated in this assay.

The steep increase in mortality observed at intermediate concentrations suggests a threshold effect, in which the accumulation of active compounds reaches a critical level capable of inducing rapid disruption of cellular homeostasis. This behavior is consistent with the known mechanisms of action of terpene-rich essential oils, including membrane destabilization, increased permeability, and interference with respiratory and metabolic processes.^36,59^

Optical microscopy analysis (Figure 5C–J) revealed pronounced morphological differences between treated and control larvae. Larvae exposed to nGDs (Figure 5C–F) exhibited clear structural alterations, including tissue disorganization and deformation of anatomical regions. In contrast, control larvae (Figure 5G–J) showed preserved anatomical features, including intact head, thorax, abdominal segments, and siphon.

Additionally, significant structural damage was observed in treated larvae, including apparent collapse of internal tissues and disruption of structural integrity. These alterations indicate severe physiological impairment, likely affecting essential processes such as respiration, osmoregulation, and digestion.^60^ The observed coating-like appearance in treated larvae may also suggest a physical contribution of the nanogel matrix, enhancing the interaction between bioactive compounds and the larval surface.

Overall, the results indicate that nGDs exhibit larvicidal activity against *A. aegypti*, with a well-defined concentration–response profile (Figure 5A) and associated structural damage at the organism level (Figure 5C–F). These findings suggest that the nanogel acts as a carrier system for EODs and exhibits larvicidal activity under the tested conditions. Further studies comparing free EODs and nGDs are required to determine whether incorporation into the nanogel modifies the larvicidal potency of the essential oil.

It should be noted that the larvicidal assay was performed using an adapted WHO-based protocol. The use of 10 larvae in 20 mL of test solution, with 10 replicates per concentration, differs from the standard WHO design regarding larvae number per replicate, exposure volume, and experimental unit. Although the total number of larvae per concentration was maintained at 100, these methodological differences may influence larval exposure, formulation dispersion, mortality estimates, and LC_50_/LC_90_ calculations. Therefore, the larvicidal results should be interpreted under the adapted experimental conditions used in this study, and direct comparison with standardized WHO assays should be made with caution.

### In Vitro Leishmanicidal Activity of nGDs

The leishmanicidal activity of *D. stelechantha* essential oil (EODs) and the EODs-loaded nanogel (nGDs) was evaluated against *L. amazonensis* promastigotes after 24 and 48 h of exposure. Cell mortality data and IC_50_ values (Table 7) clearly indicate that free EODs exhibit higher immediate leishmanicidal potency compared to the nanogel formulation. After 24 h, EODs showed an IC_50_ of 276.5 ± 41.72 μg/mL, whereas nGDs presented a higher IC_50_ (493.45 ± 0.49 μg/mL), indicating lower immediate activity under the tested conditions.

**Table 7:** Leishmanicidal Activity and IC_50_ Values of nGDs and EODs against *L. amazonensis* after 24 and 48 h of Exposure^a^

	nGDs	EODs
parameter	cell mortality (%)—24 h	
505 μg/mL	80.25 ± 1.06	95.00 ± 0.00
336.5 μg/mL	0.00 ± 0.00	94.50 ± 4.24
224.4 μg/mL	0.00 ± 0.00	
	Cell mortality (%)—48 h	
505 μg/mL	93.75 ± 1.77	97.15 ± 0.49
336.5 μg/mL	2.50 ± 3.54	95.95 ± 3.46
224.4 μg/mL	0.00 ± 0.00	
	IC_50_ (μg/mL) [95% CI]	
24 h	493.45 ± 0.49	276.50 ± 41.72
	[492.46–494.15]	[193.06–335.50]
48 h	448.85 ± 51.83	304.80 ± 2.83
	[345.19–522.15]	[299.14–308.80]

a Concentrations are expressed as EODs-equivalent concentration (μg/mL). Data are expressed as mean ± SD, with 95% confidence intervals (95% CI).

This behavior is consistent with the concentration–response profile shown in Figure 6, in which free EODs exhibited higher immediate leishmanicidal activity than nGDs, particularly at intermediate concentrations. In contrast, nGDs showed a delayed response under the tested conditions, with increased promastigote mortality mainly at the highest concentration and after longer exposure.

**Figure 6:**
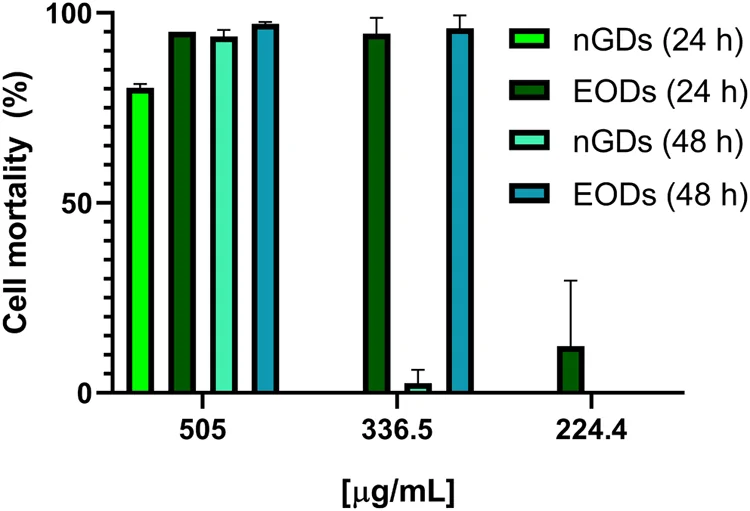
Leishmanicidal activity of EODs and the EODs-loaded nanogel (nGDs) against *L. amazonensis* promastigotes after 24 and 48 h of exposure. Cell mortality (%) is presented as a function of EODs-equivalent concentration (μg/mL). EODs exhibited higher immediate activity, whereas nGDs showed a delayed response under the tested conditions. This behavior may be associated with formulation-dependent modulation of EODs availability, although controlled release was not directly evaluated. Data are expressed as mean ± SD.

This delayed response may be associated with the physicochemical properties of the nanogel system. FTIR analysis demonstrated that the incorporation of EODs modulates intermolecular hydrogen bonding within the polymeric network, leading to structural reorganization and the formation of a more cohesive matrix. In parallel, DLS results revealed that nGDs maintains relatively stable particle sizes across the evaluated temperature range, with increased diffusion coefficients at higher temperatures. Together, these findings suggest that the nanogel structure may influence the availability of EODs over time. However, controlled release cannot be confirmed without specific release kinetics studies. Furthermore, rheological analysis showed that nGDs exhibits higher viscosity, greater gel strength, and enhanced viscoelastic behavior compared to the nG, indicating a more structured and stable network. This increased structural organization may restrict the immediate availability of bioactive compounds, which could contribute to the lower initial activity observed at 24 h. The slight increase in activity observed over time may reflect gradual changes in the availability of EODs within the system, although this hypothesis requires confirmation through release assays.

Morphological analysis (Figure 7) further corroborates these findings. After 24 h, parasites treated with EODs showed early structural damage, including cell rounding, flagellar disruption, and reduced cell density, indicating rapid membrane interaction and cytotoxicity. In contrast, nGDs-treated parasites exhibited milder alterations at this stage, consistent with a slower biological response. After 48 h, however, both treatments resulted in pronounced morphological damage, including deformation, aggregation, and extensive cellular debris, indicating loss of membrane integrity and progression toward cell death.

**Figure 7:**
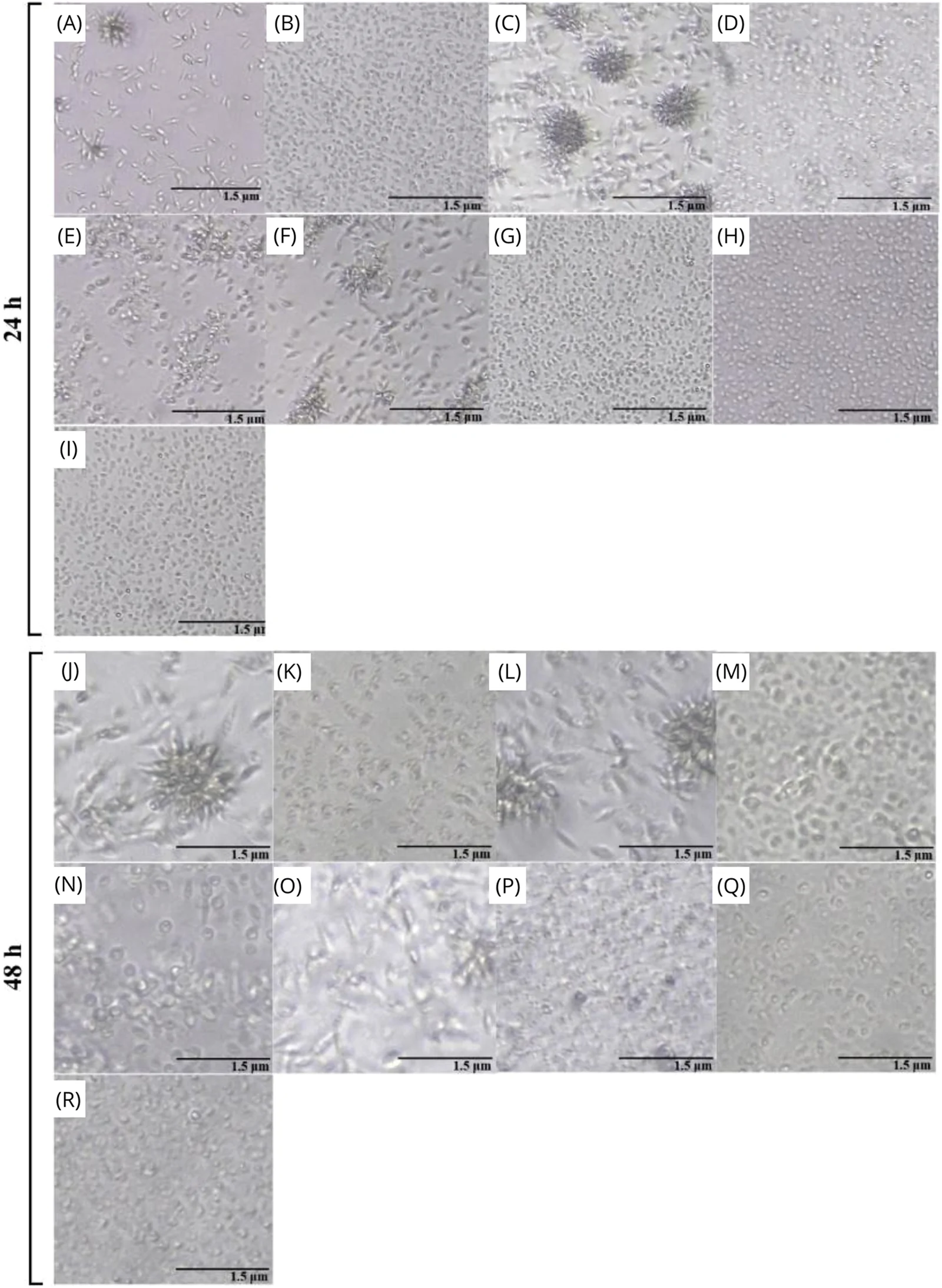
Optical micrographs of *Leishmania (Leishmania) amazonensis* promastigotes after exposure to EODs and the EODs-loaded nanogel (nGDs) at 24 and 48 h. (A) Untreated culture (negative control); (B) amphotericin B (1.95 μg/mL; positive control); (C) blank nanogel (vehicle control); (D–F) nGDs at 505, 336.5, and 224.4 μg/mL, respectively, after 24 h; (G–I) EODs at the same concentrations after 24 h; (J–R) corresponding treatments after 48 h. Scale bars: 1.5 μm. Treated promastigotes exhibited marked morphological alterations, including cell shrinkage, flagellar damage, and reduced cell density, particularly at higher concentrations and longer exposure times.

Overall, these results suggest that the nanogel structure may influence the time-dependent biological response observed for nGDs. While free EODs exhibited higher immediate leishmanicidal activity, nGDs showed a more delayed response under the tested conditions. This behavior may be relevant for future formulation studies, but additional cytotoxicity, release, and efficacy assays are required before therapeutic advantages can be inferred. These findings highlight the potential of nGDs as a carrier system for plant-derived bioactive compounds, while indicating the need for further studies to confirm release behavior and biological advantages over the free essential oil.

Future studies should include cytotoxicity assessment in mammalian cells, determination of encapsulation efficiency, controlled release assays, and mechanistic investigations to better define the safety profile, release behavior, and mode of action of the EODs-loaded nanogel. These complementary assays will be important to confirm the functional role of the nanogel matrix and to support further development of this system for antiparasitic and vector control applications.

## Conclusions

A thermoresponsive nanogel based on Pluronic F127 and Carbopol 974P was successfully developed for the incorporation of *D. stelechantha* essential oil (EODs), resulting in a stable and structurally organized system (nGDs). Physicochemical characterization confirmed that the incorporation of EODs modulated intermolecular interactions, improved rheological properties, and maintained temperature-responsive behavior, with a sol–gel transition near physiological conditions.

Biological assays demonstrated that free EODs exhibited higher immediate leishmanicidal activity, whereas nGDs showed a delayed time-dependent response. This behavior may be related to the organization of the nanogel matrix, but controlled release studies are required to confirm this mechanism. Morphological analysis confirmed significant structural damage in treated parasites. In addition, nGDs showed strong larvicidal activity against *A. aegypti*, with well-defined dose–response behavior and high efficacy at relatively low concentrations.

Overall, the developed nanogel provides a stable platform for incorporating EODs and maintaining biological activity under the tested conditions. This system represents a promising natural-based platform for further investigation in antiparasitic and vector control applications, particularly after additional release, cytotoxicity, and temperature-dependent biological studies.

## Supplementary Material



## Notes

GC–MS total ion chromatogram, mass spectra of the identified compounds, the complete chemical composition table of EODs, NMR spectra (^1^H, ^13^C, and DEPT 135), and FTIR spectra of the blank and EODs-loaded nanogel formulations (PDF)
