# Supplementary material for: Thermoresponsive Nanogel Incorporating Duguetia stelechantha Essential Oil: Biological Activity against Leishmania and Aedes aegypti Larvae
Source: ACS Omega. 2026 Aug 26;11(35):52796–809. doi: 10.1021/acsomega.6c04271 (PMC13563625; doi:10.1021/acsomega.6c04271)

Supporting Information

Thermoresponsive nanogel incorporating *Duguetia stelechantha* essential oil: biological activity against *Leishmania* and *Aedes aegypti* larvae

Raiene Lisboa Rocha[1], Arthur Girardi Francalin Laureano[2], Thaíssa Gabrielle Corrêa[1], Melissa Pires Souza[3], Anderson Ferreira Sepulveda[4], Agatha Maria Pelosine[4], Clenilma Marques Brandão[5], Gustavo Braga[6], Carlos Alexandre Holanda[7], Daniele Stéfanie Sara Lopes Lera-Nonose[2], Daniele Ribeiro de Araujo[4], Emmanoel Vilaça Costa[3], Renato Sonchini Gonçalves[8]*

[1] Laboratory of Chemistry of Natural Products, Department of Chemistry, Federal University of Maranhão (UFMA), São Luís 65080-805, Brazil;
[2] Department of Clinical Analysis and Biomedicine, State University of Maringá (DAB/UEM), Brazil;
[3] Postgraduate Program in Chemistry, Federal University of Amazonas (UFAM), Manaus 69080-900, Brazil;
[4] Department of Biophysics, Federal University of São Paulo (UNIFESP), São Paulo, SP, Brazil;
[5] Department of Chemistry, Federal Institute of Maranhão (IFMA), São Luís 65075-441, Brazil;
[6] University College (COLUN), Federal University of Maranhão (UFMA), São Luís 65080-805, Brazil;
[7] Natural Sciences Degree Coordination, Federal University of Maranhão, Bom Jesus campus, Imperatriz, MA 65915-060, Brazil;
[8] Department of Engineering and Exact Sciences, Palotina Sector, Federal University of Paraná (UFPR), Palotina 85950-000, PR, Brazil.

***Corresponding author: renato.sonchini@ufpr.br**

Figure S1. (**A**) Chromatogram of the total ions of *Duguetia stelechanta*; (**B**) Enlargement of the region between 10.0 min to 50.0 min.

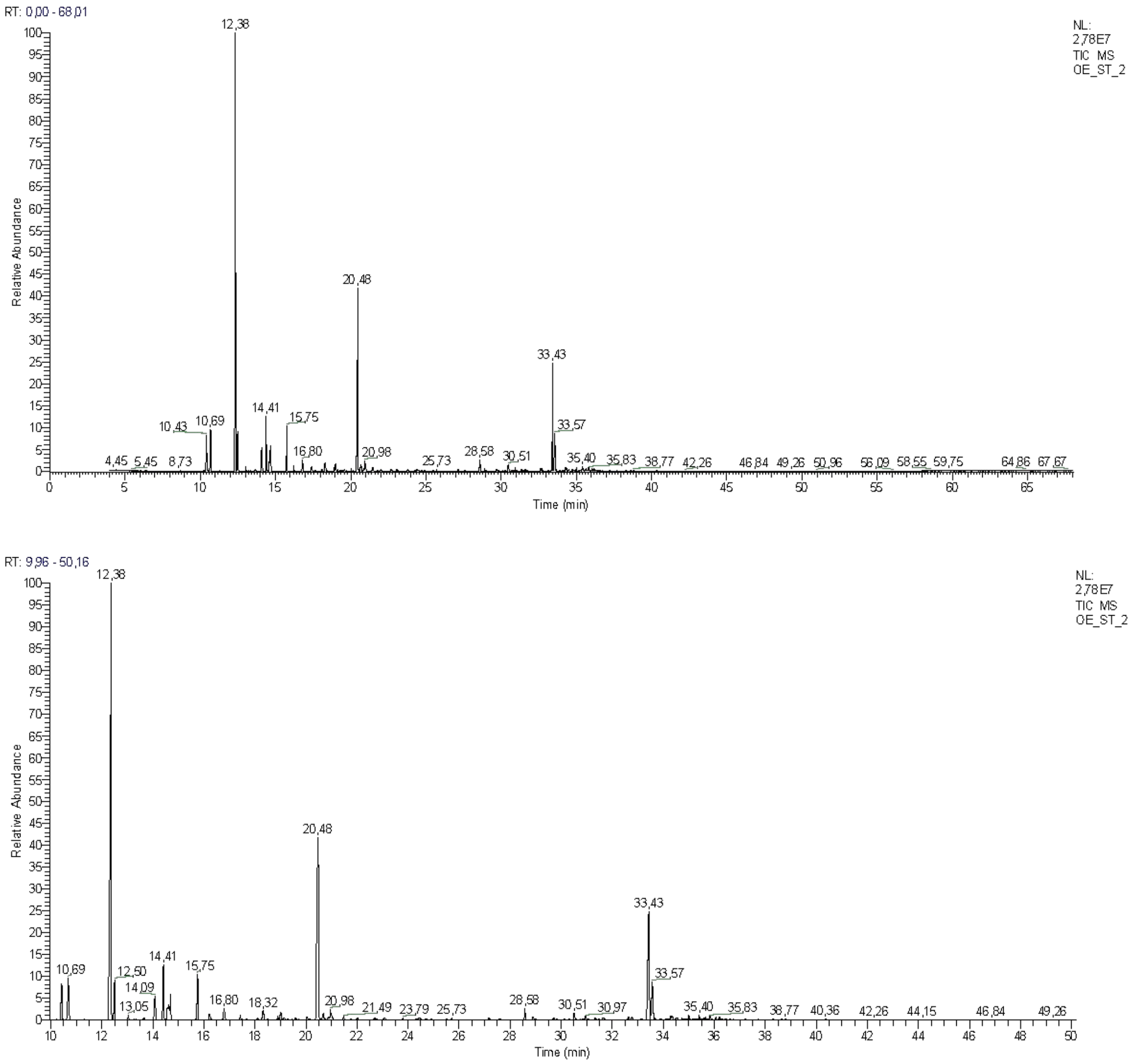

Figure S2. Mass spectrum of α-thujene (t_R 10.43 min).

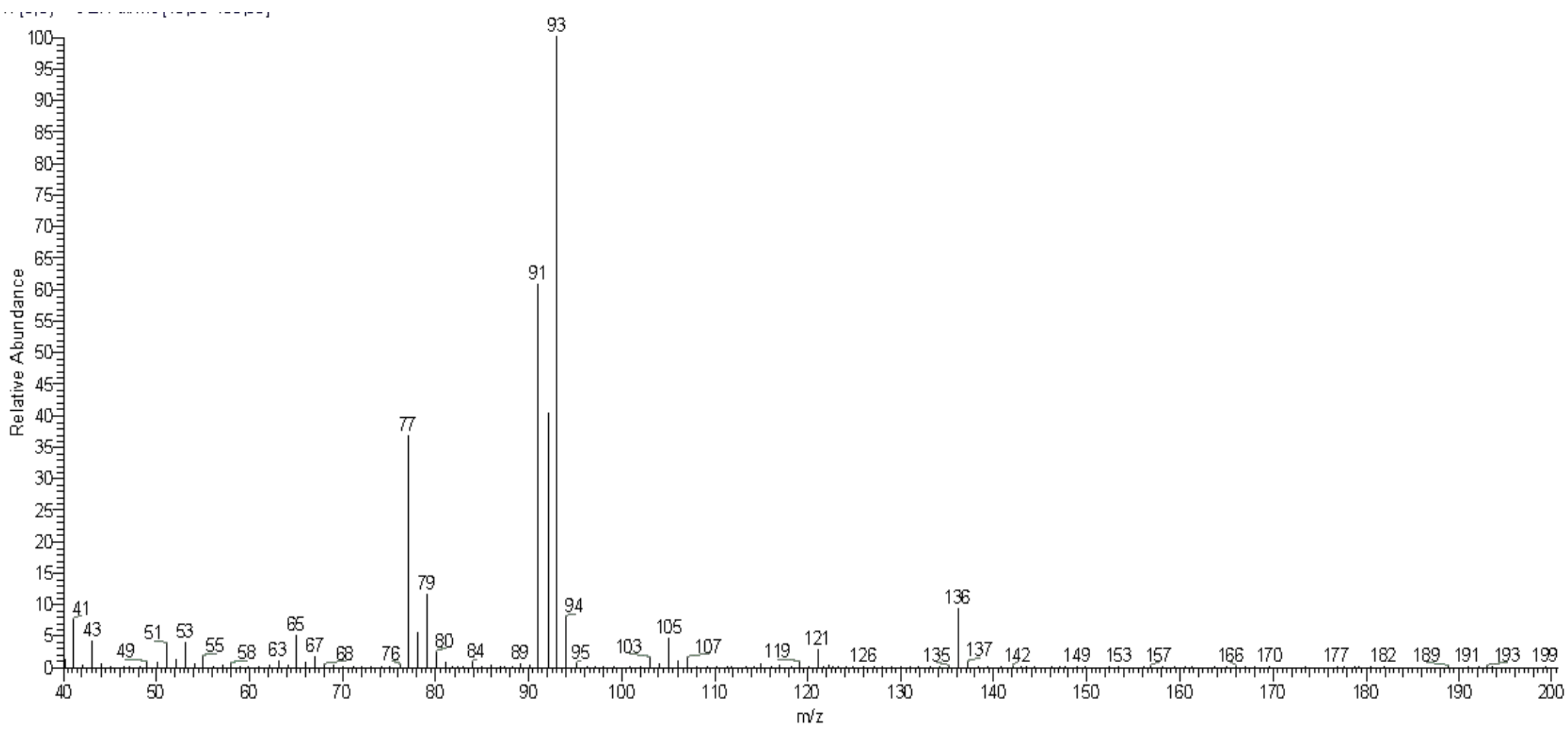

Figure S3. Mass spectrum of α-pinene (t_R 10.69 min).

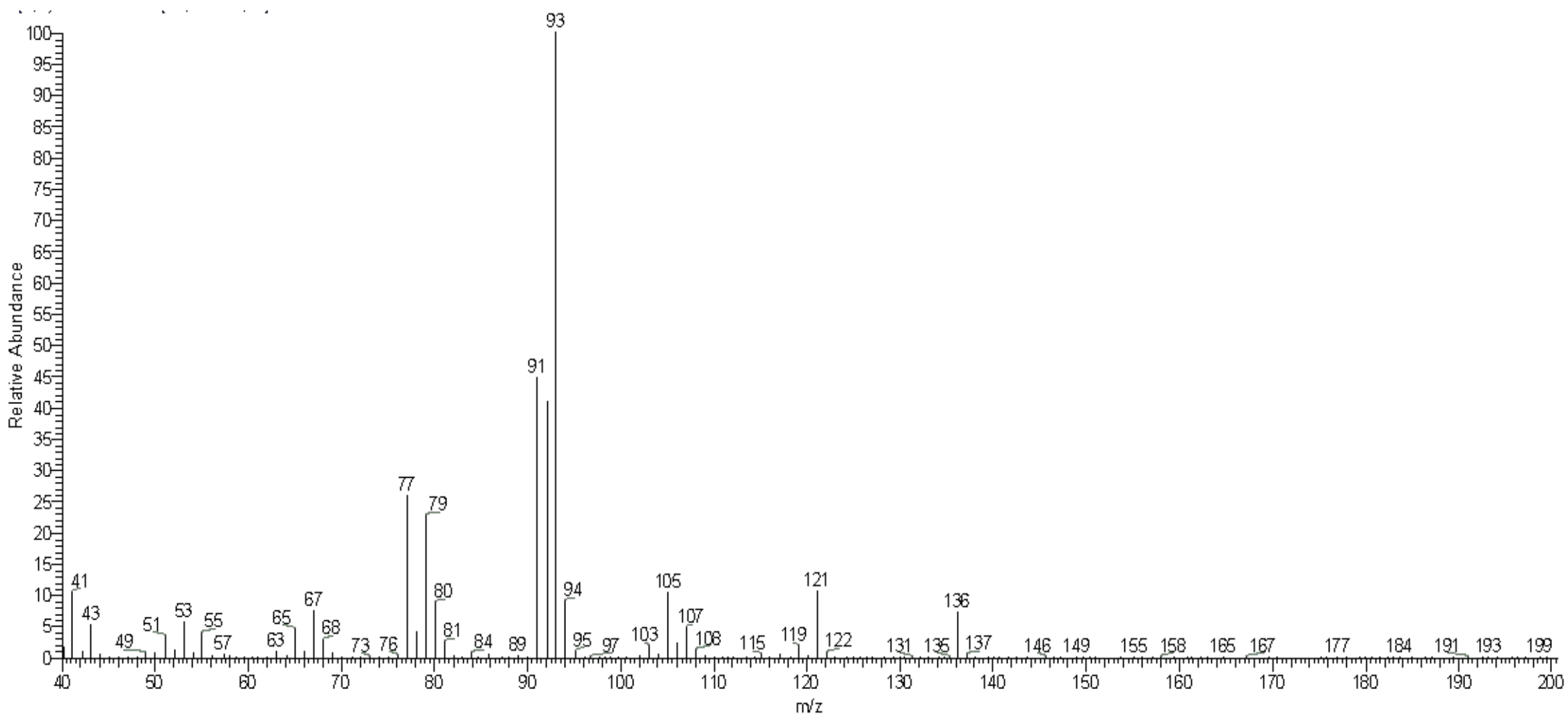

Figure S4. Mass spectrum of Sabinene (t_R 12.38 min).

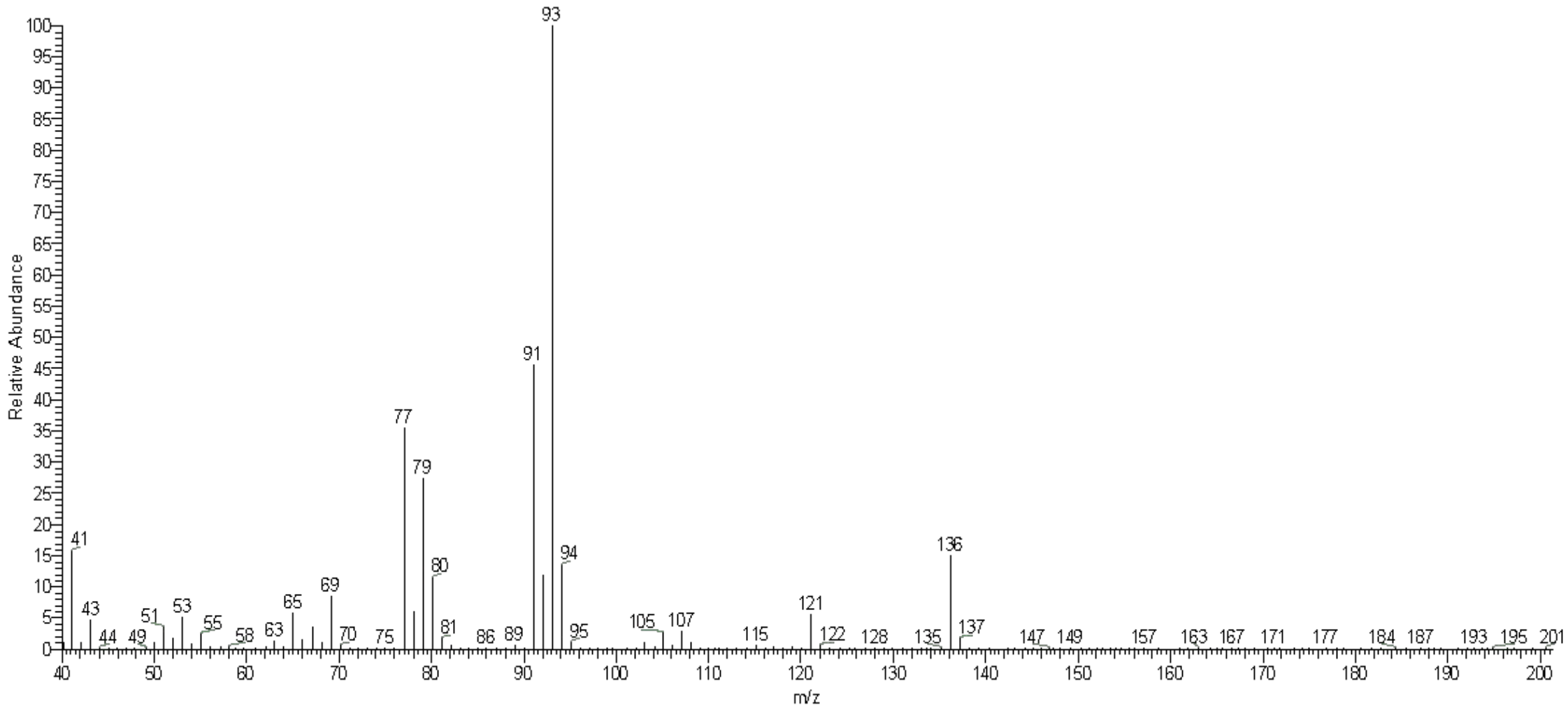

Figure S5. Mass spectrum of β-pinene (t_R 12.50 min).

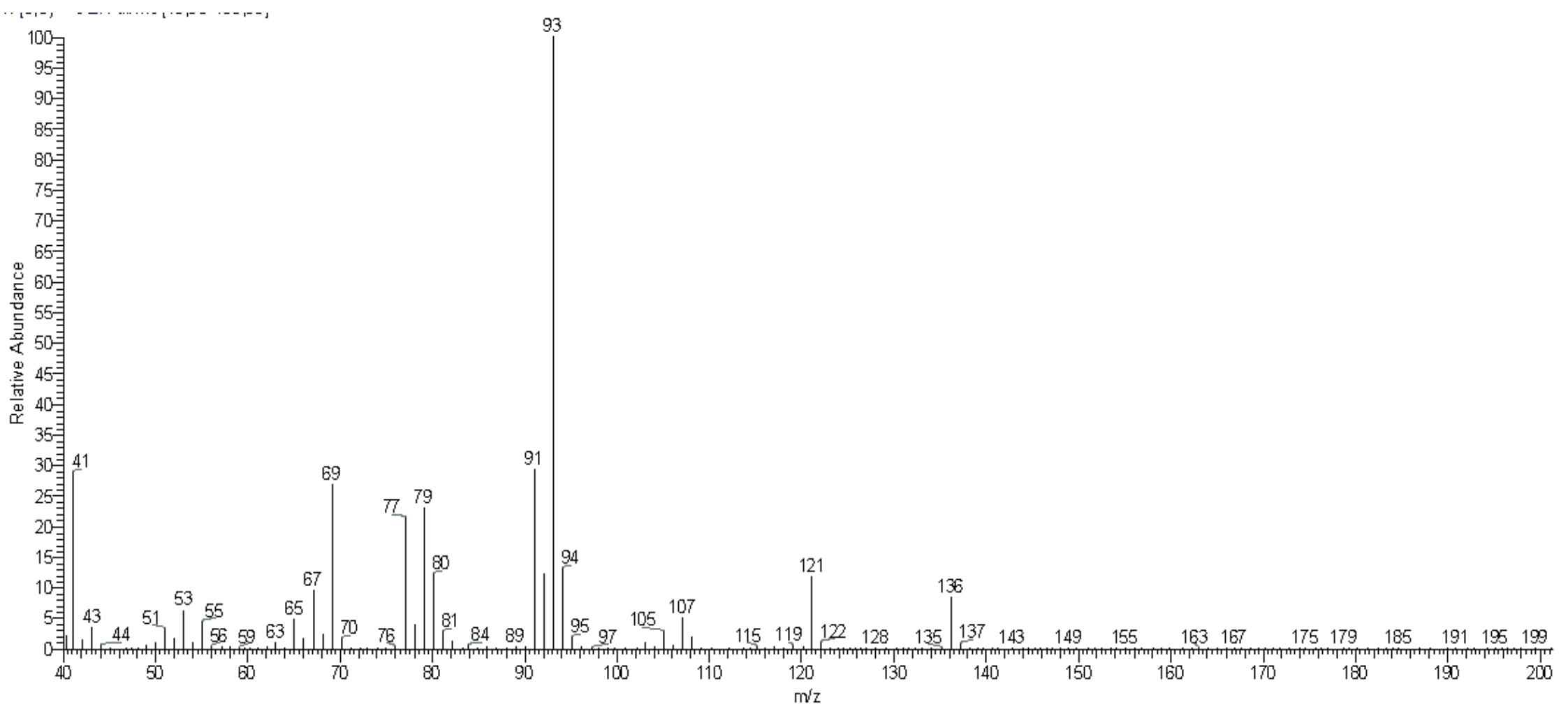

Figure S6. Mass spectrum of Myrcene (t_R 13.05 min).

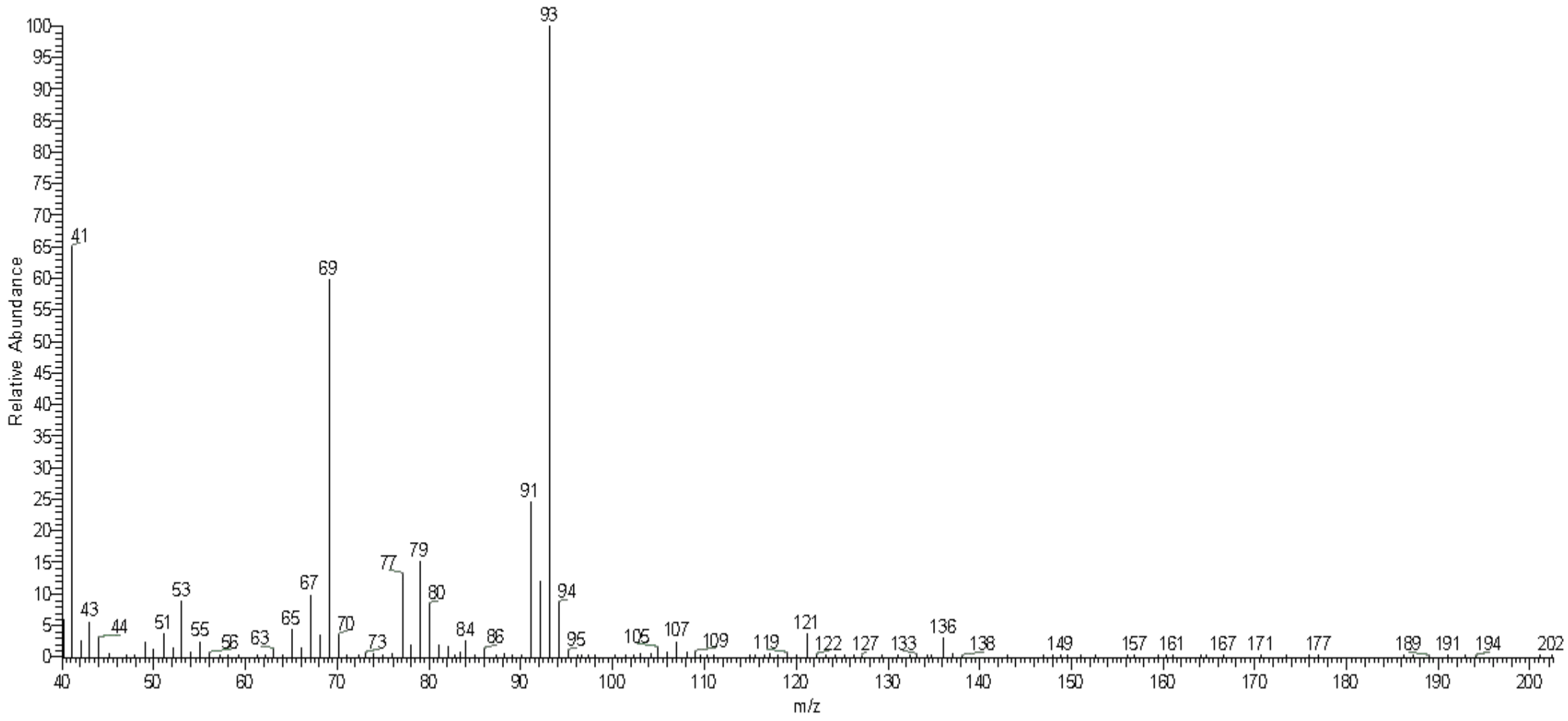

Figure S7. Mass spectrum of α-phellandrene (t_R 13.67 min).

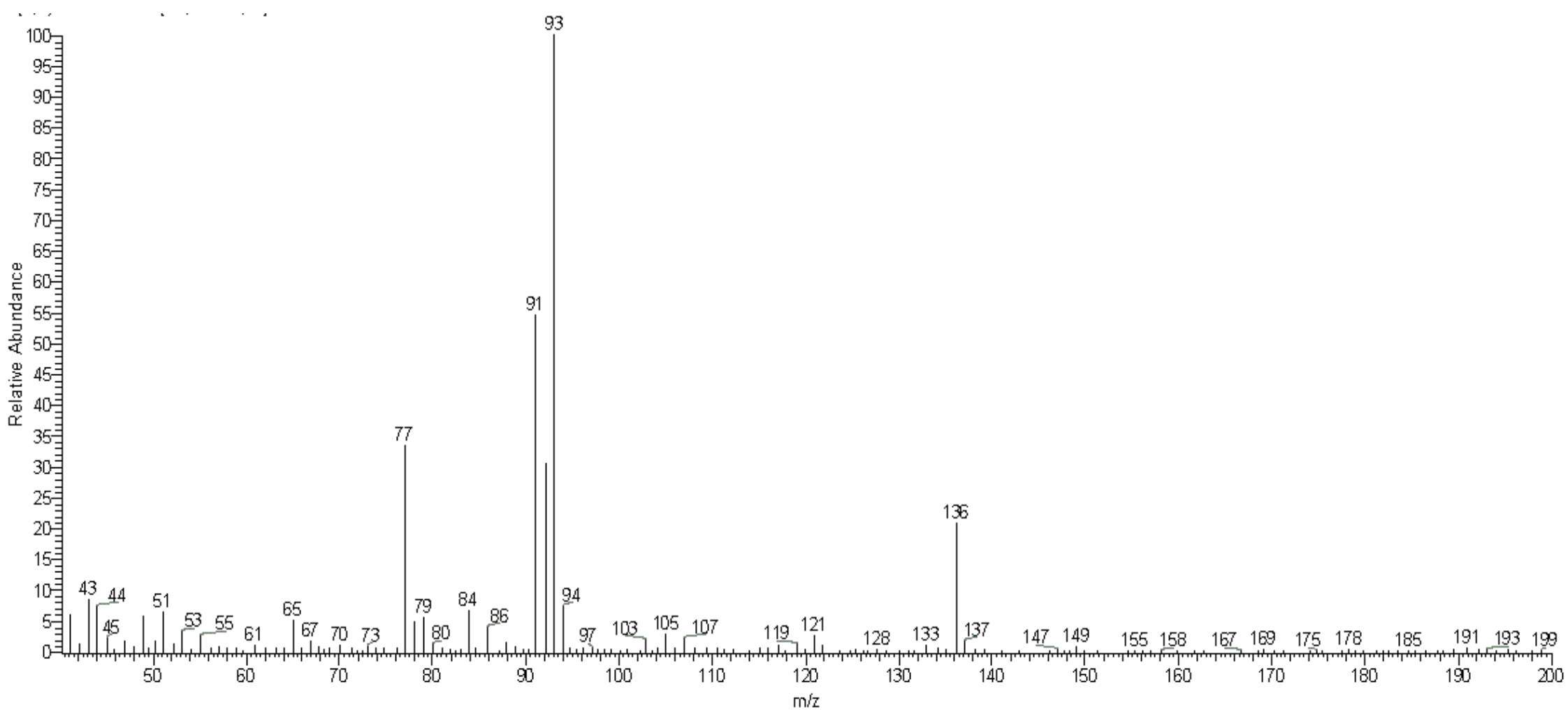

Figure S8. Mass spectrum of α-terpinene (t_R 14.09 min).

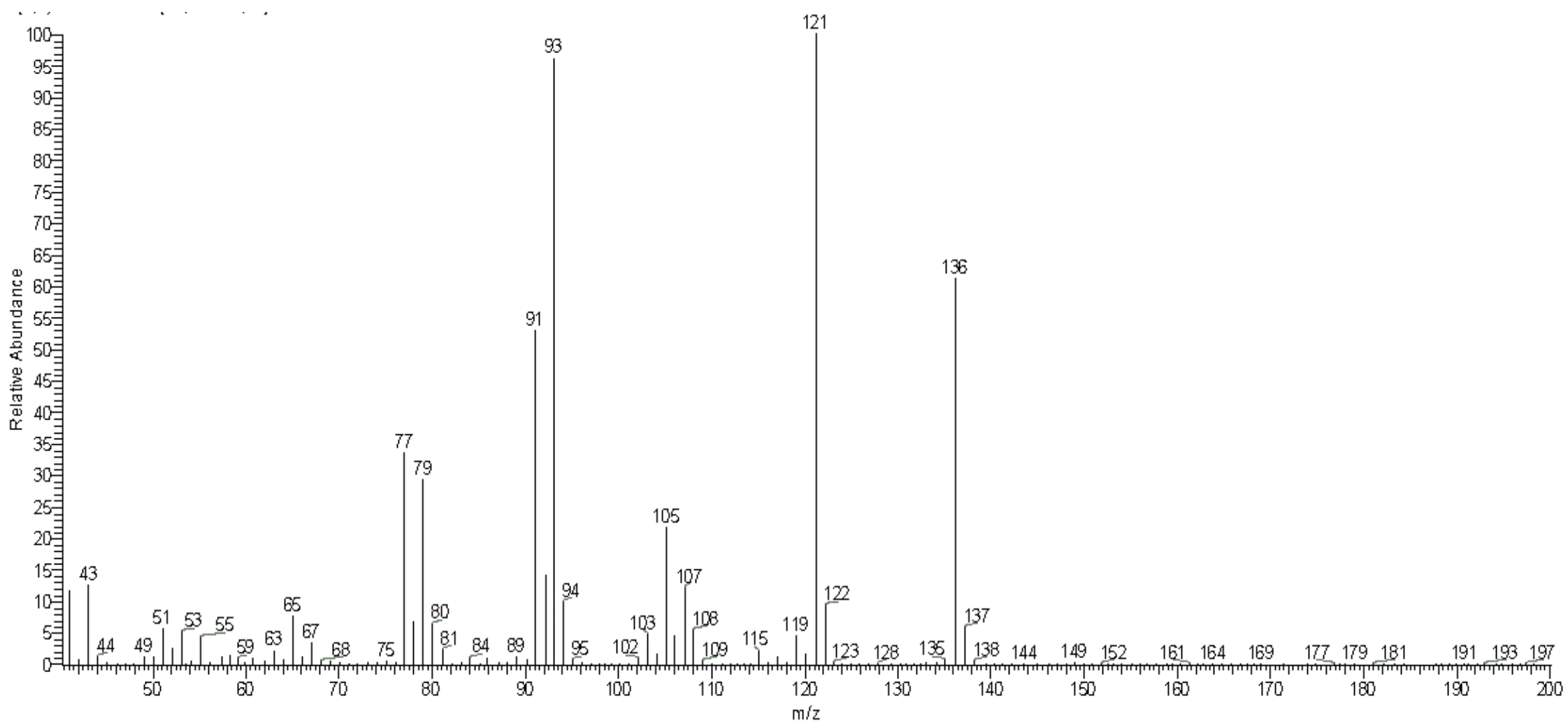

Figure S9. Mass spectrum of *p*-cymene (t_R 14.41 min).

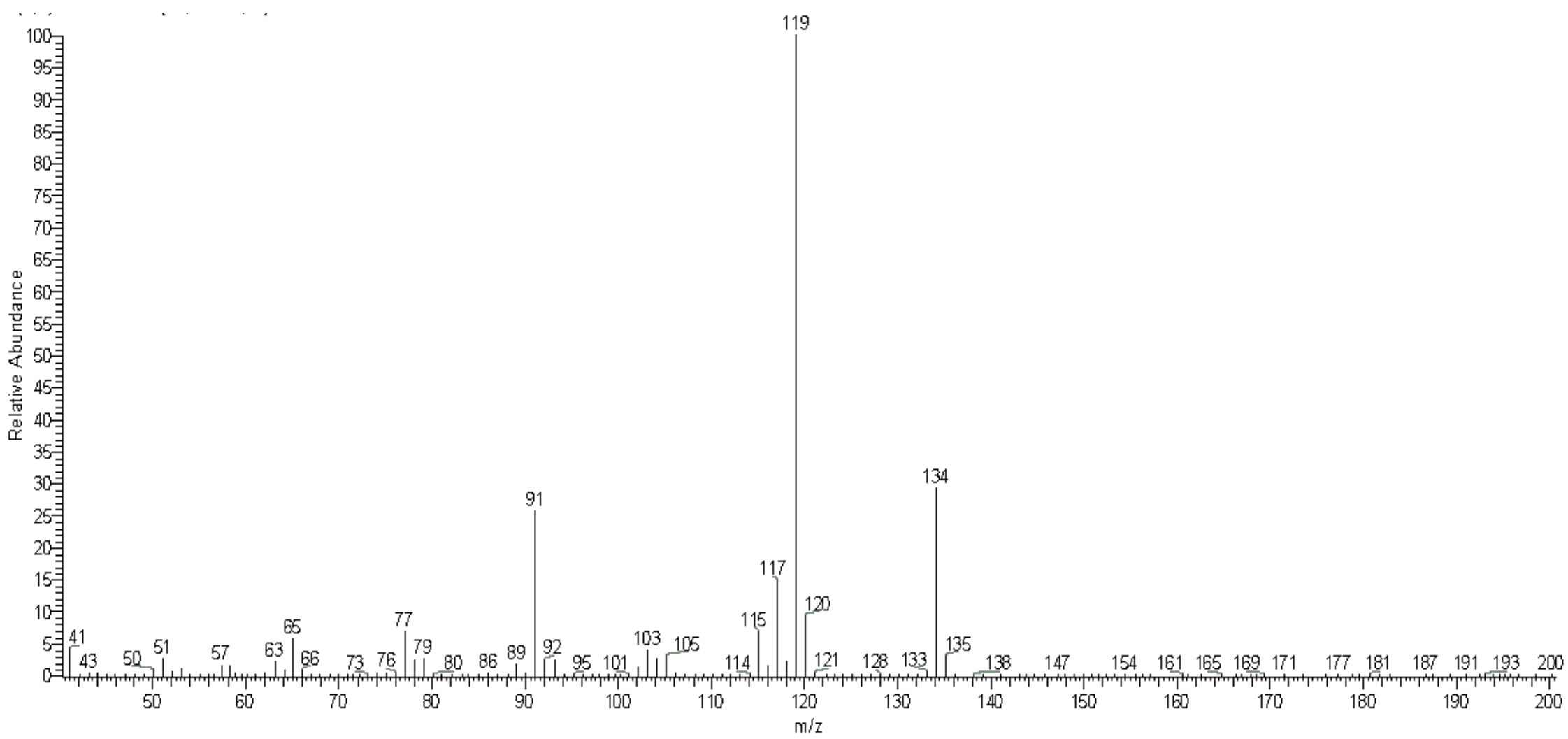

Figure S10. Mass spectrum of Limonene (t_R 14.59 min).

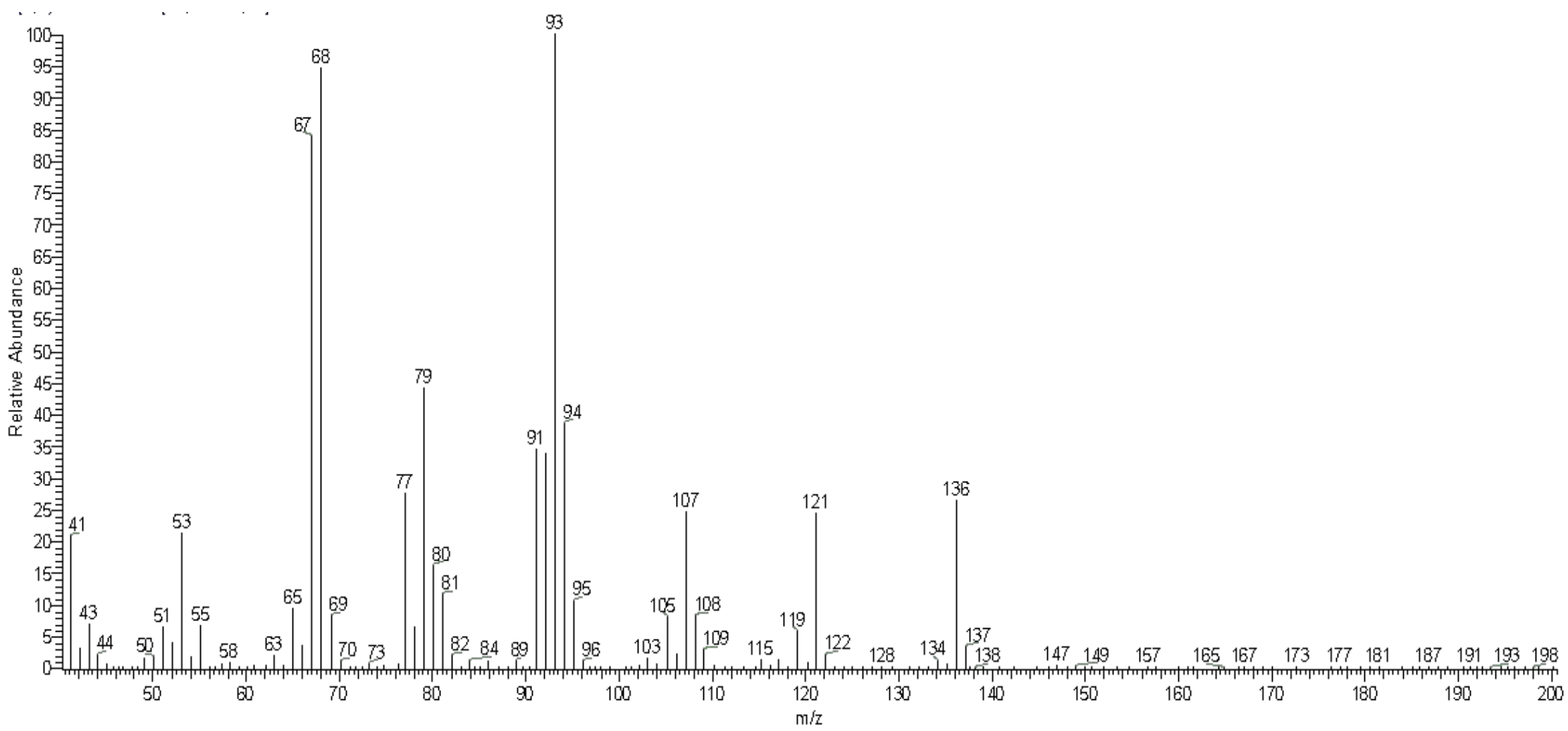

Figure S11. Mass spectrum of β-phellandrene (t_R 14.63 min).

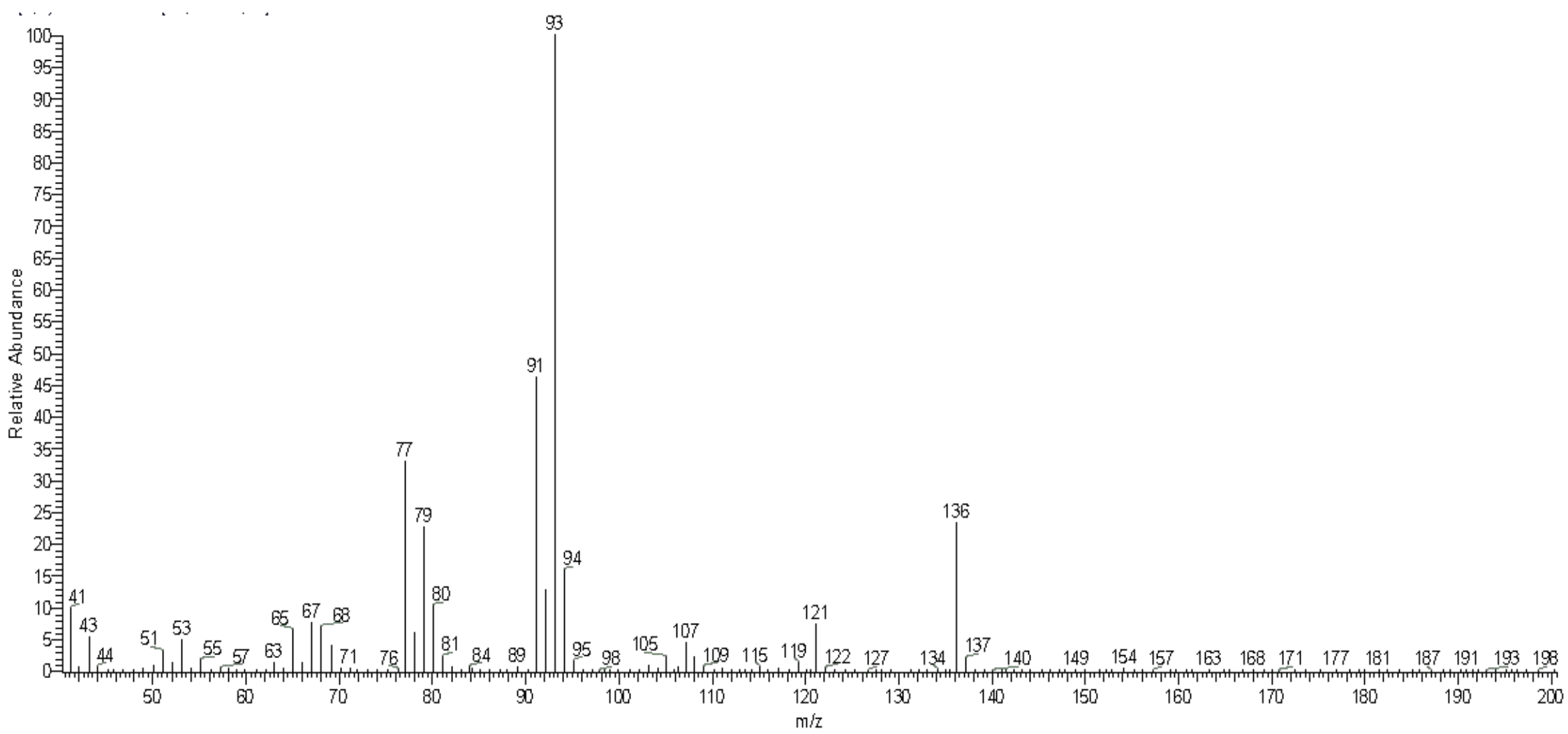

Figure S12. Mass spectrum of 1,8-cineole (t_R 14.70 min).

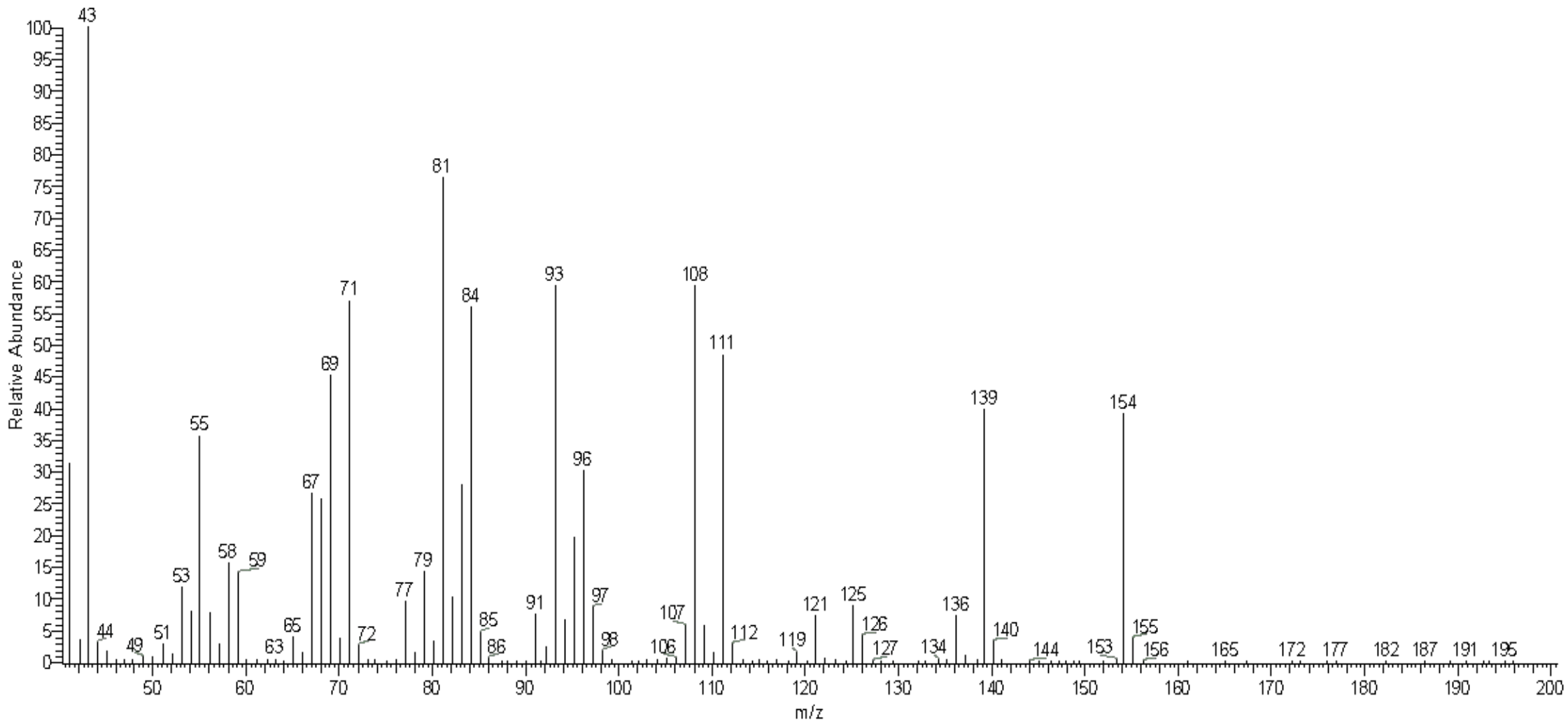

Figure S13. Mass spectrum of *γ*-terpinene (t_R 15.75 min).

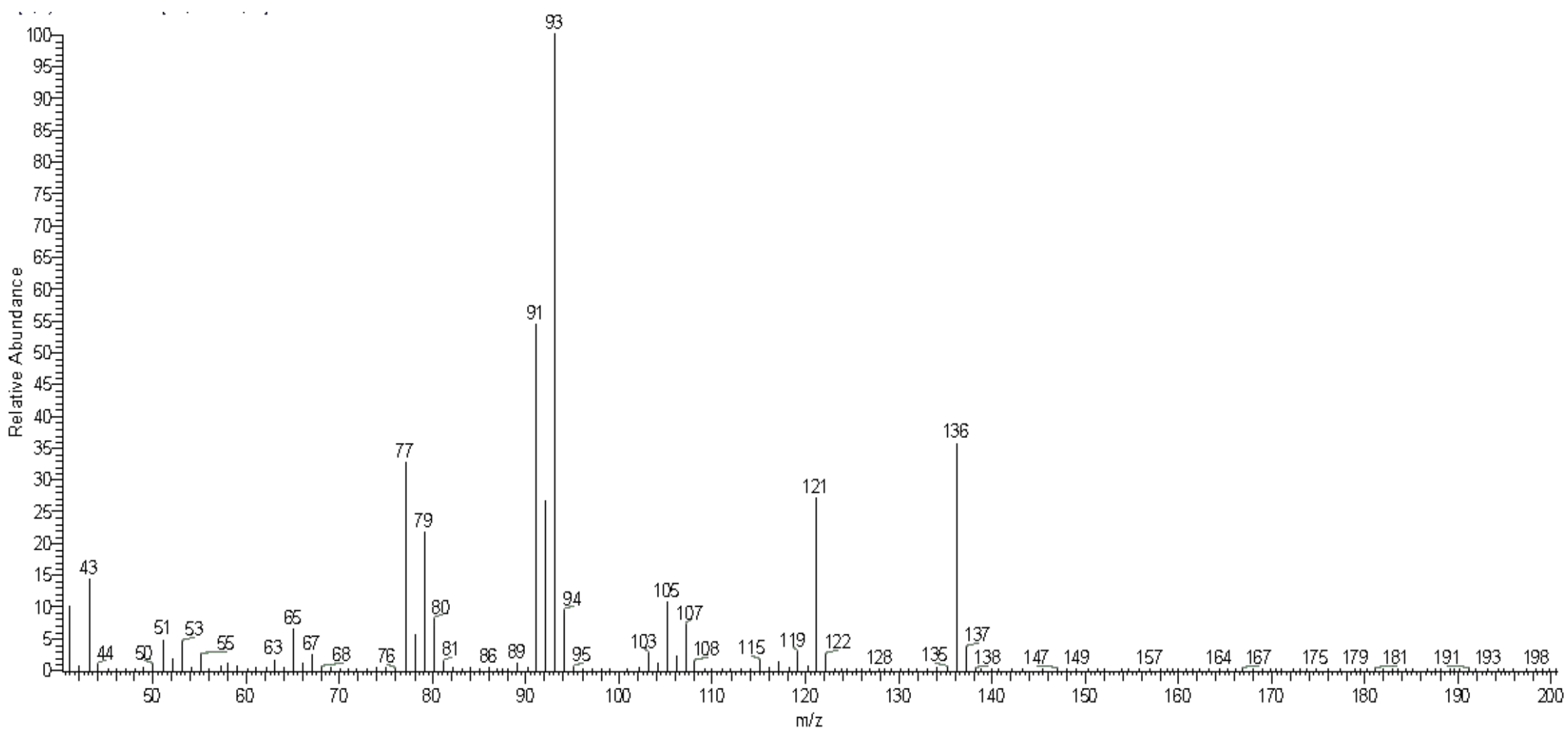

Figure S14. Mass spectrum of Sabinene hydrate <cis-> (IPP vs. OH) (t_R 16.23 min).

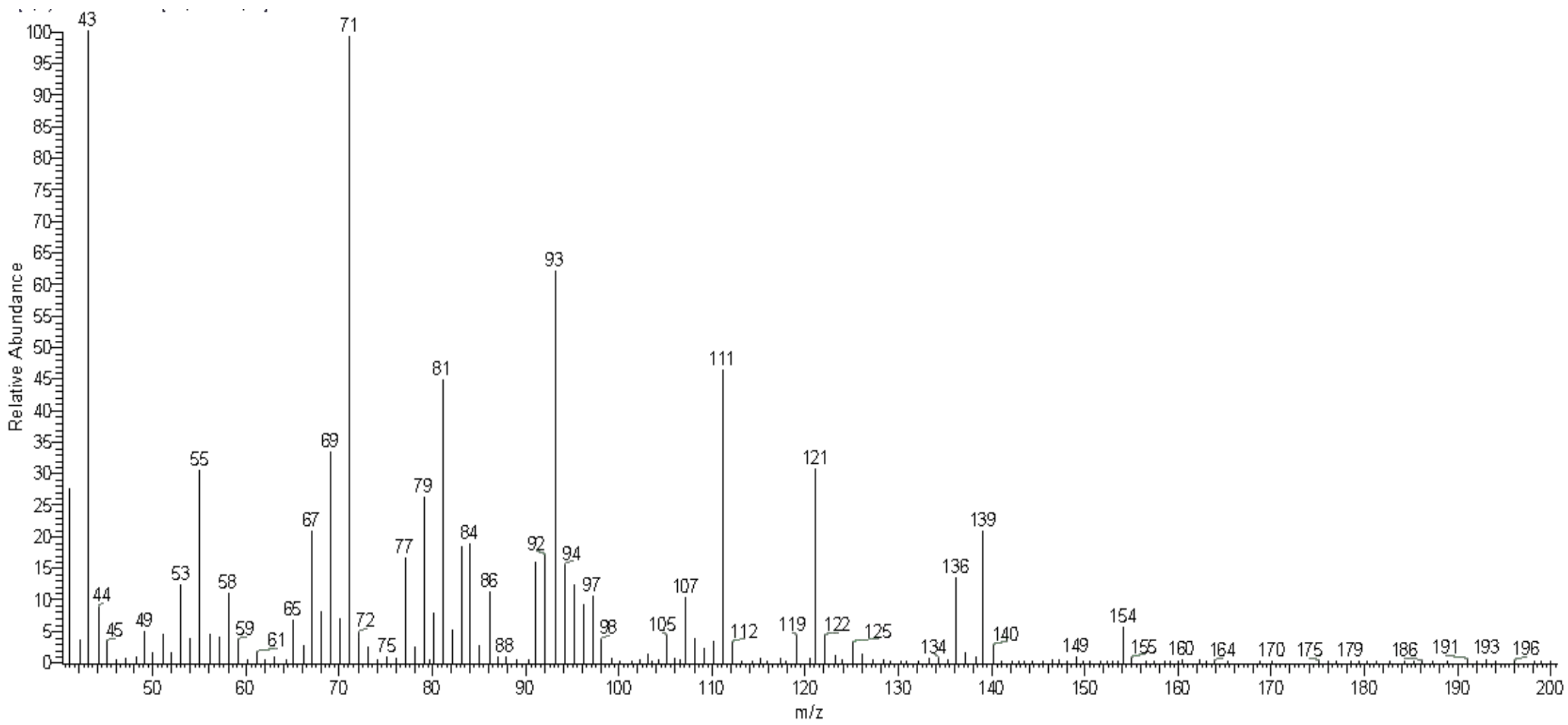

Figure S15. Mass spectrum of Terpinolene (t_R 16.80 min).

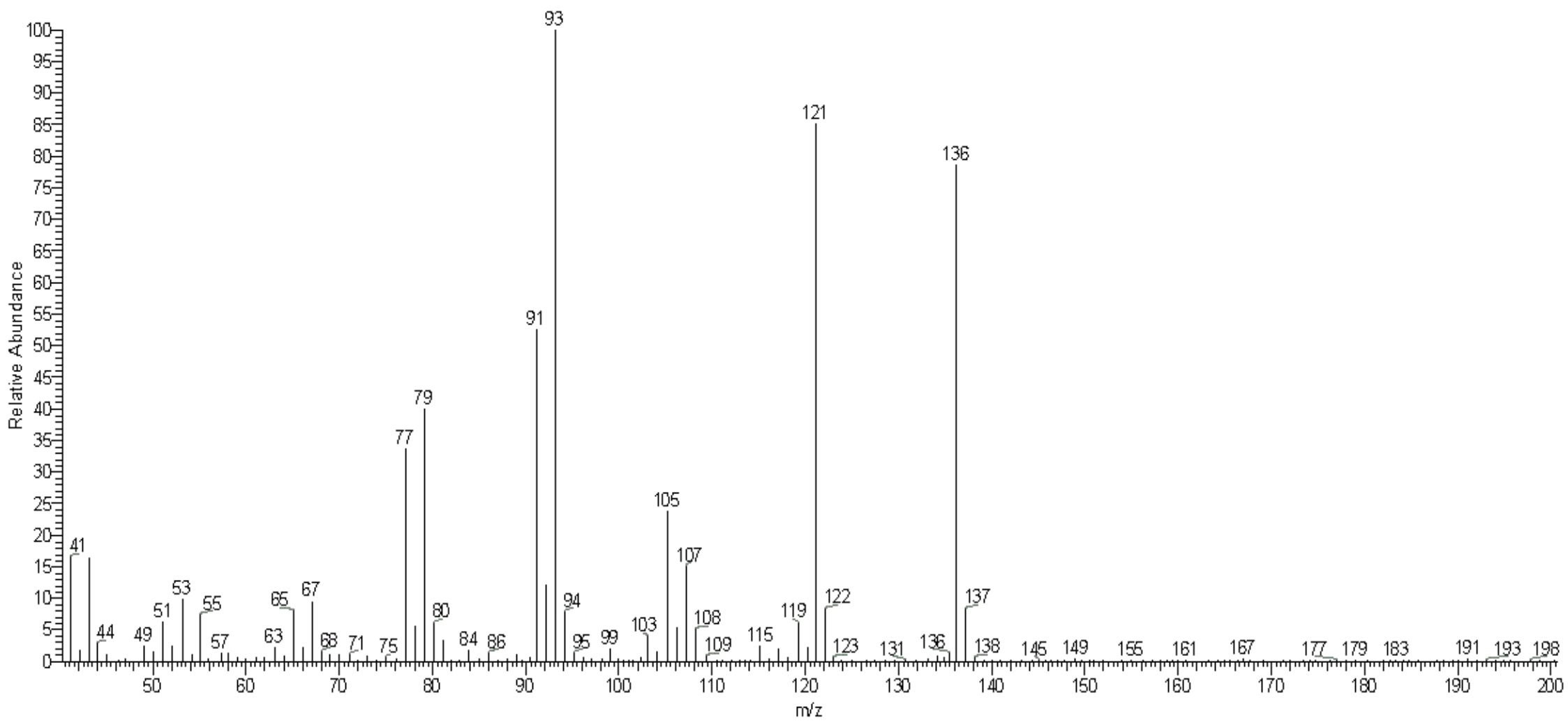

Figure S16. Mass spectrum of Sabinene hydrate <trans-> (IPP vs. OH) (t_R 17.43 min).

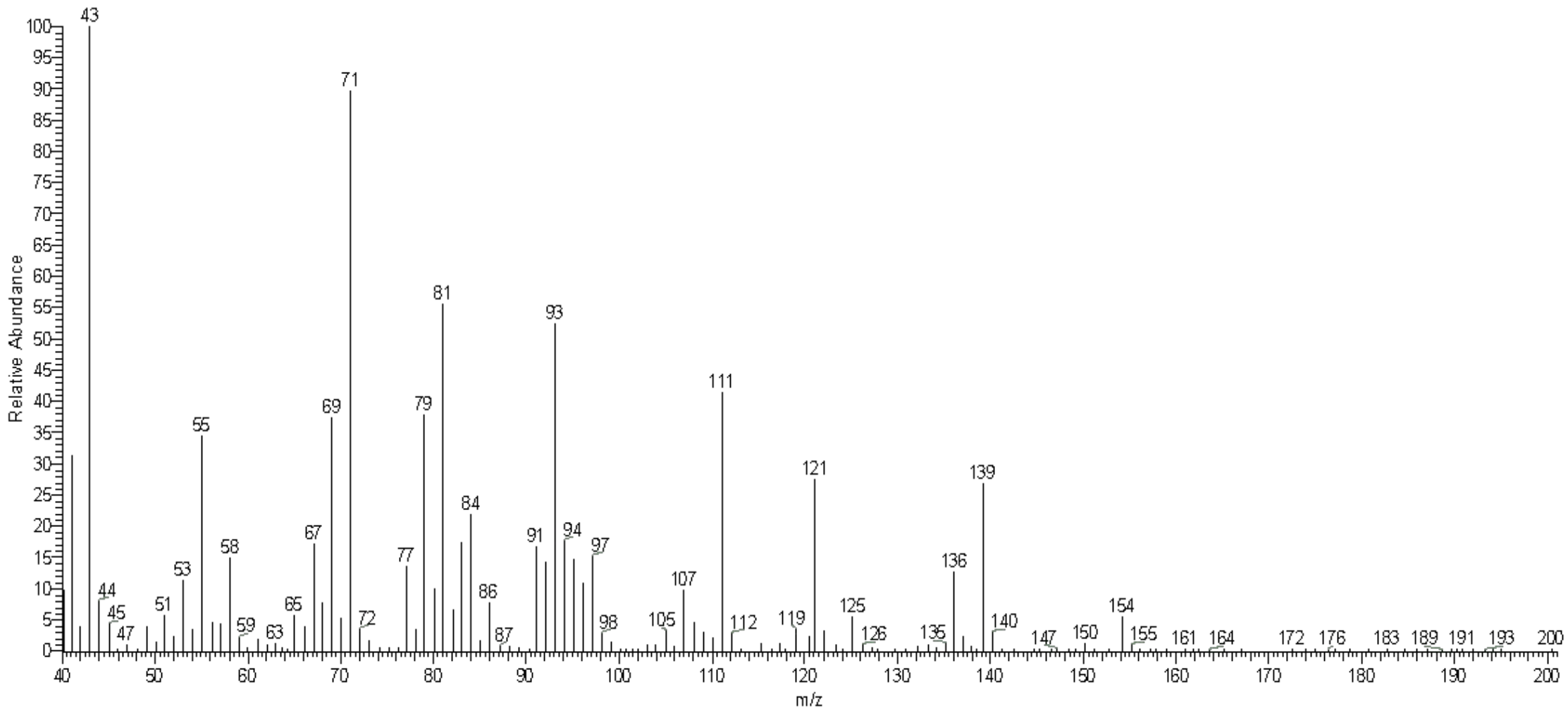

Figure S17. Mass spectrum of Fenchol <endo-> (t_R 18.11 min).

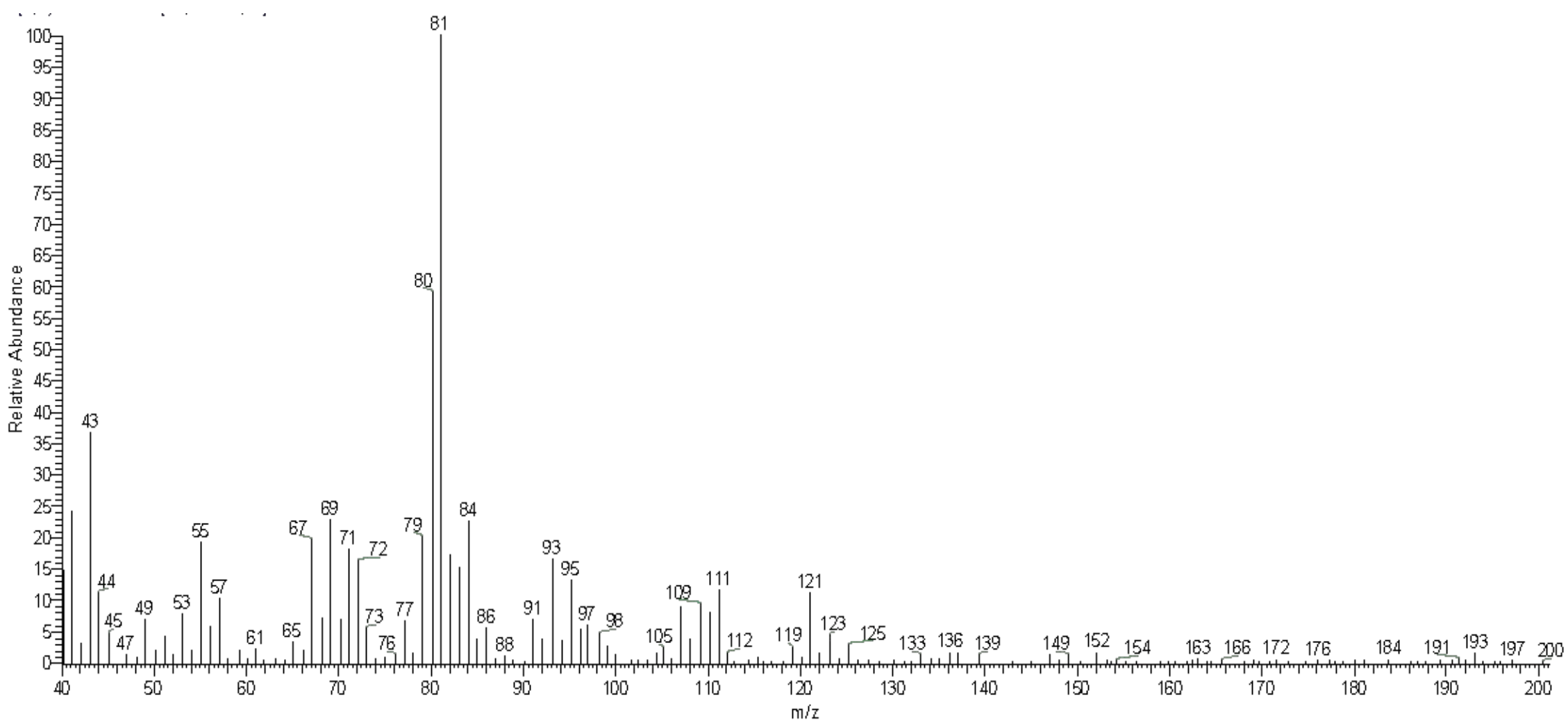

Figure S18. Mass spectrum of Menth-2-en-1-ol <cis-ρ-> (t_R 18.32 min).

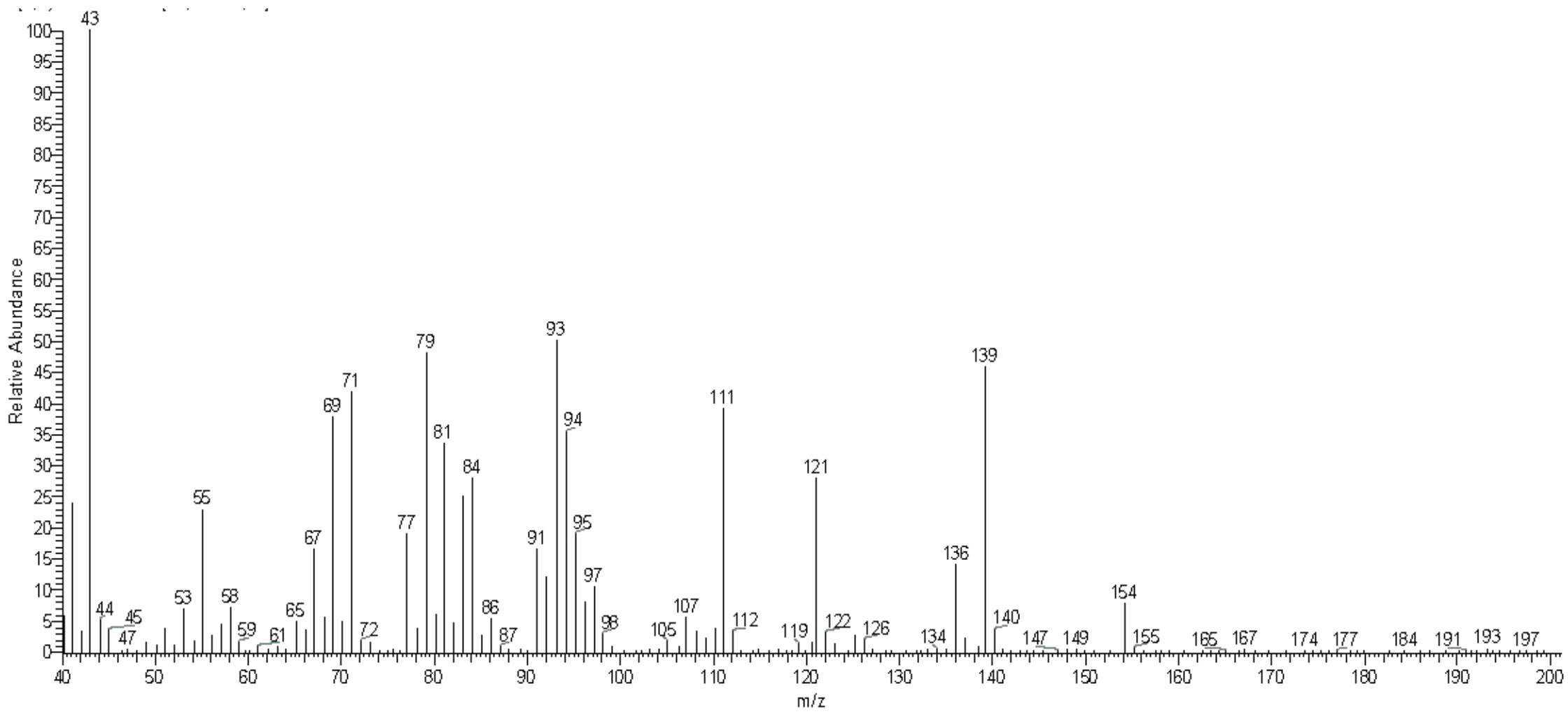

Figure S19. Mass spectrum of Sabinol <trans-> (trans for OH vs. IPP) (t_R 18.91 min).

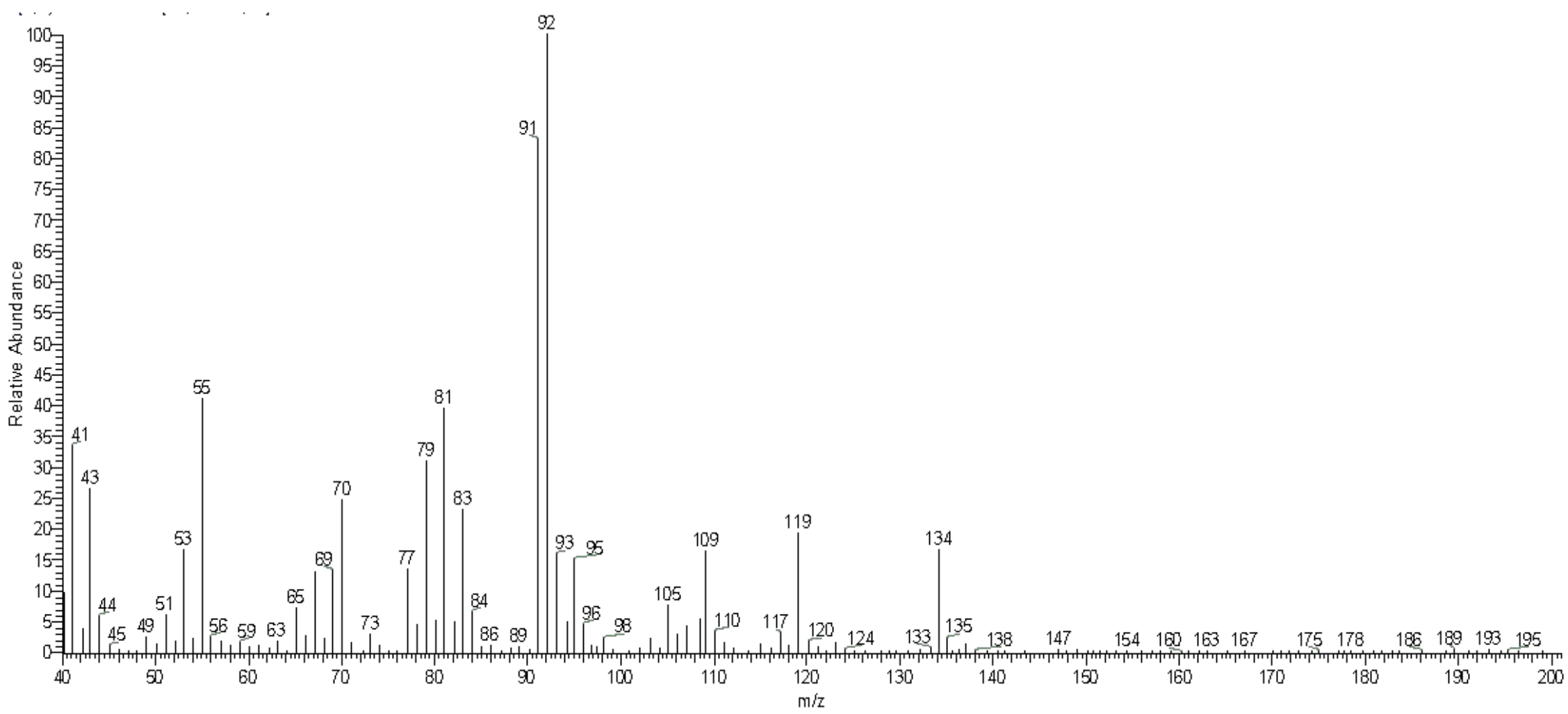

Figure S20. Mass spectrum of Menth-2-en-1-ol <trans-ρ-> (t_R 19.01 min).

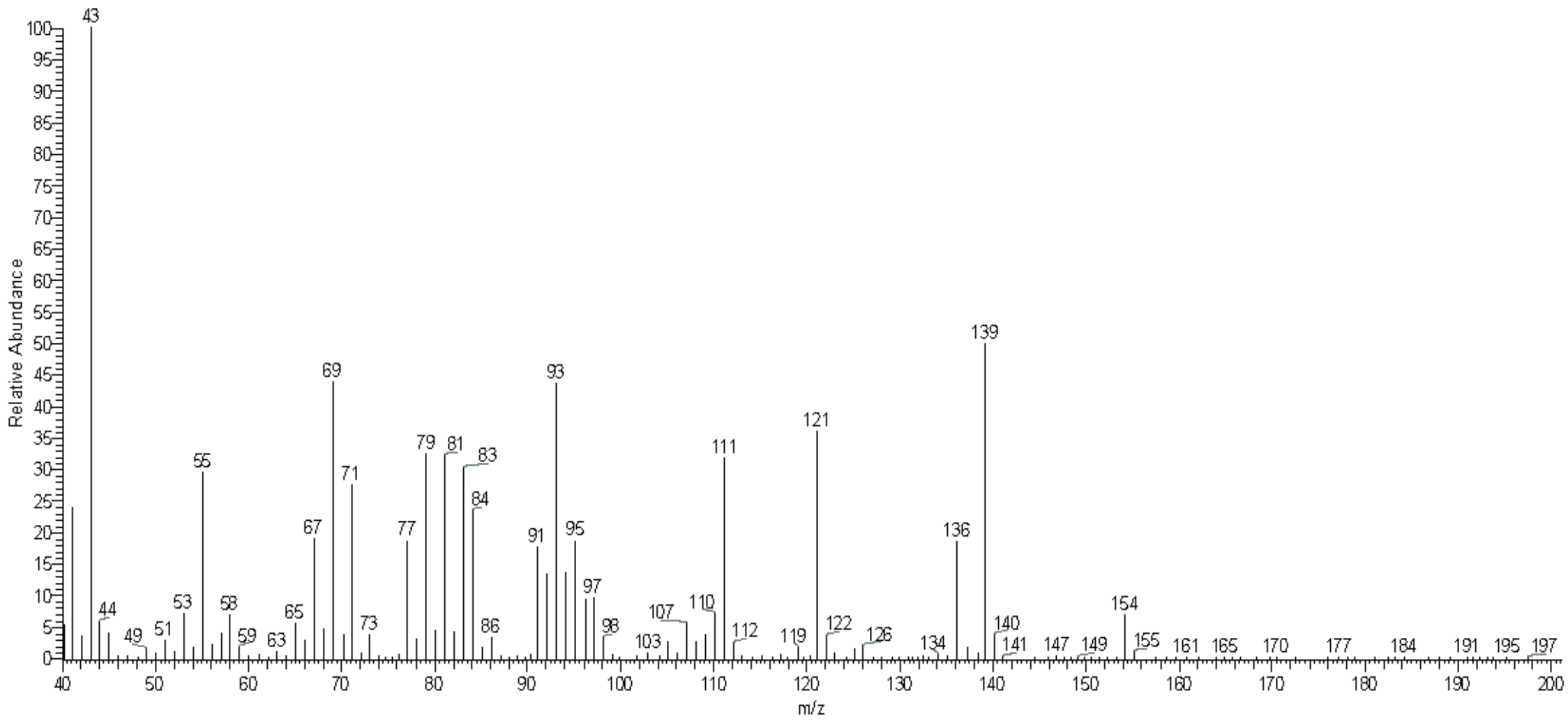

Figure S21. Mass spectrum of Sabina ketone (t_R 19.59 min).

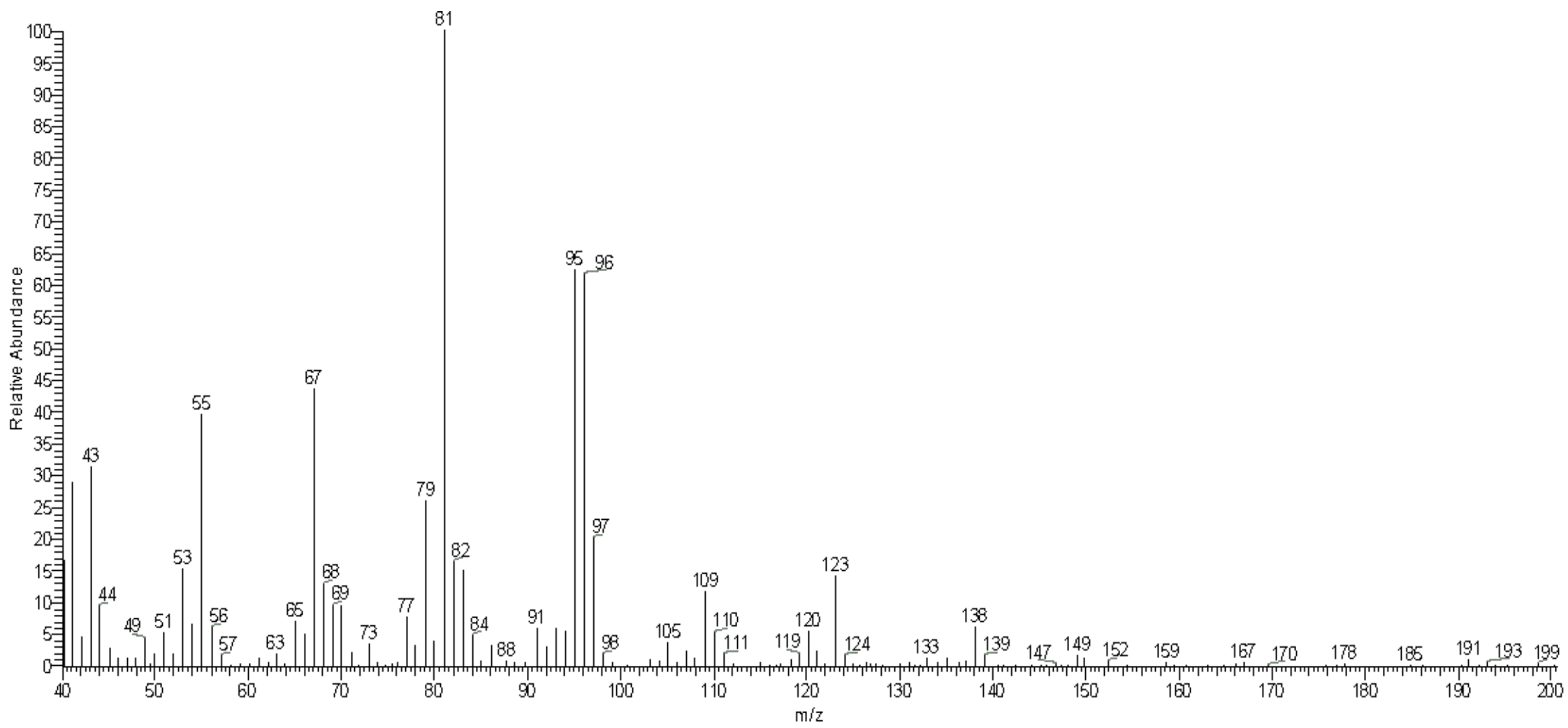

Figure S22. Mass spectrum of Terpinen-4-ol (t_R 20.48 min).

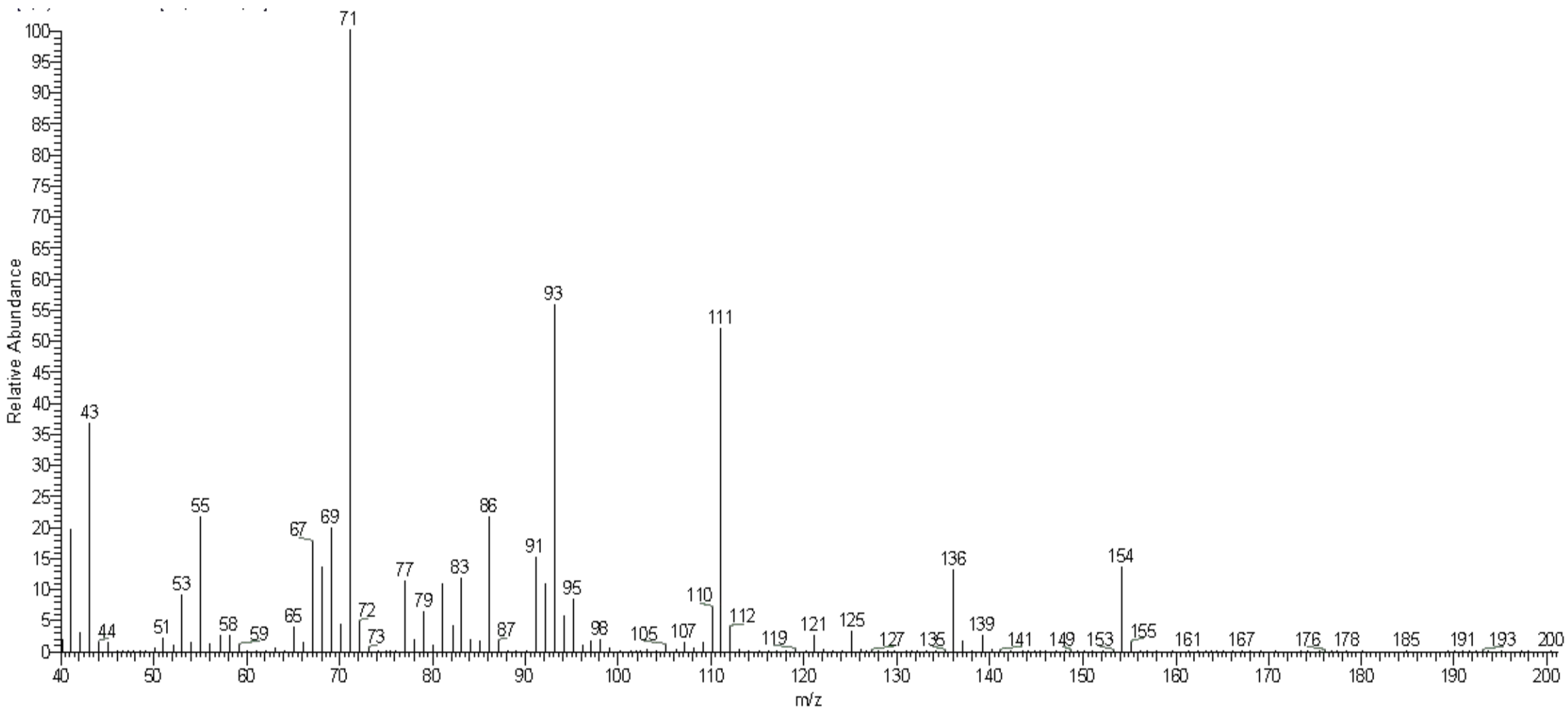

Figure S23. Mass spectrum of Cryptone (t_R 20.68 min).

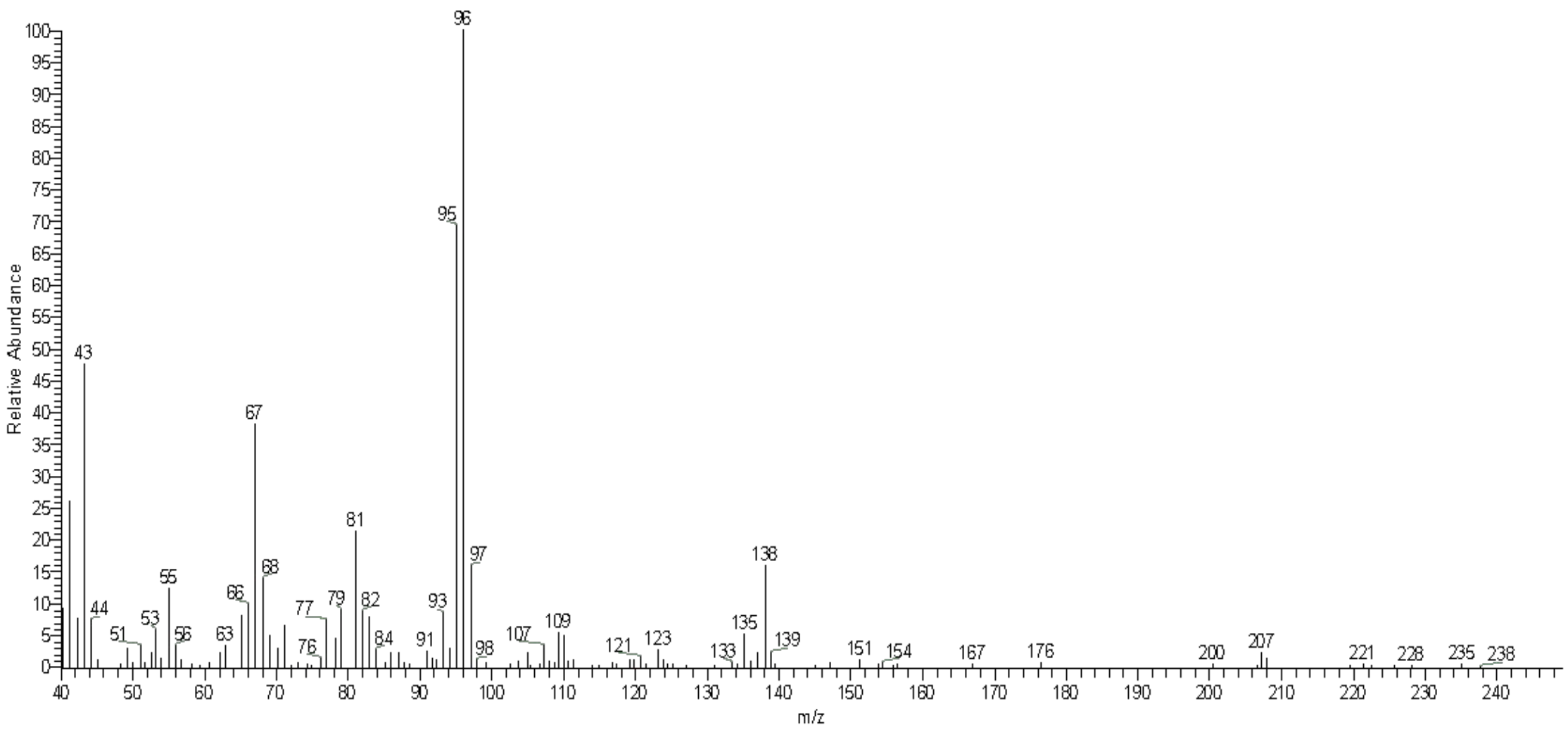

Figure S24. Mass spectrum of Myrtenol (t_R 20.89 min).

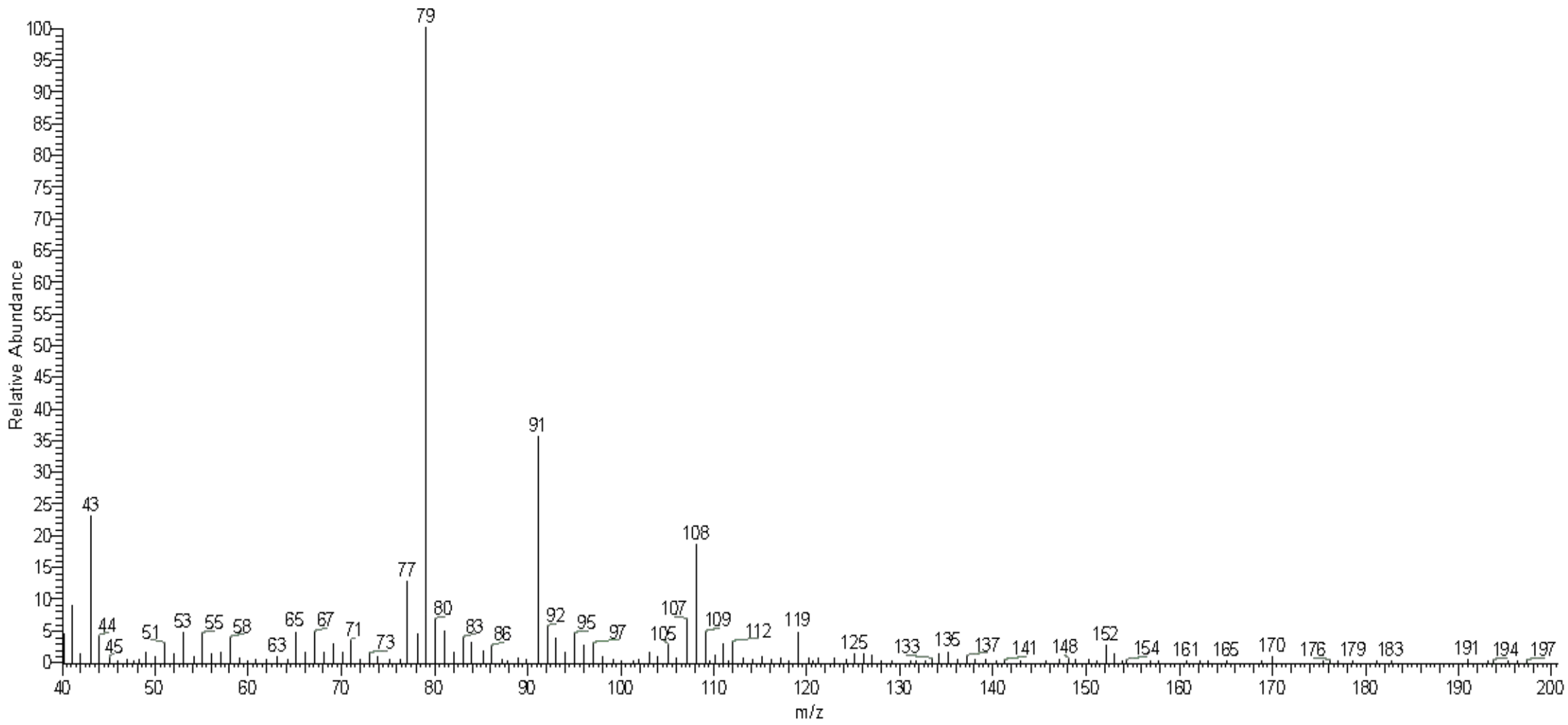

Figure S25. Mass spectrum of α-terpineol (t_R 20.98 min).

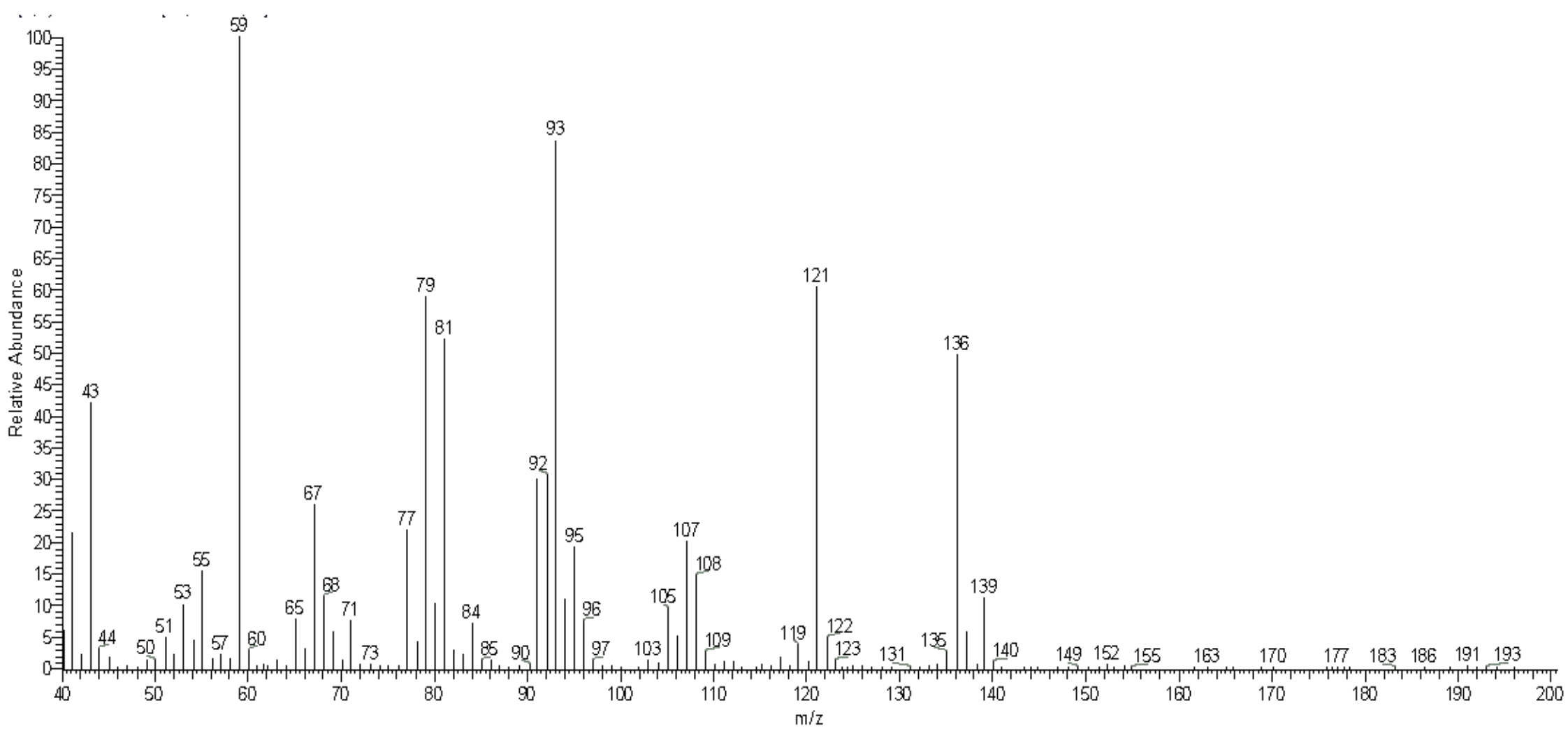

Figure S26. Mass spectrum of trans-Piperitol (t_R 21.49 min).

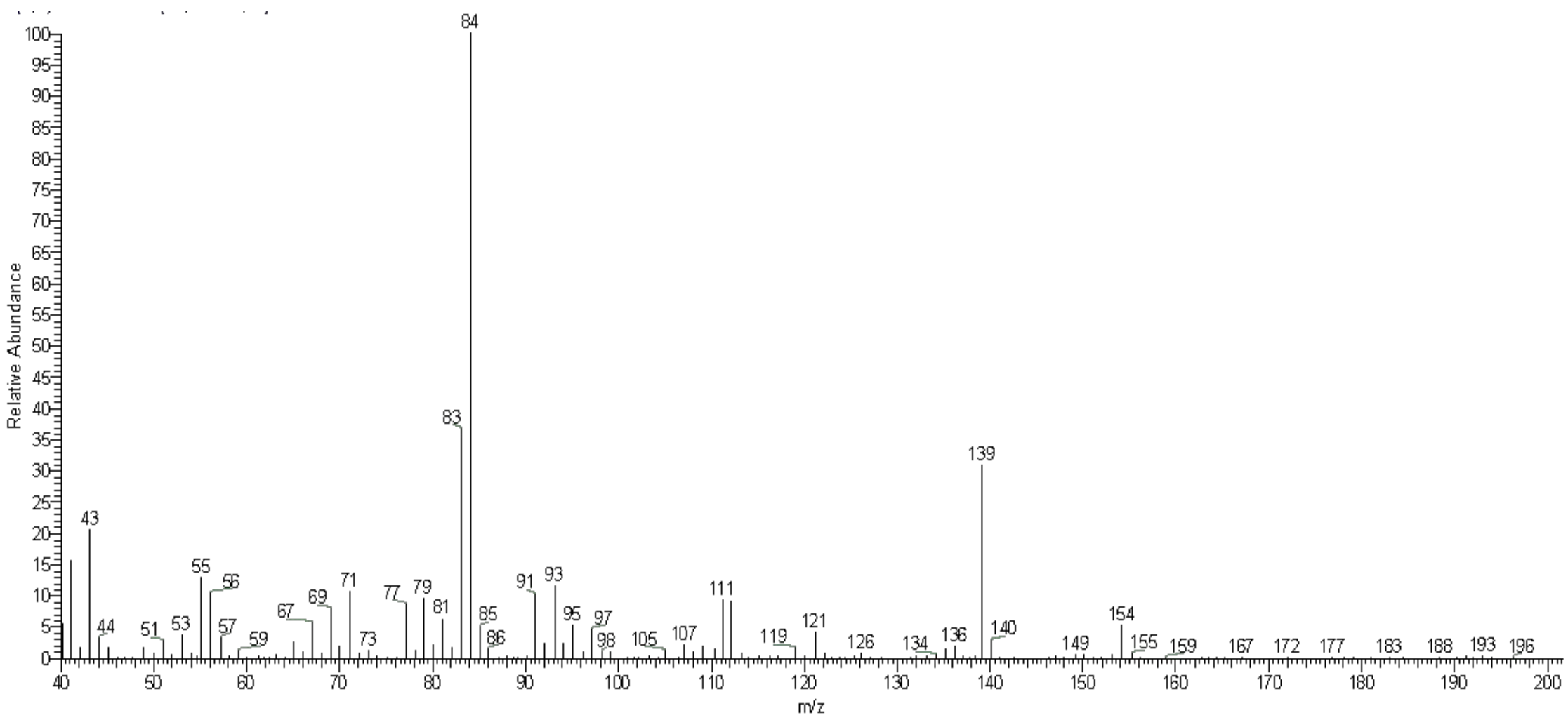

Figure S27. Mass spectrum of trans- Ascaridol glycol (t_R 23.79 min).

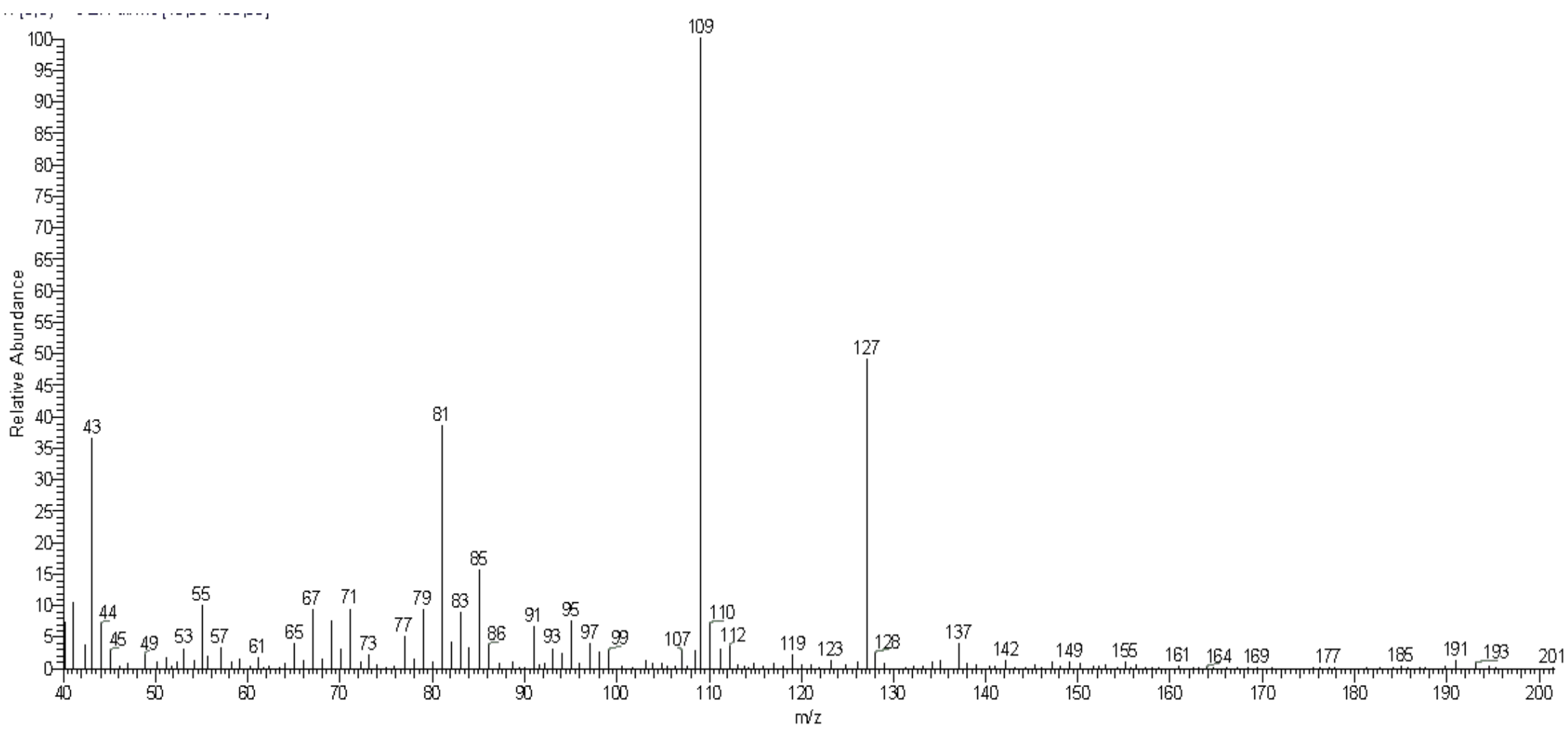

Figure S28. Mass spectrum of α-copaene (t_R 27.17 min).

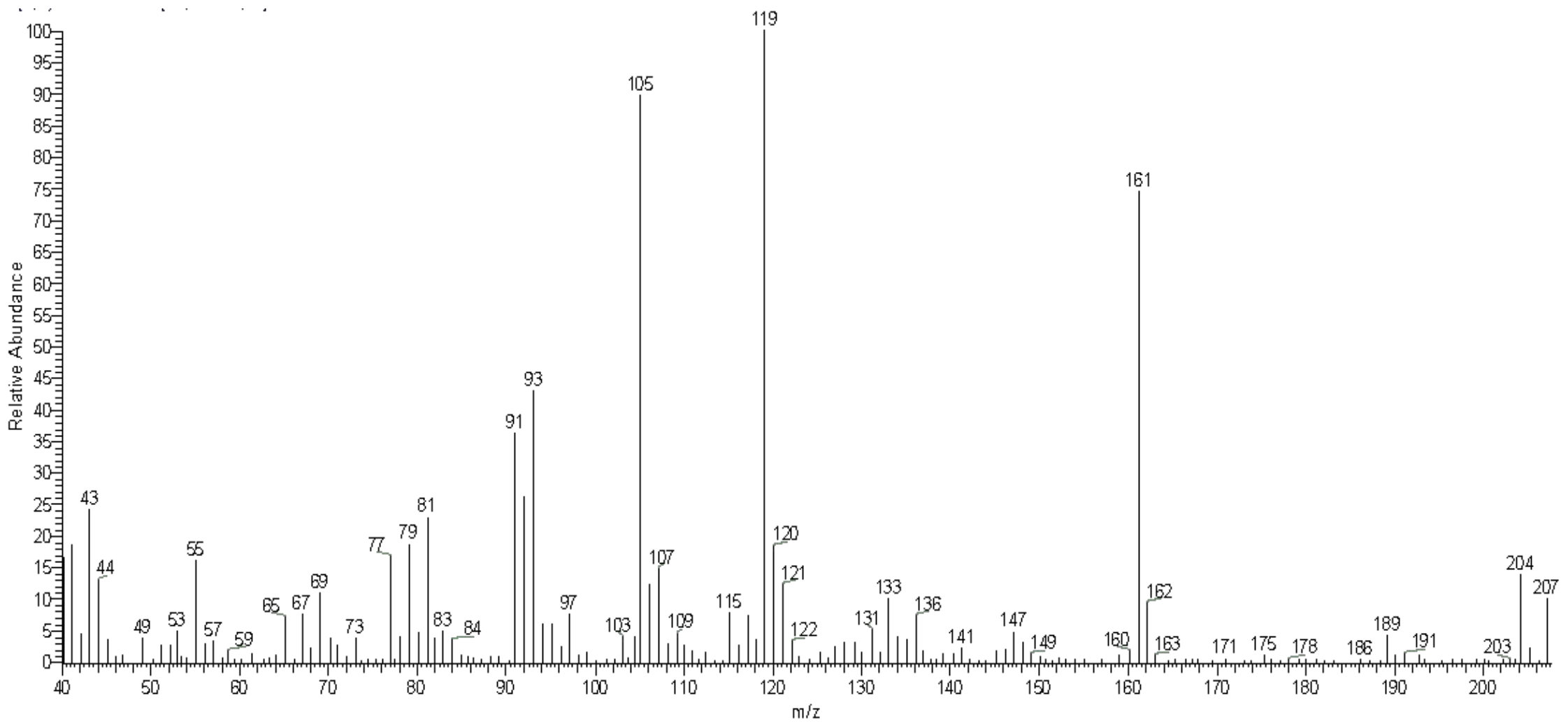

Figure S29. Mass spectrum of (*E*)-Caryophyllene (t_R 28.58 min).

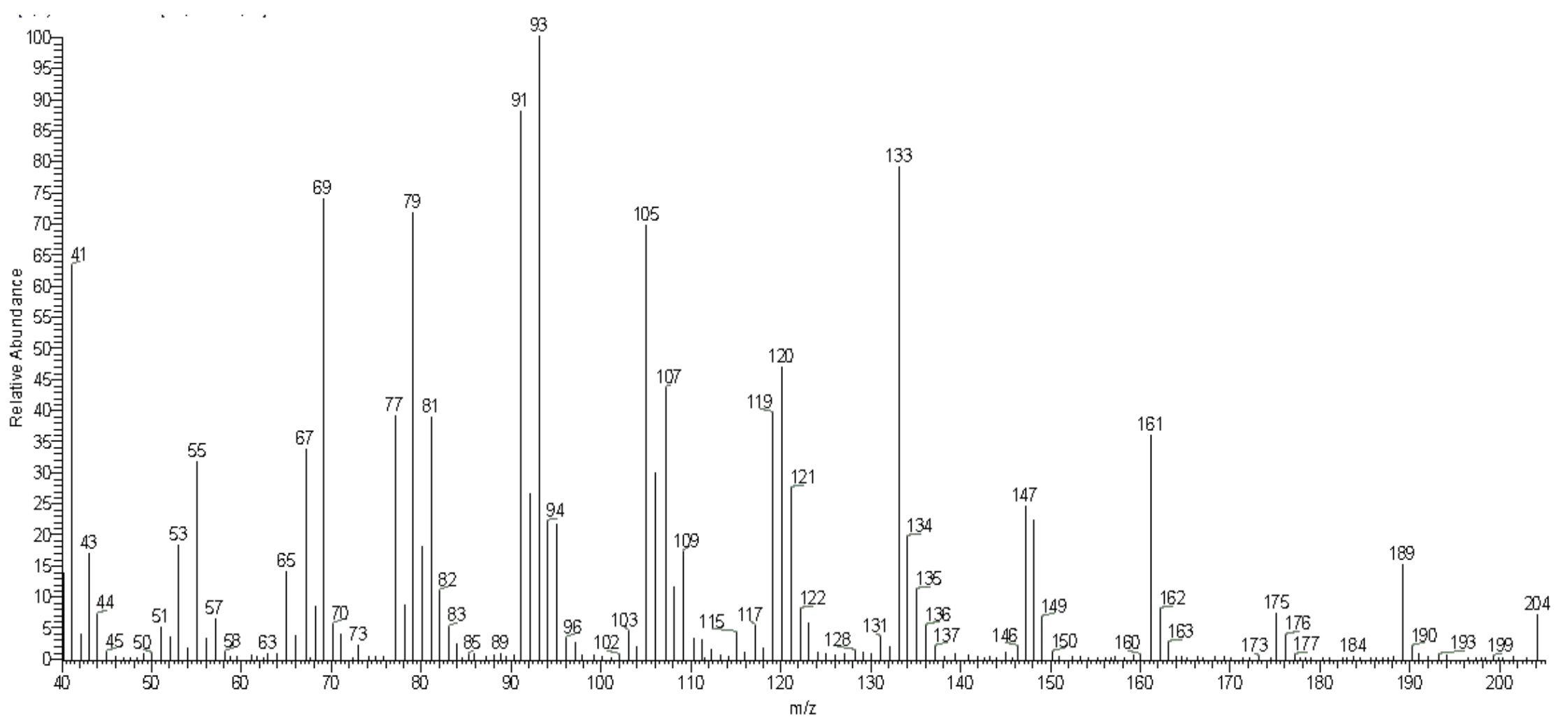

Figure S30. Mass spectrum of *γ*-elemene (t_R 28.90 min).

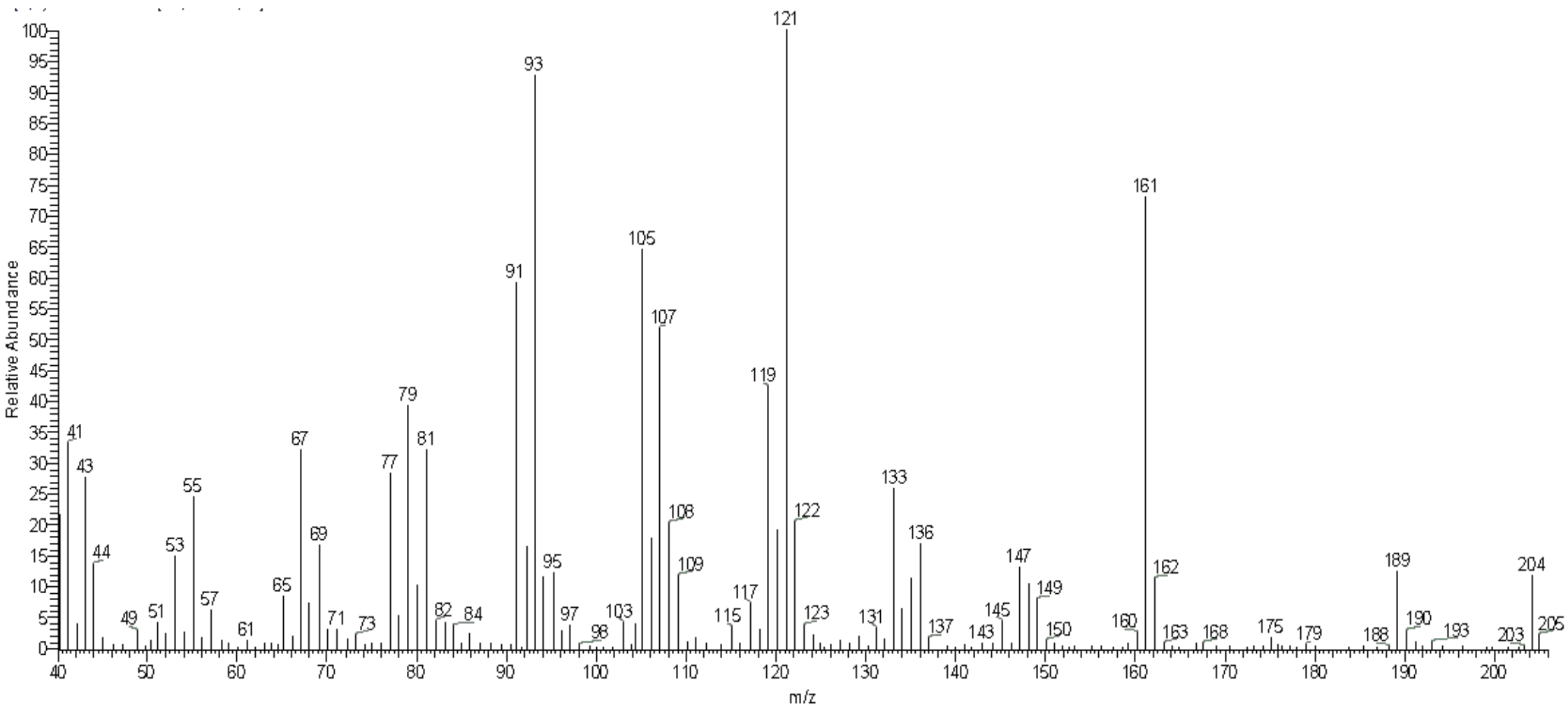

Figure S31. Mass spectrum of α-Humulene (t_R 29.72 min).

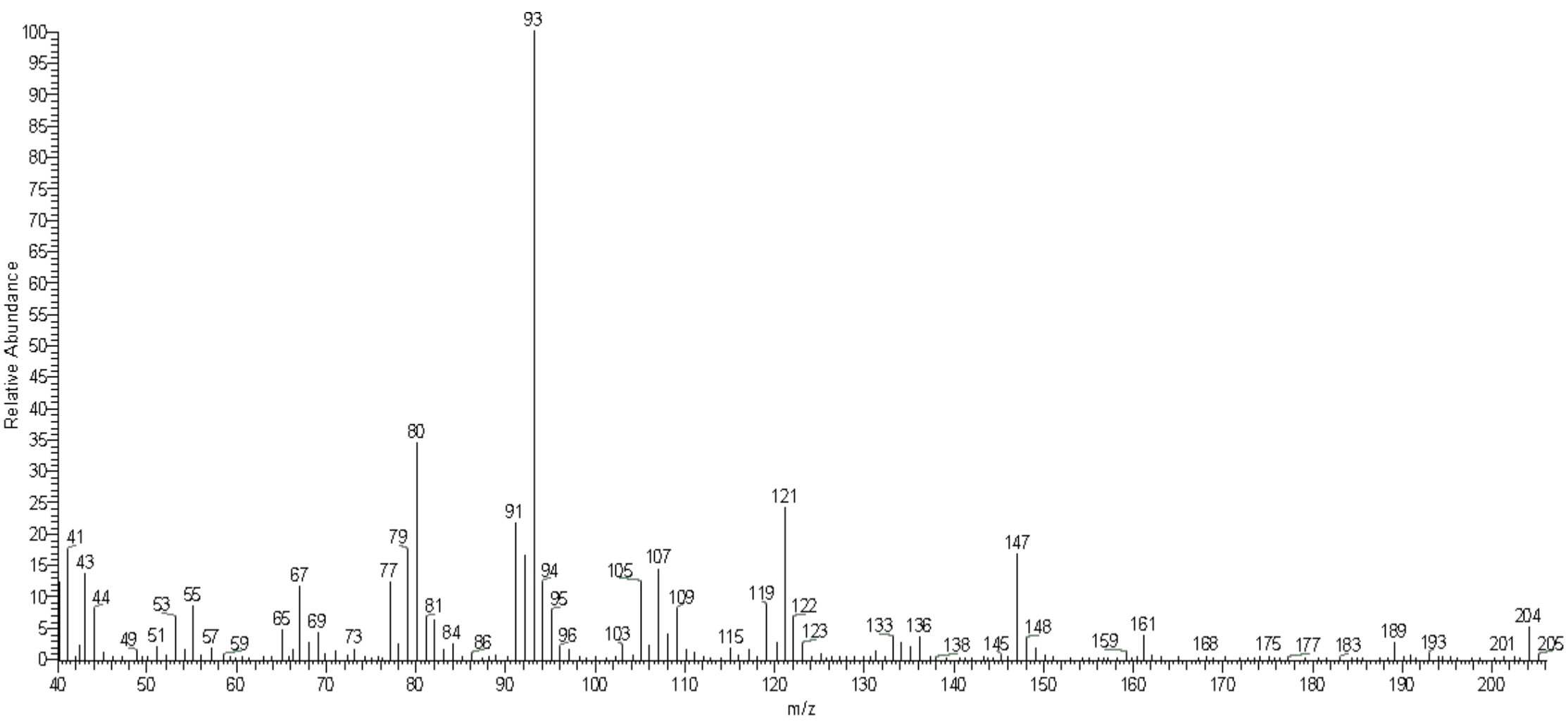

Figure S32. Mass spectrum of Germacrene D (t_R 30.51 min).

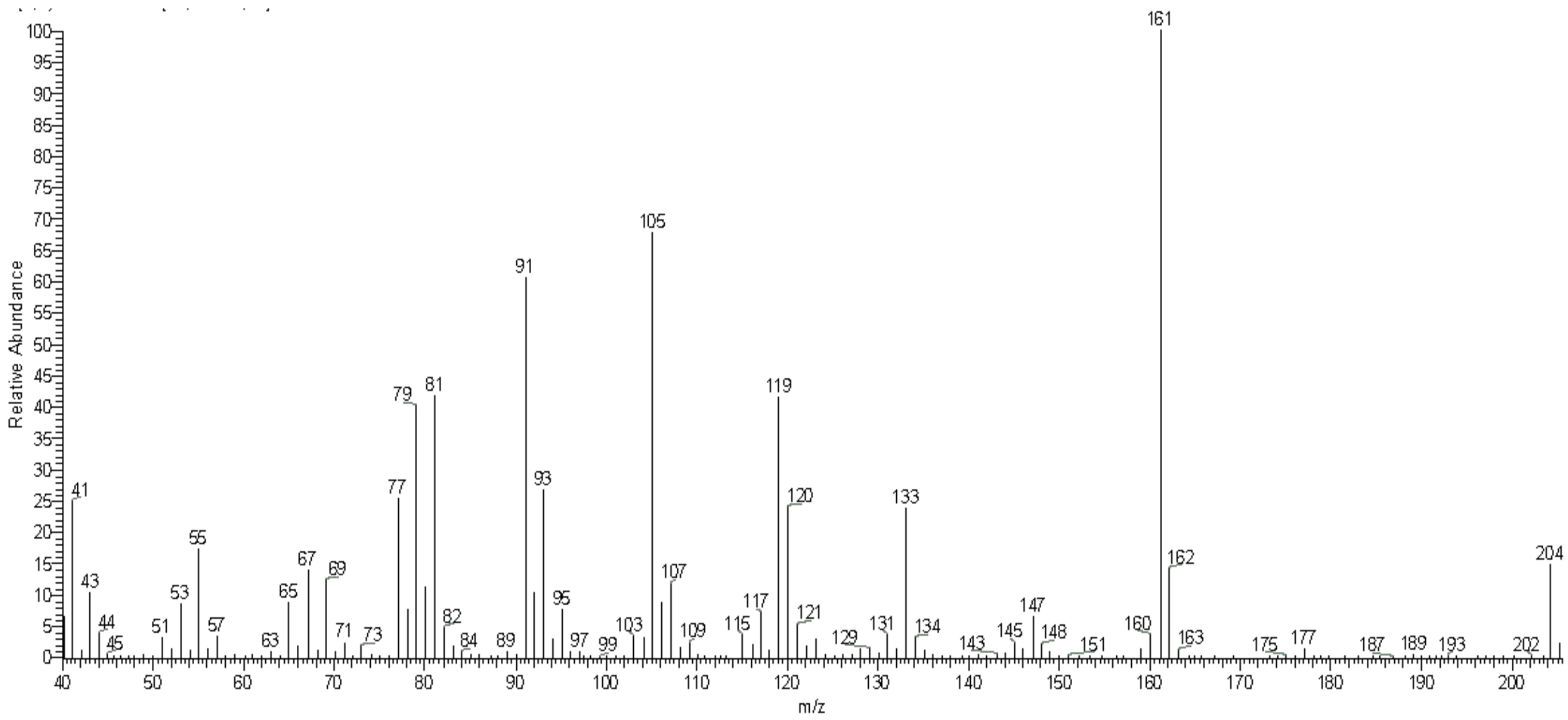

Figure S33. Mass spectrum of Bicyclogermacrene (t_R 30.97 min).

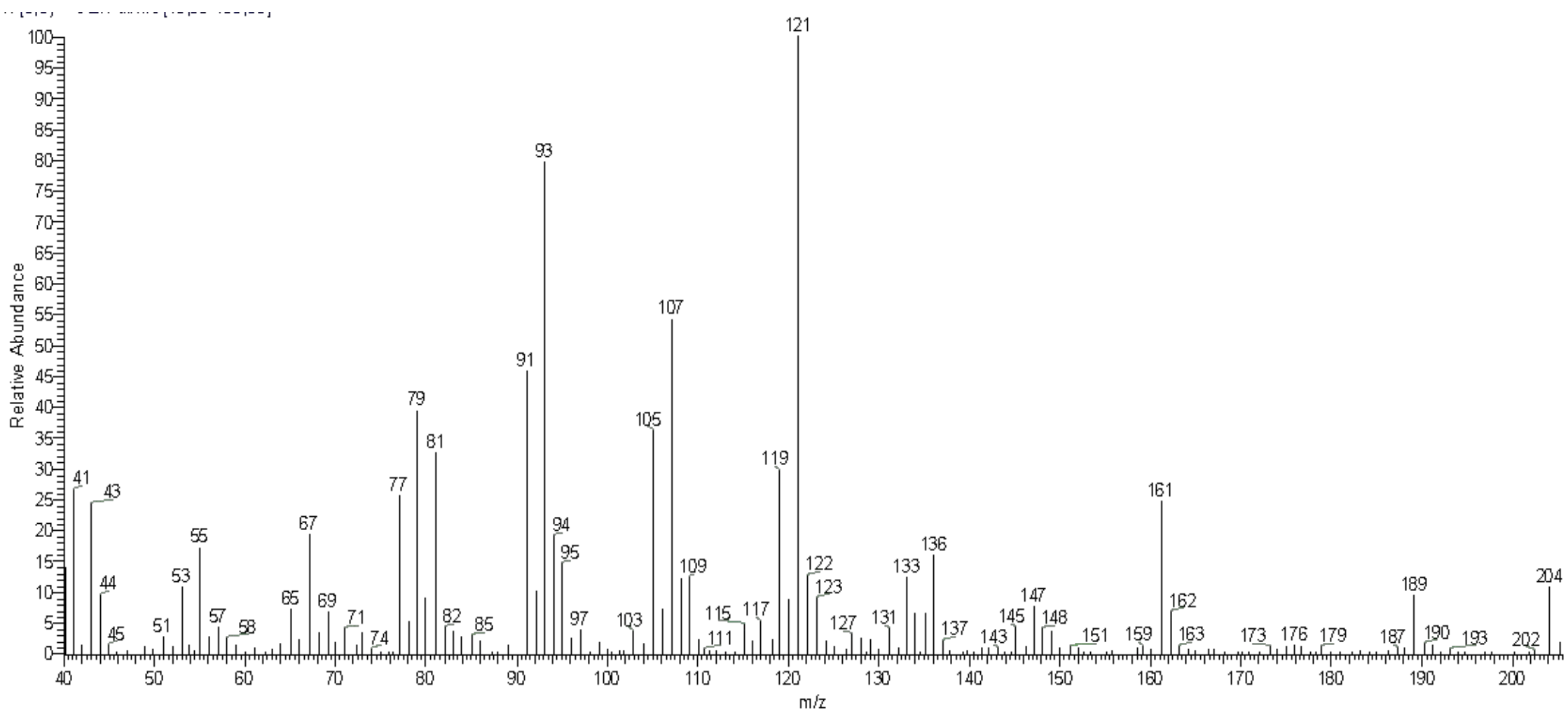

Figure S34. Mass spectrum of β-bisabolene (t_R 31.35 min).

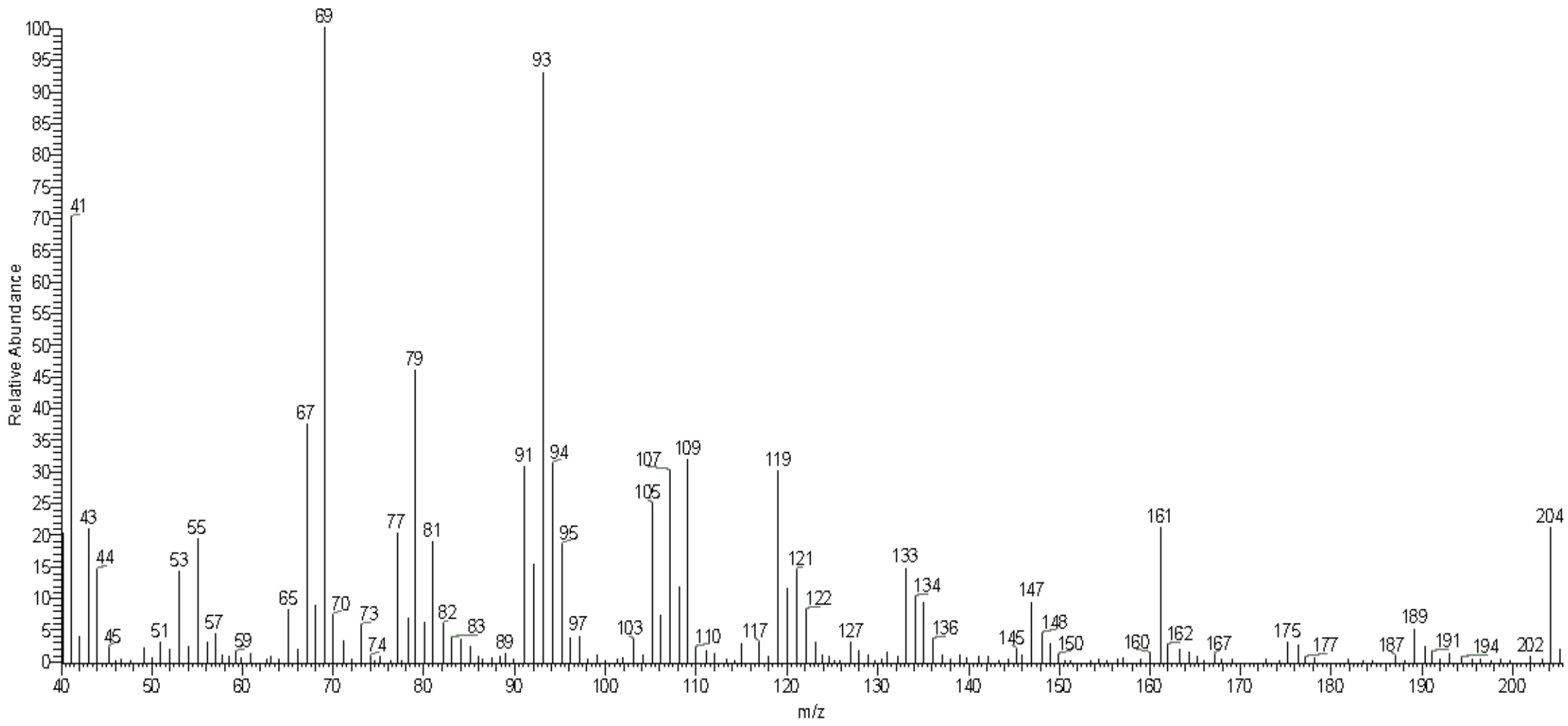

Figure S35. Mass spectrum of δ-amorphene (t_R 31.65 min).

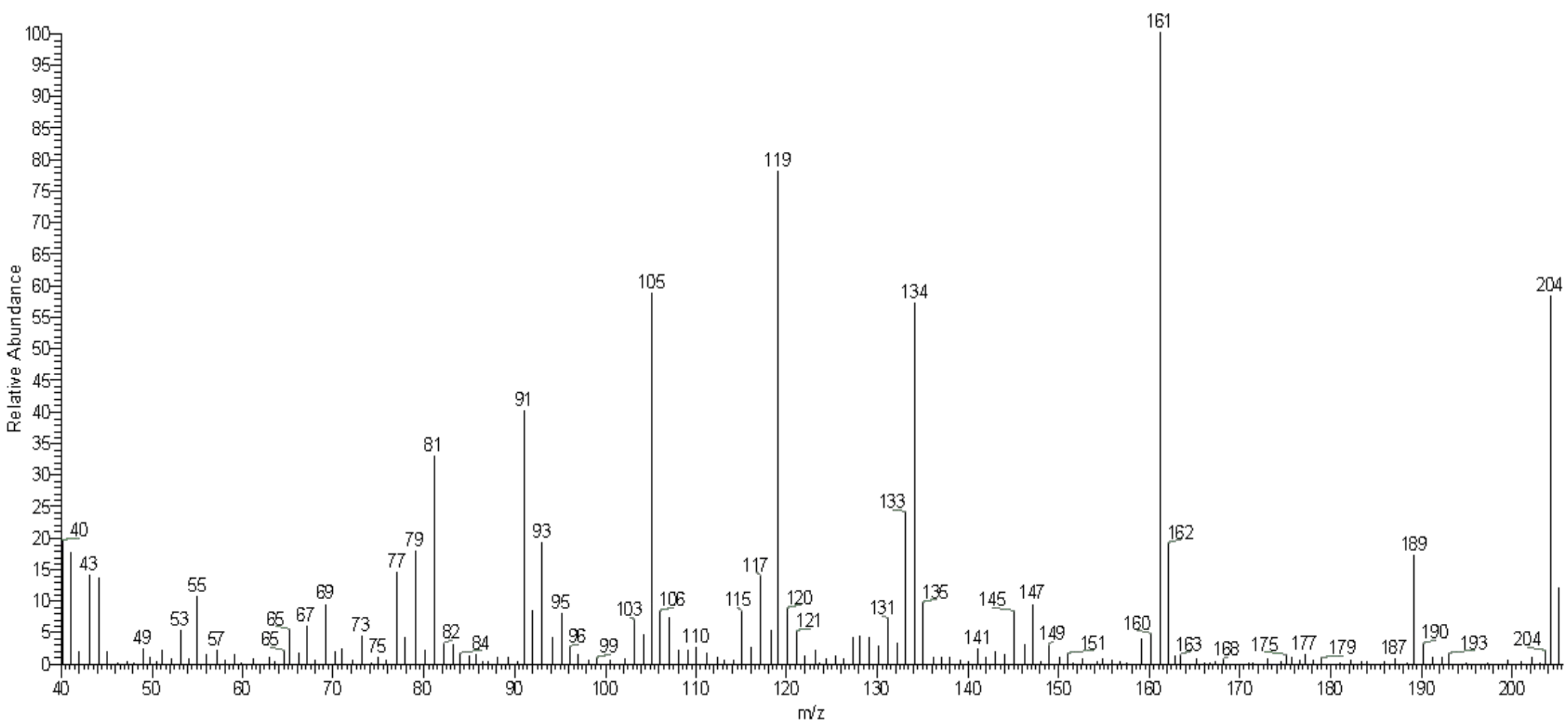

Figure S36. Mass spectrum of Spathulenol (t_R 33.43 min).

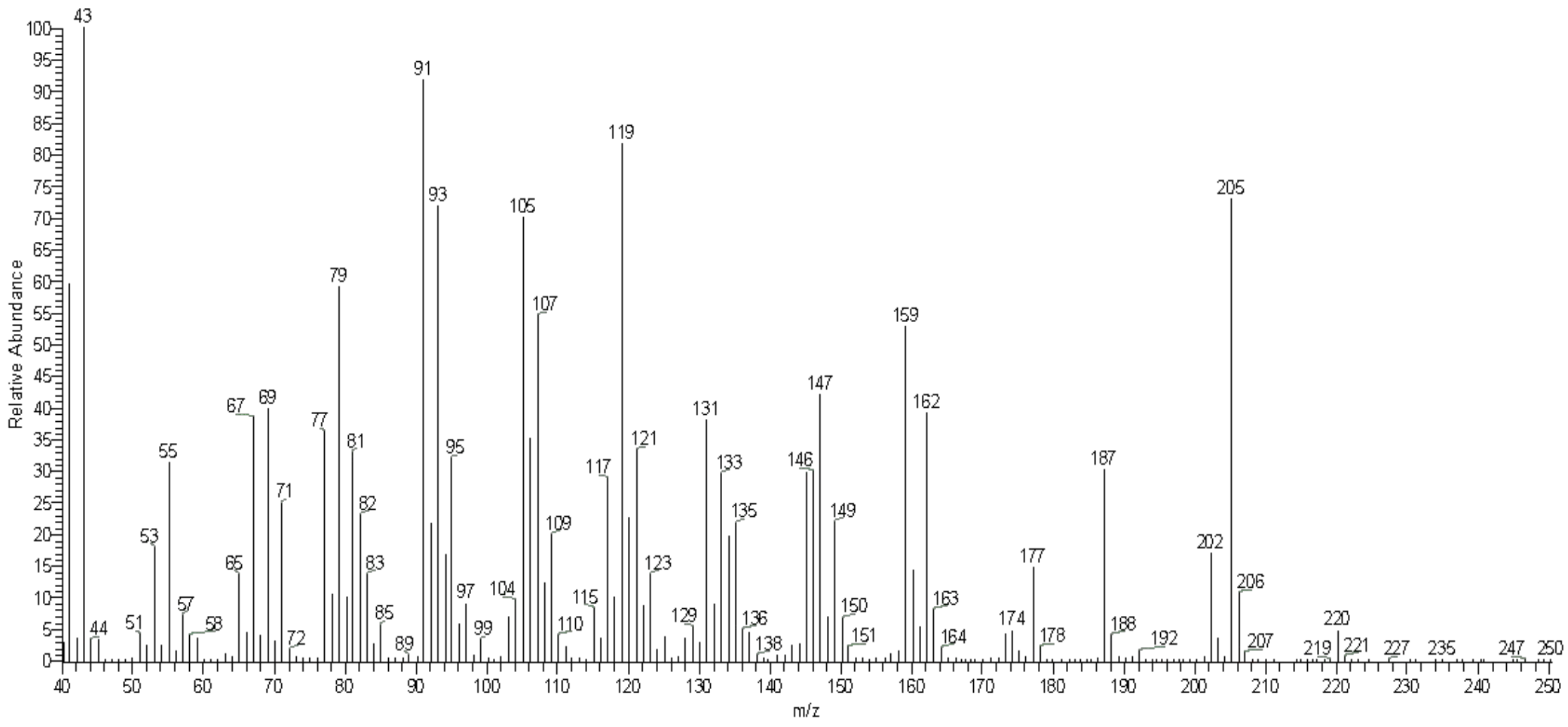

Figure S37. Mass spectrum of caryophyllene oxide (t_R 33.57 min).

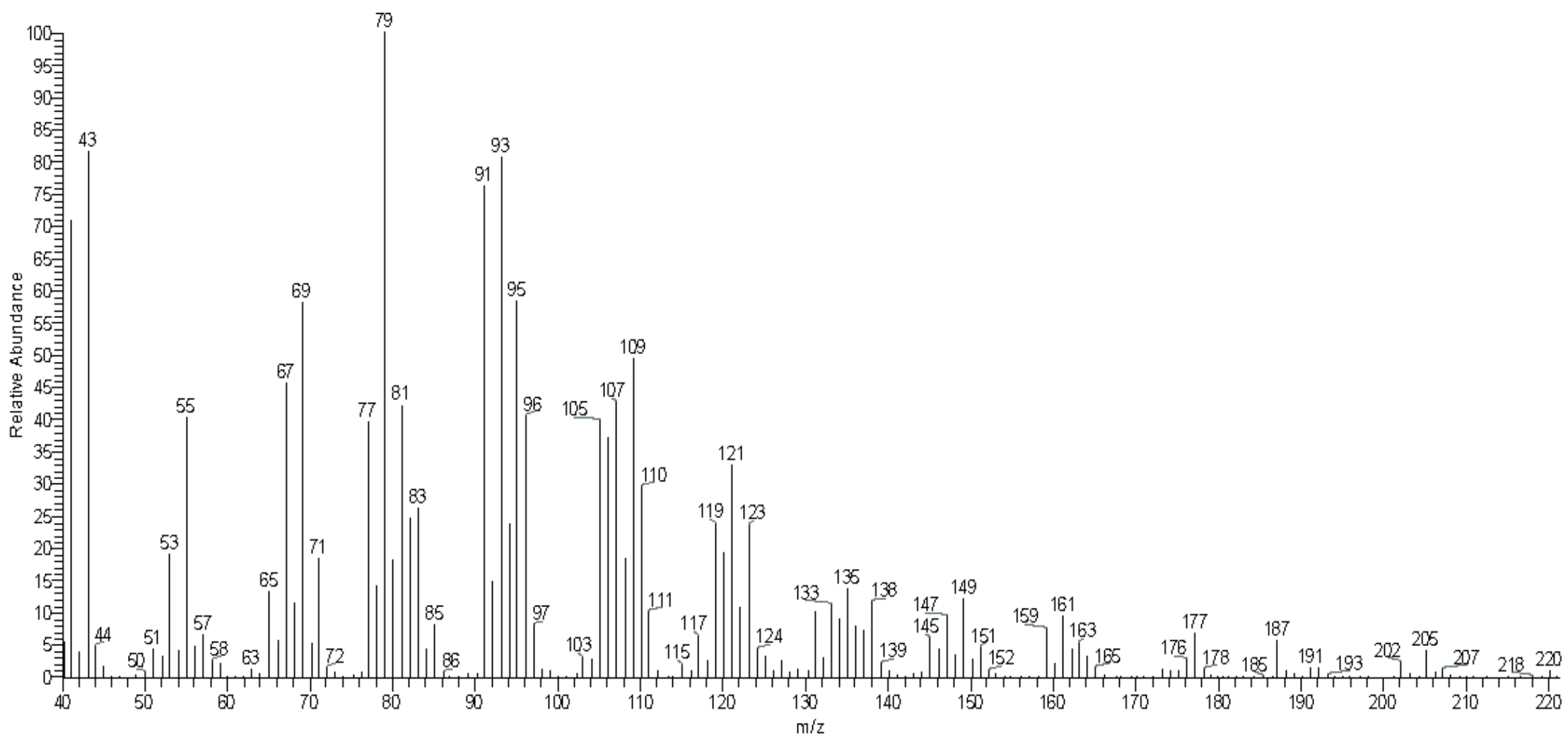

Figure S38. Mass spectrum of Humulene epoxide II (t_R 34.35 min).

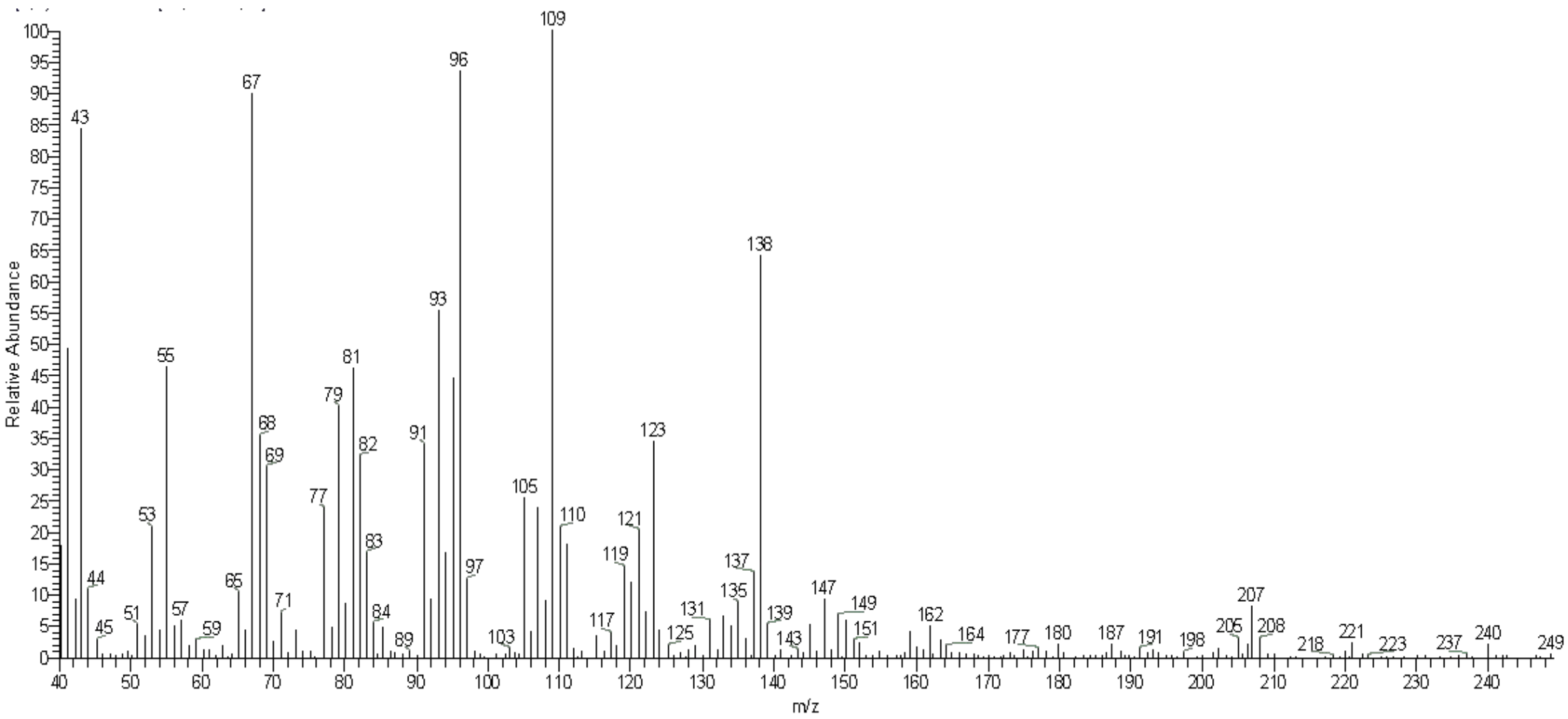

Figure S39. Mass spectrum of Eudesma-4(15),7-dien-1β-ol (impure) (t_R 36.64 min).

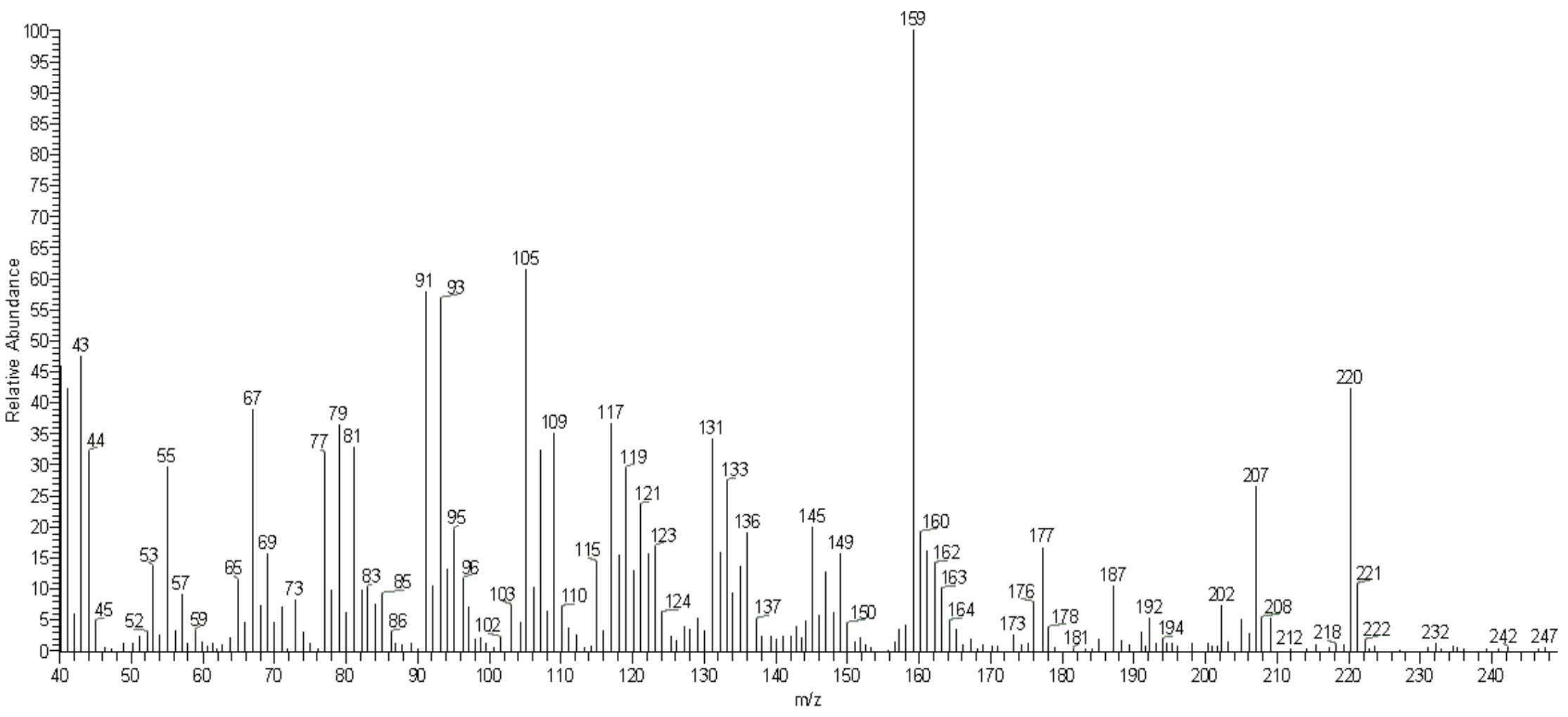

Table S1. Chemical composition of the essential oil of *Duguetia stelechantha* (EODs) determined by GC-MS and GC-FID.

No.	Compound	RI[a]	RI[b]	tR (min)	Percentage (%)	Identification

1	α-Thujene	922	924	10.43	2.25	MS, LRI
2	α-Pinene	929	932	10.69	2.27	MS, LRI
3	Sabinene	971	969	12.38	41.44	MS, LRI, NMR
4	β-Pinene	974	974	12.50	2.30	MS, LRI
5	Myrcene	987	988	13.05	0.31	MS, LRI
6	α-Phellandrene	1003	1002	13.67	0.08	MS, LRI
7	α-Terpinene	1013	1014	14.09	1.63	MS, LRI
8	*p*-Cymene	1022	1020	14.41	3.70	MS, LRI, NMR
9	Limonene	1026	1024	14.59	0.83	MS, LRI
10	β-Phellandrene	1027	1025	14.63	0.51	MS, LRI
11	1,8-Cineole	1029	1026	14.70	1.44	MS, LRI
12	γ-Terpinene	1055	1054	15.75	3.08	MS, LRI
13	Sabinene hydrate (*cis*)	1068	1065	16.23	0.38	MS, LRI
14	Terpinolene	1082	1086	16.80	0.73	MS, LRI
15	Sabinene hydrate (*trans*)	1098	1098	17.43	0.30	MS, LRI
16	Fenchol (*endo*)	1116	1114	18.11	0.10	MS, LRI
17	Menth-2-en-1-ol (*cis*-ρ)	1122	1118	18.32	0.70	MS, LRI
18	Sabinol (*trans*)	1137	1137	18.91	0.23	MS, LRI
19	Menth-2-en-1-ol (*trans*-ρ)	1140	1136	19.01	0.47	MS, LRI
20	Sabina ketone	1156	1154	19.59	0.16	MS, LRI

21	Terpinen-4-ol	1179	1174	20.48	17.61	MS, LRI, NMR
22	Cryptone	1185	1183	20.68	0.57	MS, LRI
23	Myrtenol	1190	1194	20.89	0.19	MS, LRI
24	α-Terpineol	1193	1186	20.98	0.97	MS, LRI
25	*trans*-Piperitol	1207	1207	21.49	0.25	MS, LRI
26	*trans*-Ascaridol glycol	1272	1266	23.79	0.11	MS, LRI
27	α-Copaene	1373	1374	27.17	0.11	MS, LRI
28	(*E*)-Caryophyllene	1416	1417	28.58	0.82	MS, LRI
29	γ-Elemene	1426	1434	28.90	0.22	MS, LRI
30	α-Humulene	1452	1452	29.72	0.11	MS, LRI
31	Germacrene D	1478	1480	30.51	0.45	MS, LRI
32	Bicyclogermacrene	1492	1500	30.97	0.19	MS, LRI
33	β-Bisabolene	1505	1505	31.35	0.11	MS, LRI
34	δ-Amorphene	1515	1511	31.65	0.12	MS, LRI
35	Spathulenol	1575	1577	33.43	9.21	MS, LRI
36	Caryophyllene oxide	1579	1582	33.57	2.90	MS, LRI
37	Humulene epoxide II	1606	1608	34.35	0.26	MS, LRI
38	Eudesma-4(15),7-dien-1β-ol	1688	1687	36.64	0.06	MS, LRI
	**Total monoterpenes**				**83.11**	
	**Total sesquiterpenes**				**14.56**	

Total identified **97.67**

Footnotes:

[a] Experimental retention index calculated using the Van den Dool–Kratz equation.

[b] Literature retention index according to Adams.

RI: retention index; tR: retention time; MS: mass spectral comparison; LRI: linear retention index. Compounds were identified based on mass spectral comparison and consistency between experimental and literature retention indices.

Figure S40. ^{1}H NMR spectrum of EODs (500 MHz, $CDCl_3$).

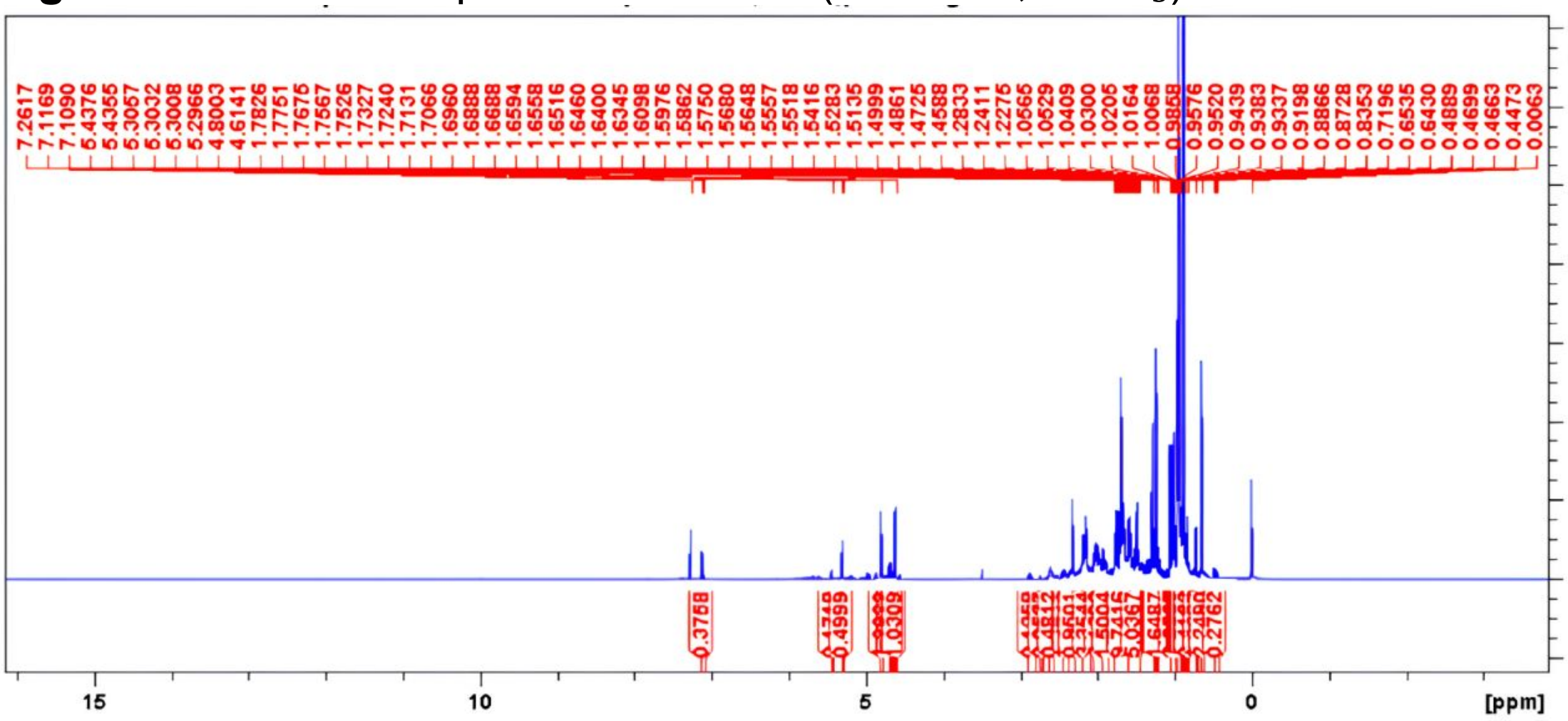

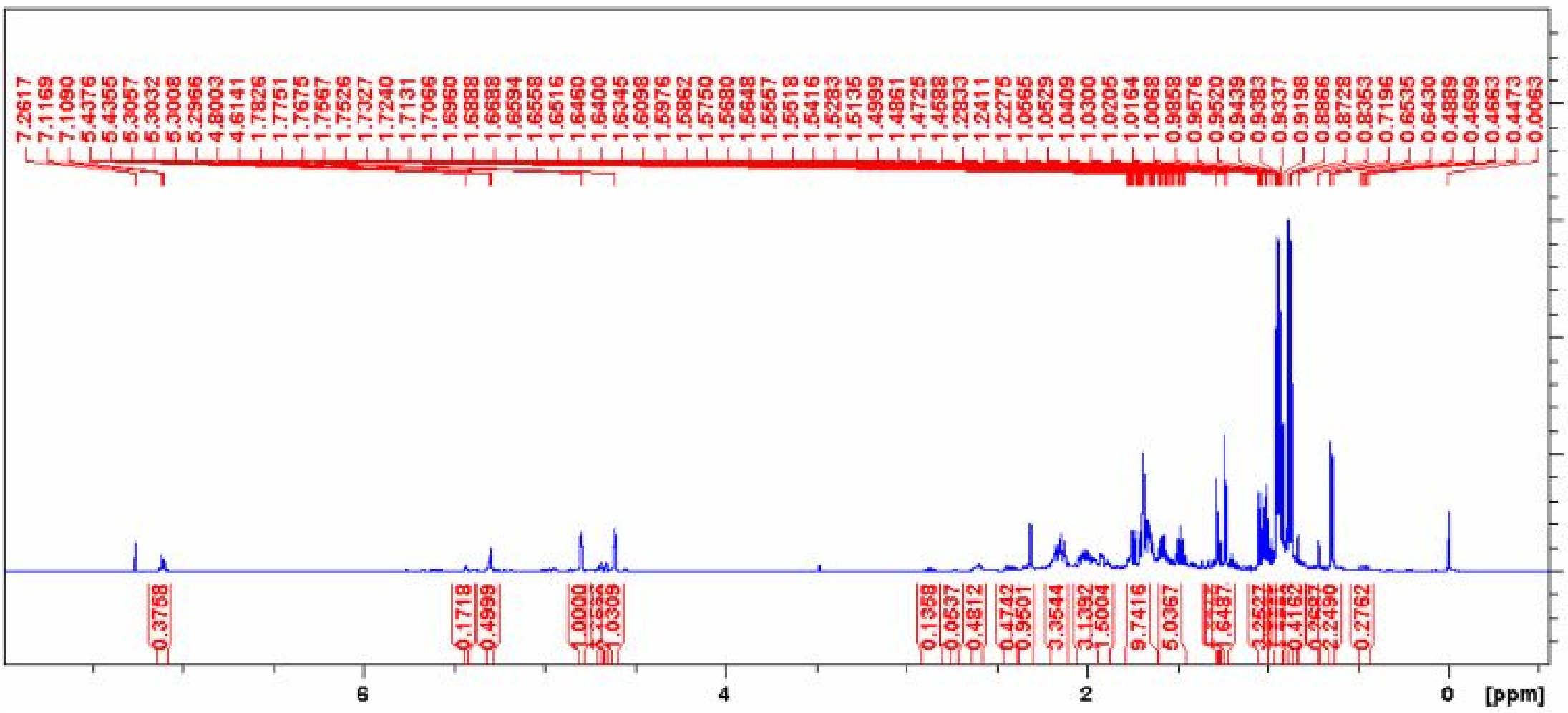
[rel]
20
15
10
5
0
6
4
2
0
[ppm]

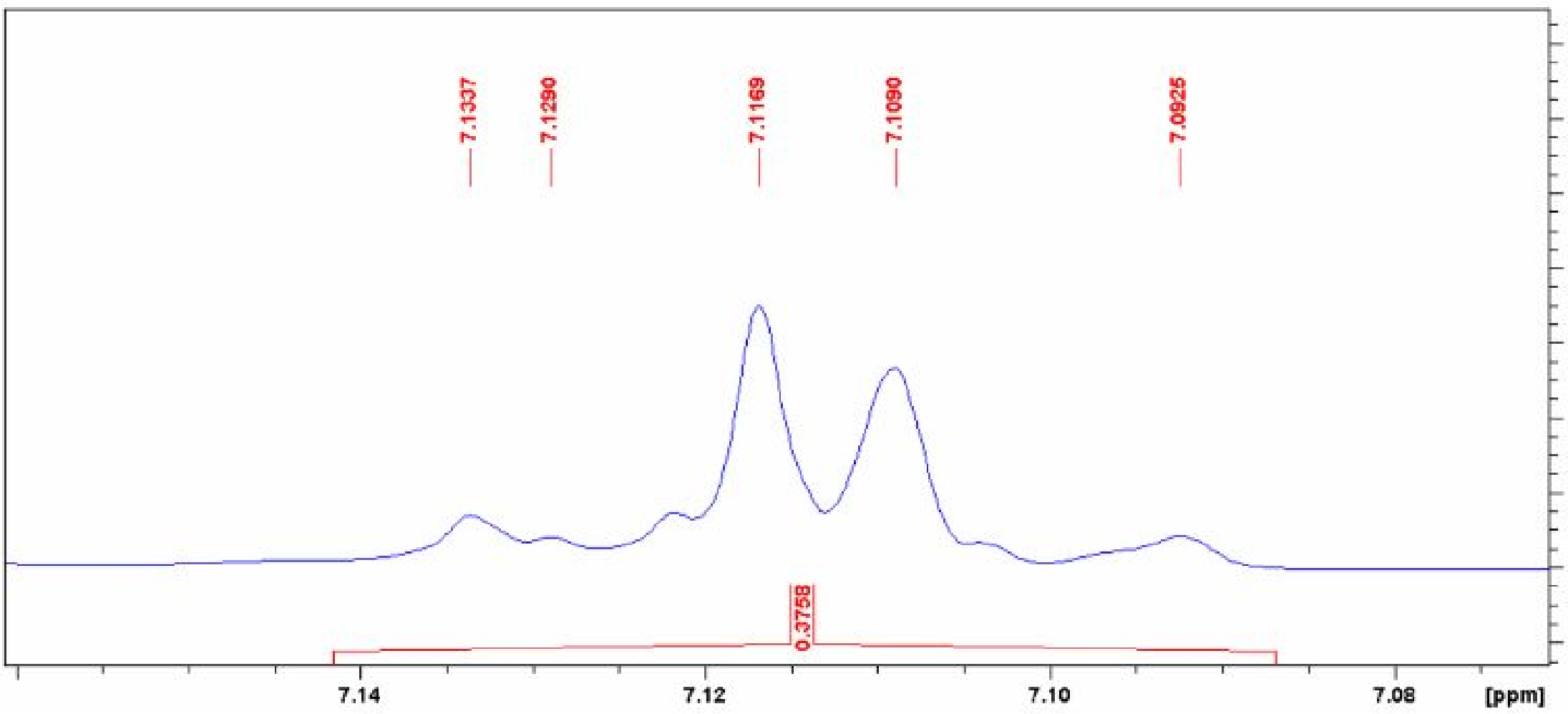
[rel]
7.1337
7.1290
7.1169
7.1090
7.0925
1.2
1.0
0.8
0.6
0.4
0.2
0.0
0.3758
7.14
7.12
7.10
7.08
[ppm]

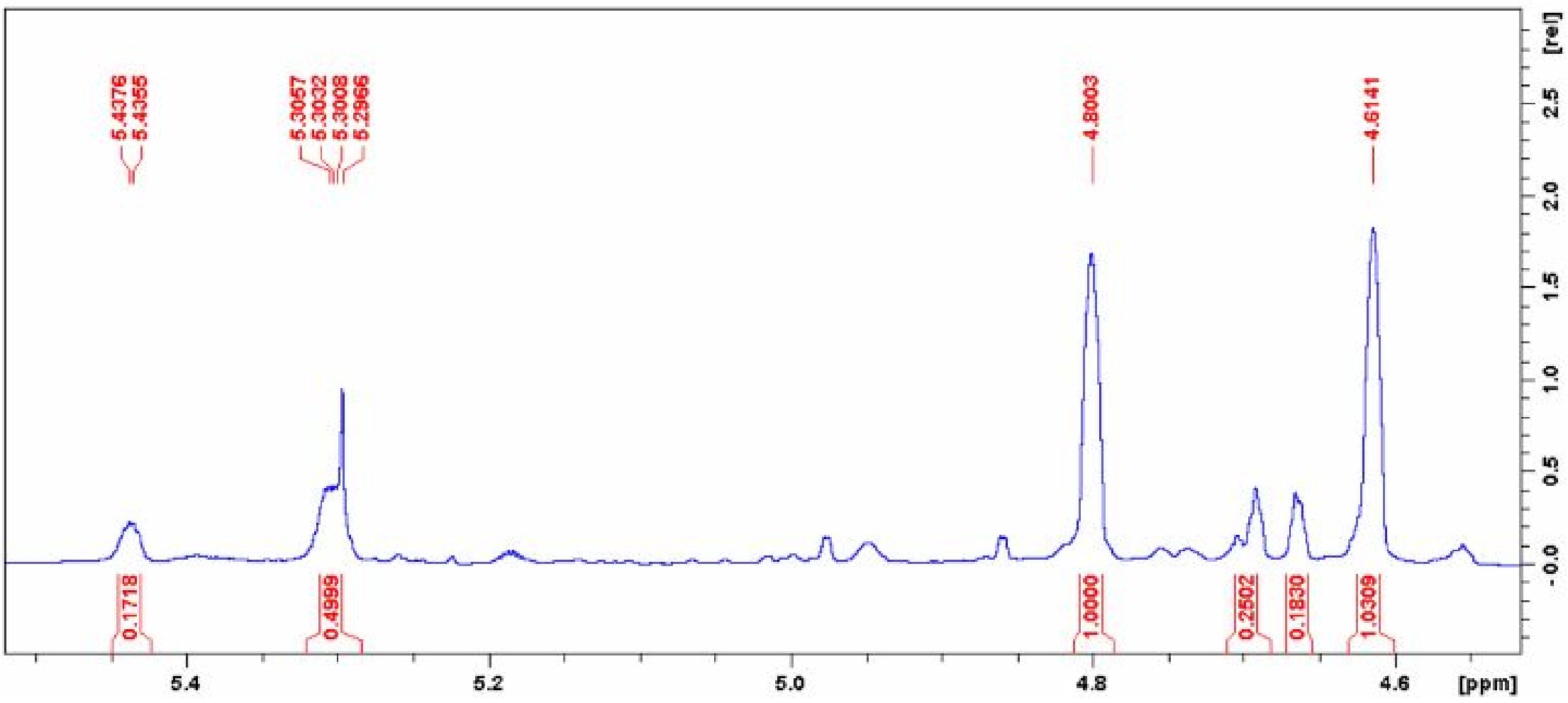
5.4376
5.4355
5.3057
5.3032
5.3008
5.2966
4.8003
4.6141
0.1718
0.4999
1.0000
0.2502
0.1830
1.0309
[rel]
[ppm]

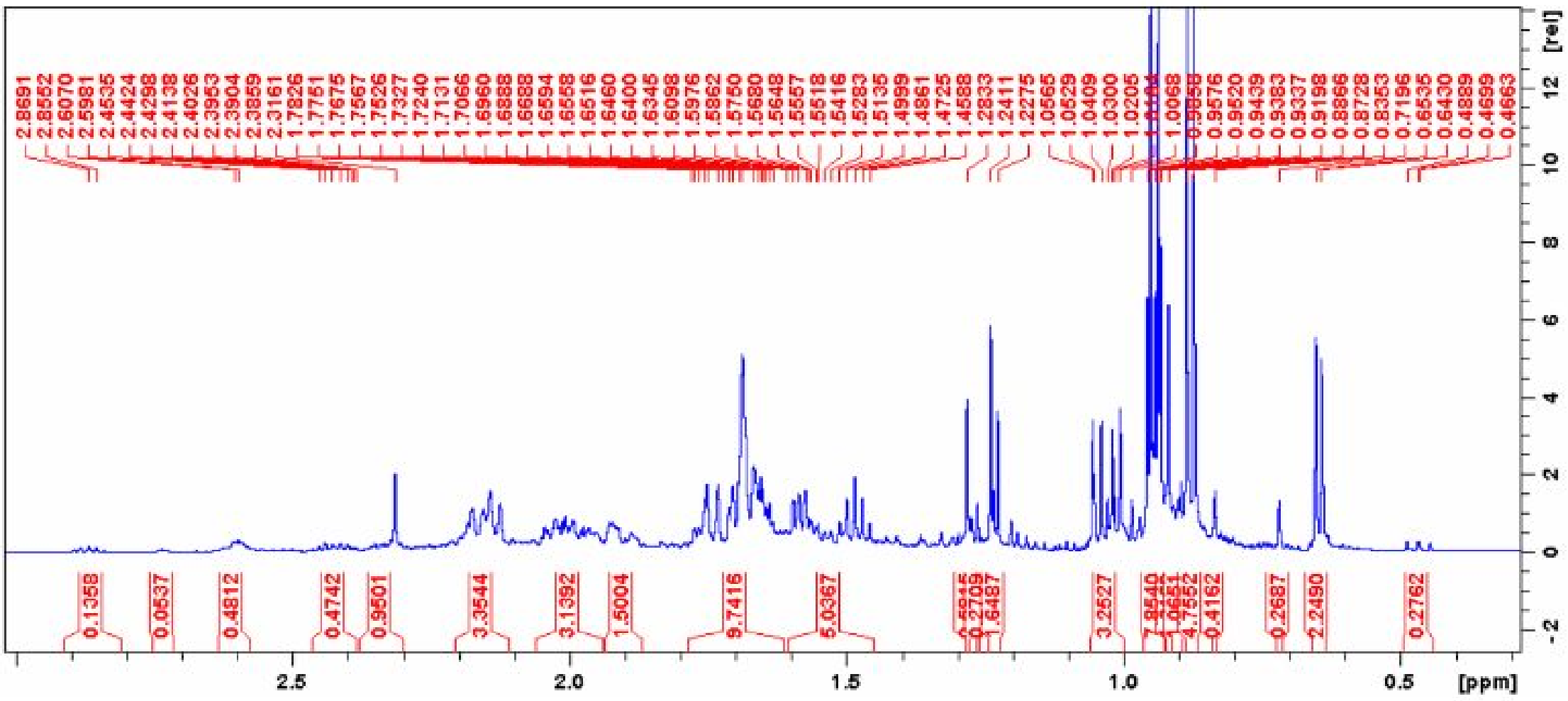
0.1358
0.0537
0.4812
0.4742
0.9501
3.3544
3.1392
1.5004
9.7416
5.0367
1.6487
3.2627
2.2490
0.2762
[rel]
[ppm]

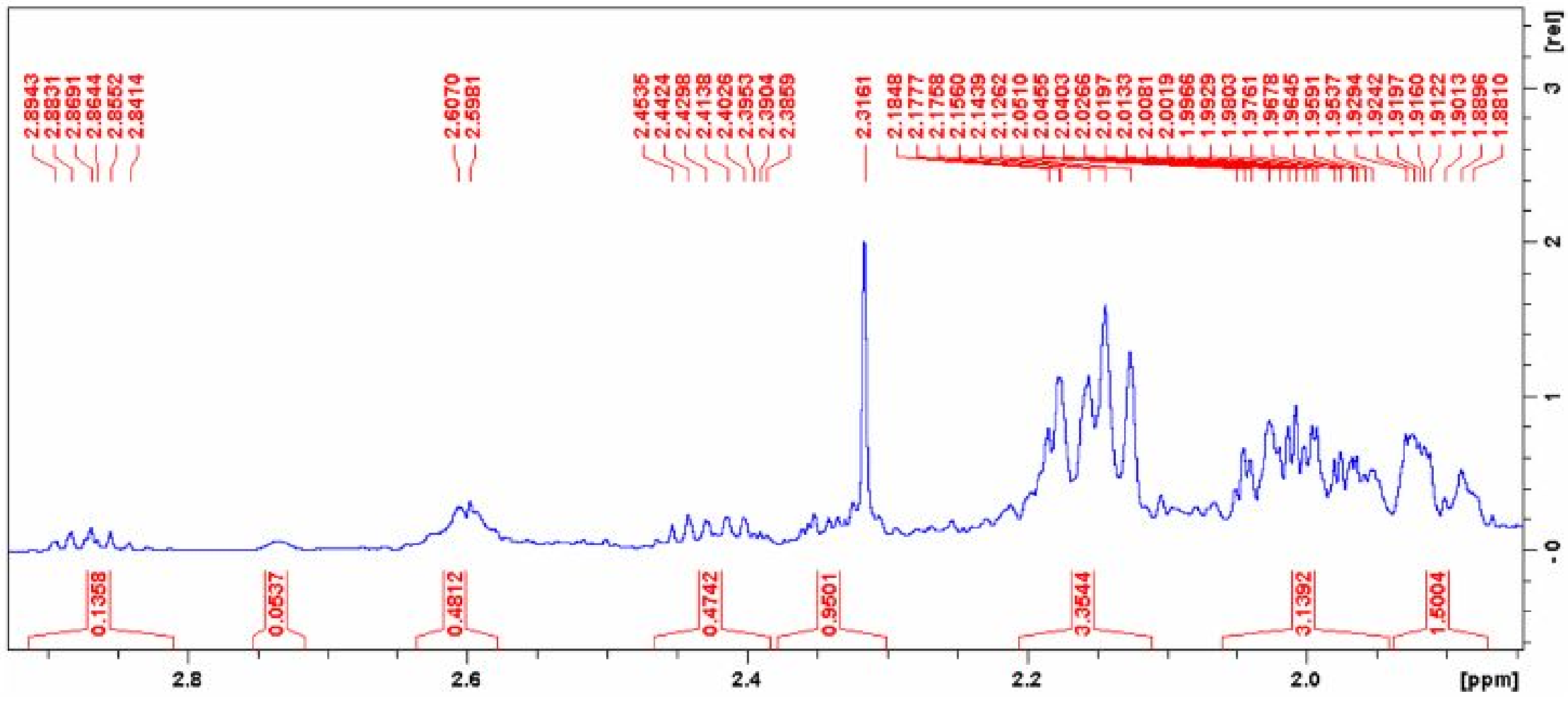
2.8943
2.8831
2.8691
2.8644
2.8552
2.8414
2.6070
2.5981
2.4535
2.4424
2.4298
2.4138
2.4026
2.3953
2.3904
2.3859
2.3161
0.1358
0.0537
0.4812
0.4742
0.9501
3.3544
3.1392
1.5004
[rel]
[ppm]

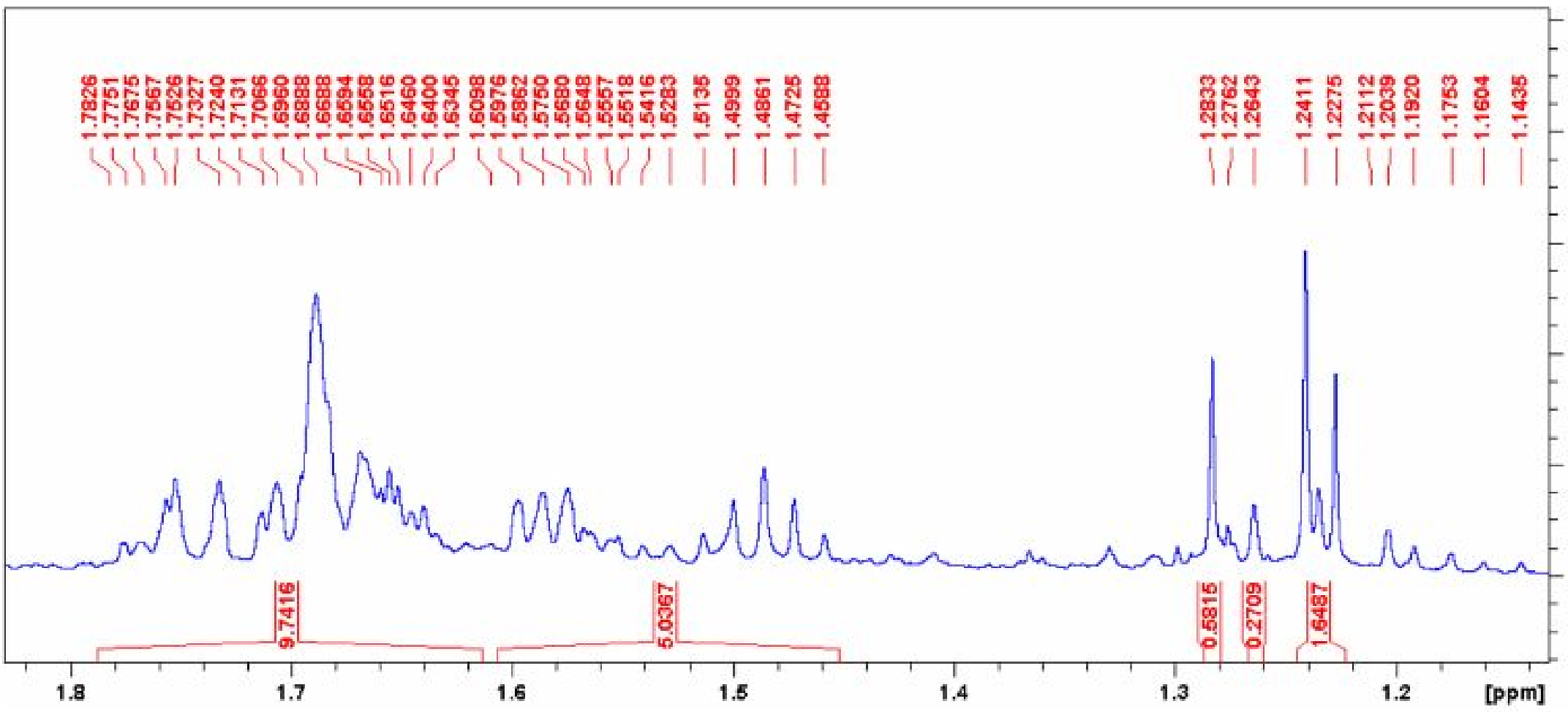
1.7826
1.7751
1.7675
1.7567
1.7526
1.7327
1.7240
1.7131
1.7066
1.6960
1.6888
1.6688
1.6594
1.6558
1.6516
1.6460
1.6400
1.6345
1.6098
1.5976
1.5862
1.5750
1.5680
1.5648
1.5557
1.5518
1.5416
1.5283
1.5135
1.4999
1.4861
1.4725
1.4588
1.2833
1.2762
1.2643
1.2411
1.2275
1.2112
1.2039
1.1920
1.1753
1.1604
1.1435
9.7416
5.0367
0.5815
0.2709
1.6487
[rel]
[ppm]

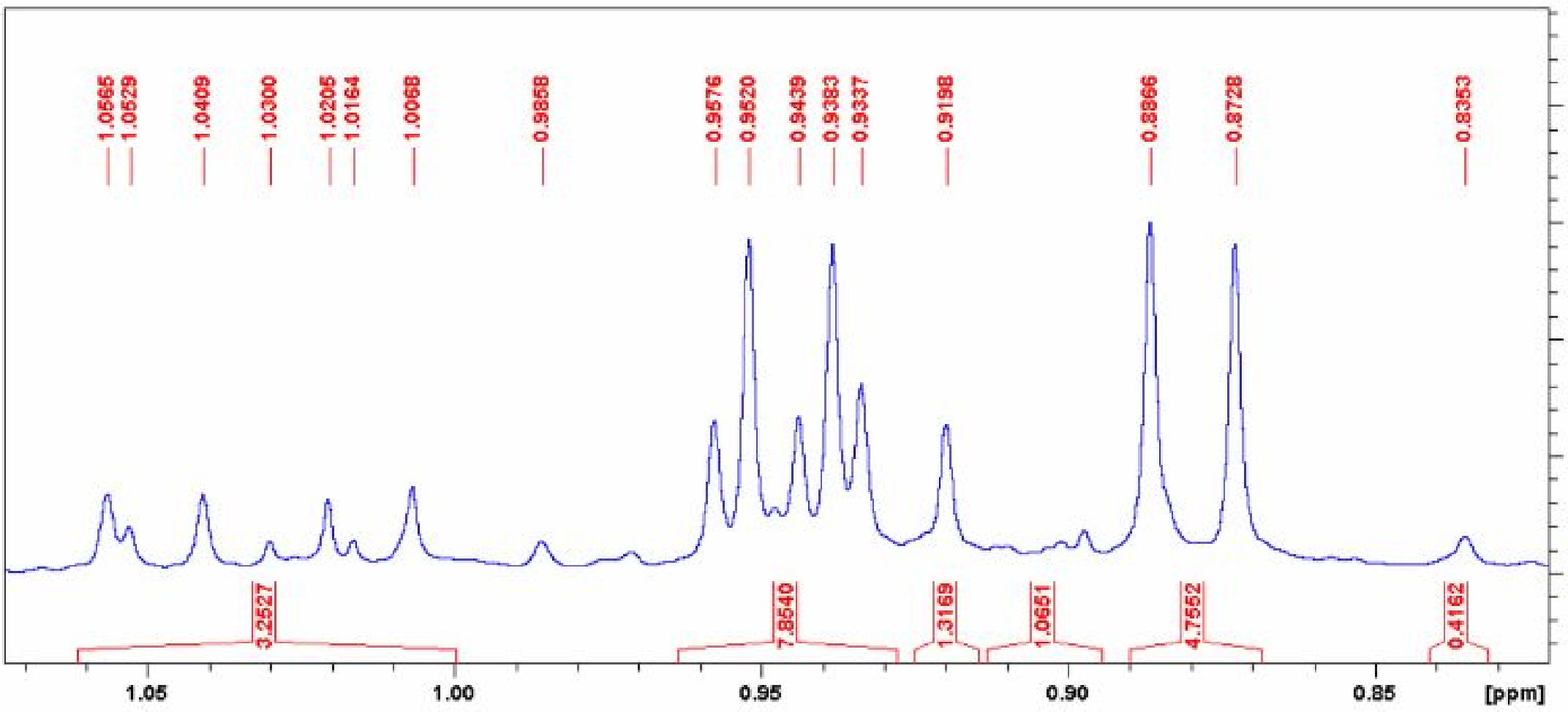
1.0565
1.0529
1.0409
1.0300
1.0205
1.0164
1.0068
0.9858
0.9576
0.9520
0.9439
0.9383
0.9337
0.9198
0.8866
0.8728
0.8353
3.2527
7.8540
1.3169
1.0651
4.7552
0.4162
[rel]
[ppm]

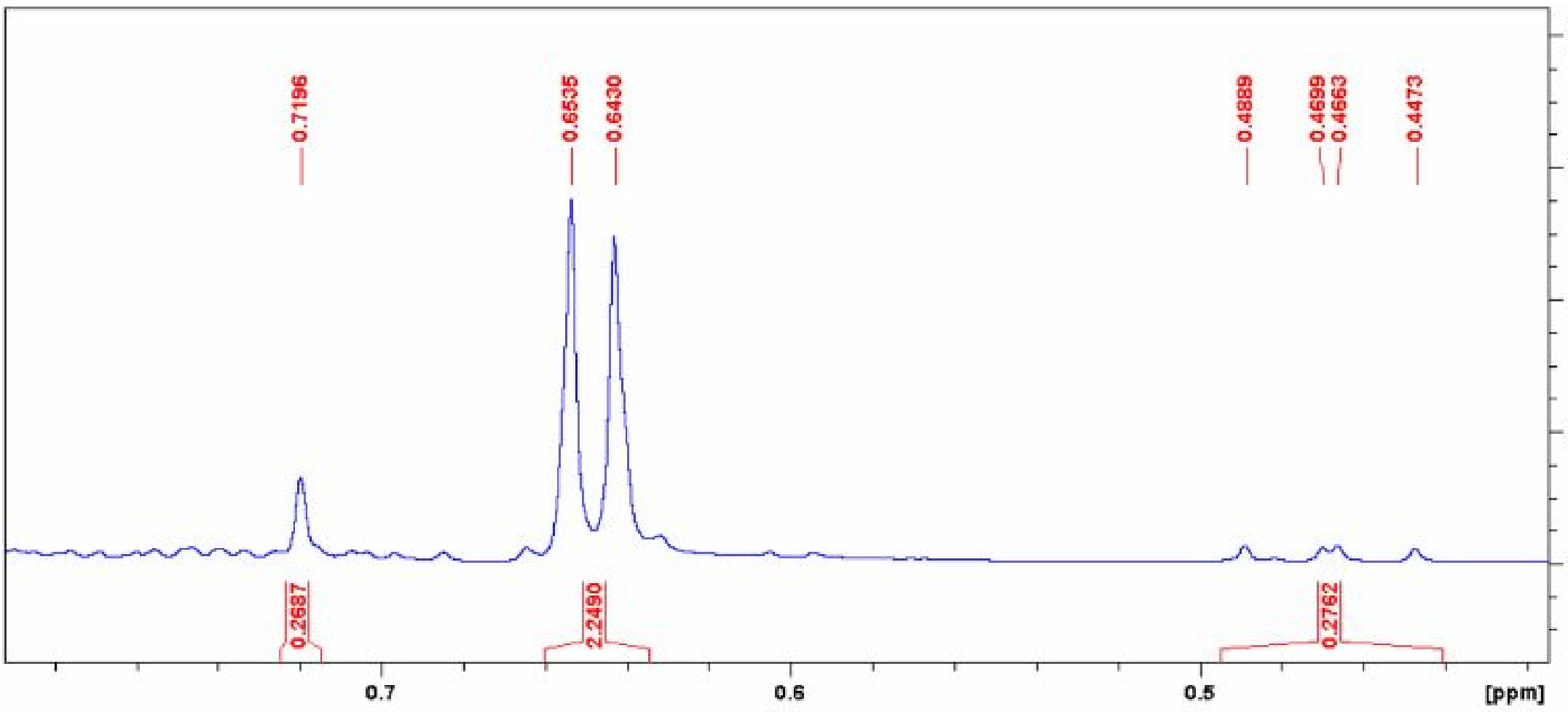
0.7196
0.6535
0.6430
0.4889
0.4699
0.4663
0.4473
0.2687
2.2490
0.2762
[rel]
[ppm]

Figure S41. ^{13}C NMR spectrum of EODs (125 MHz, $CDCl_3$).

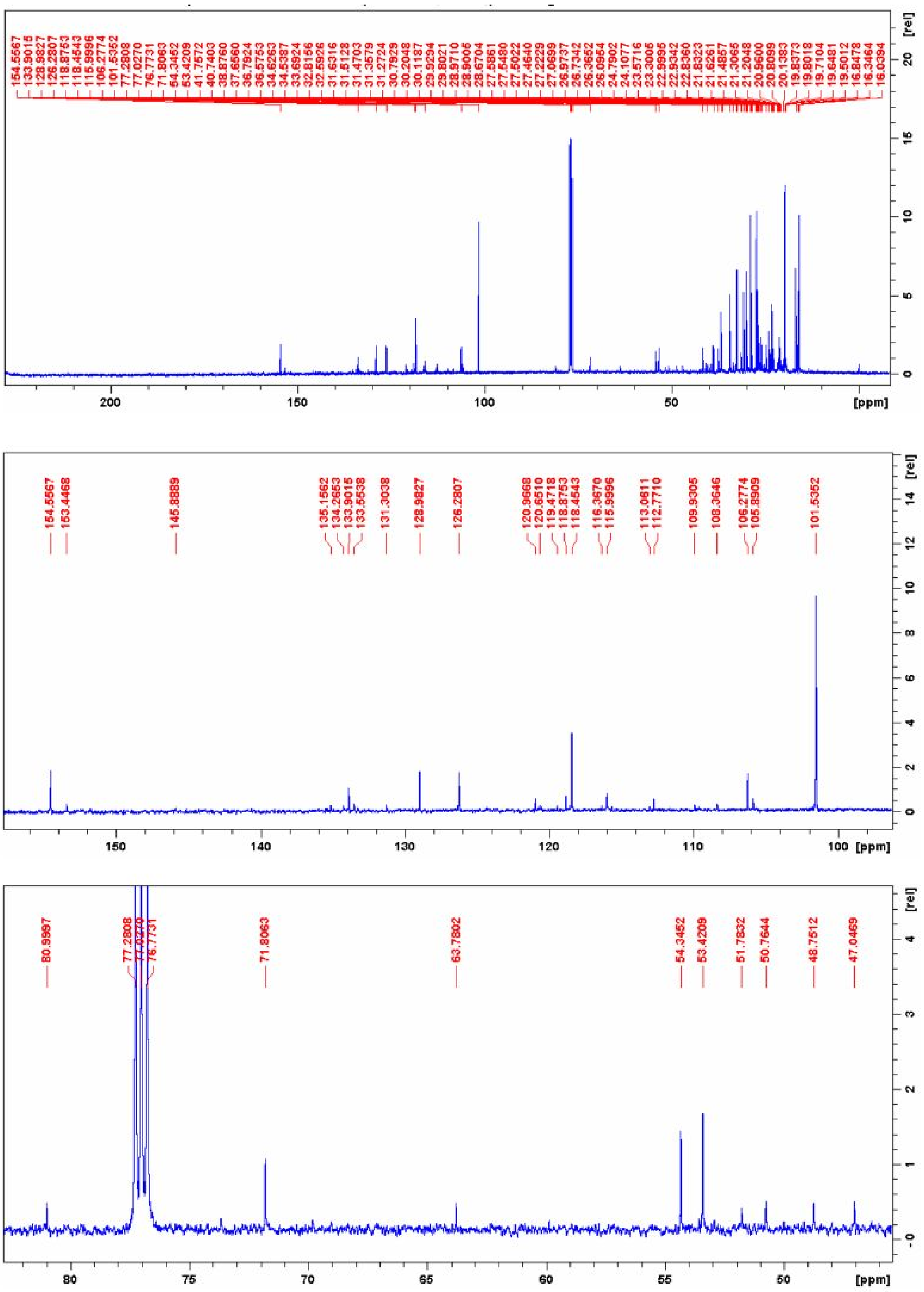

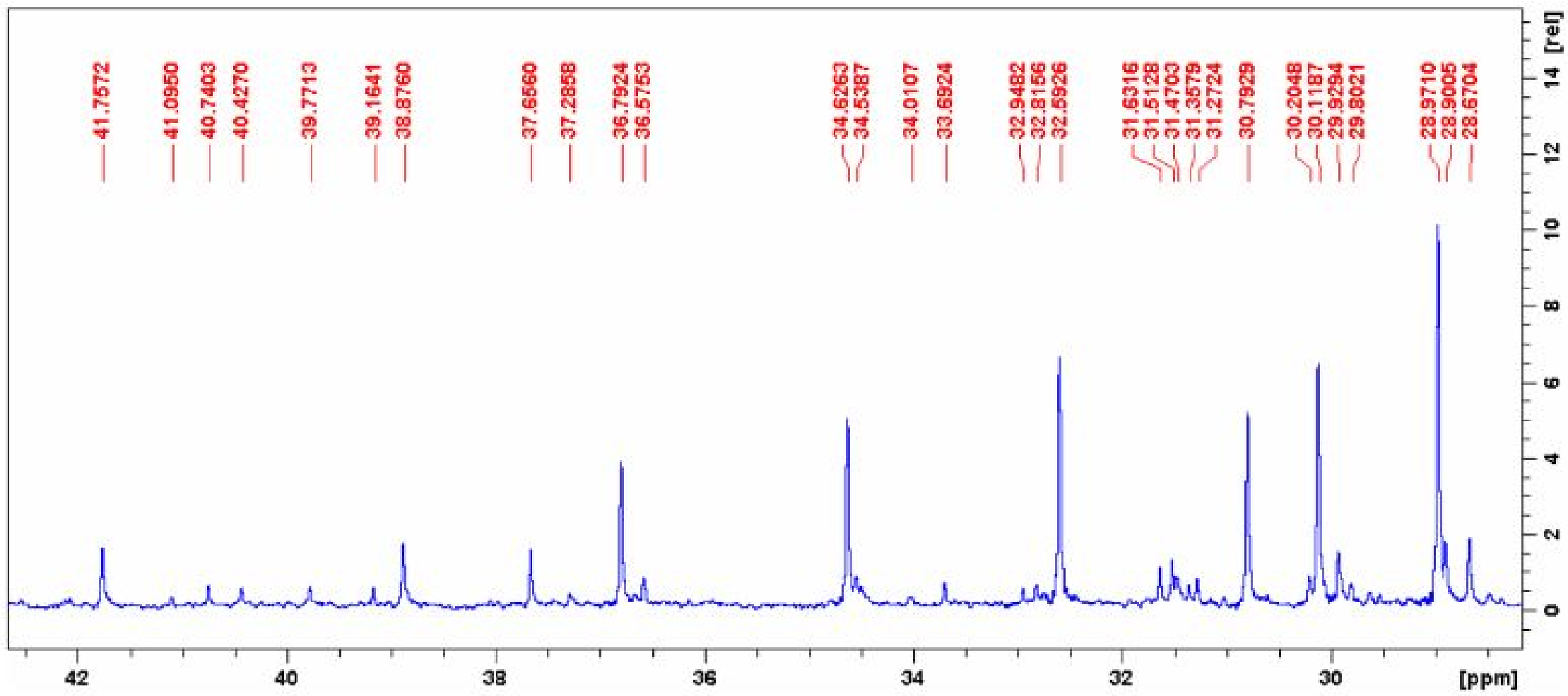

41.7572
41.0950
40.7403
40.4270
39.7713
39.1641
38.8760
37.6560
37.2858
36.7924
36.5753
34.6263
34.5387
34.0107
33.6924
32.9482
32.8156
32.5926
31.6316
31.5128
31.4703
31.3579
31.2724
30.7929
30.2048
30.1187
29.9294
29.8021
28.9710
28.9005
28.6704
[ppm]
[rel]

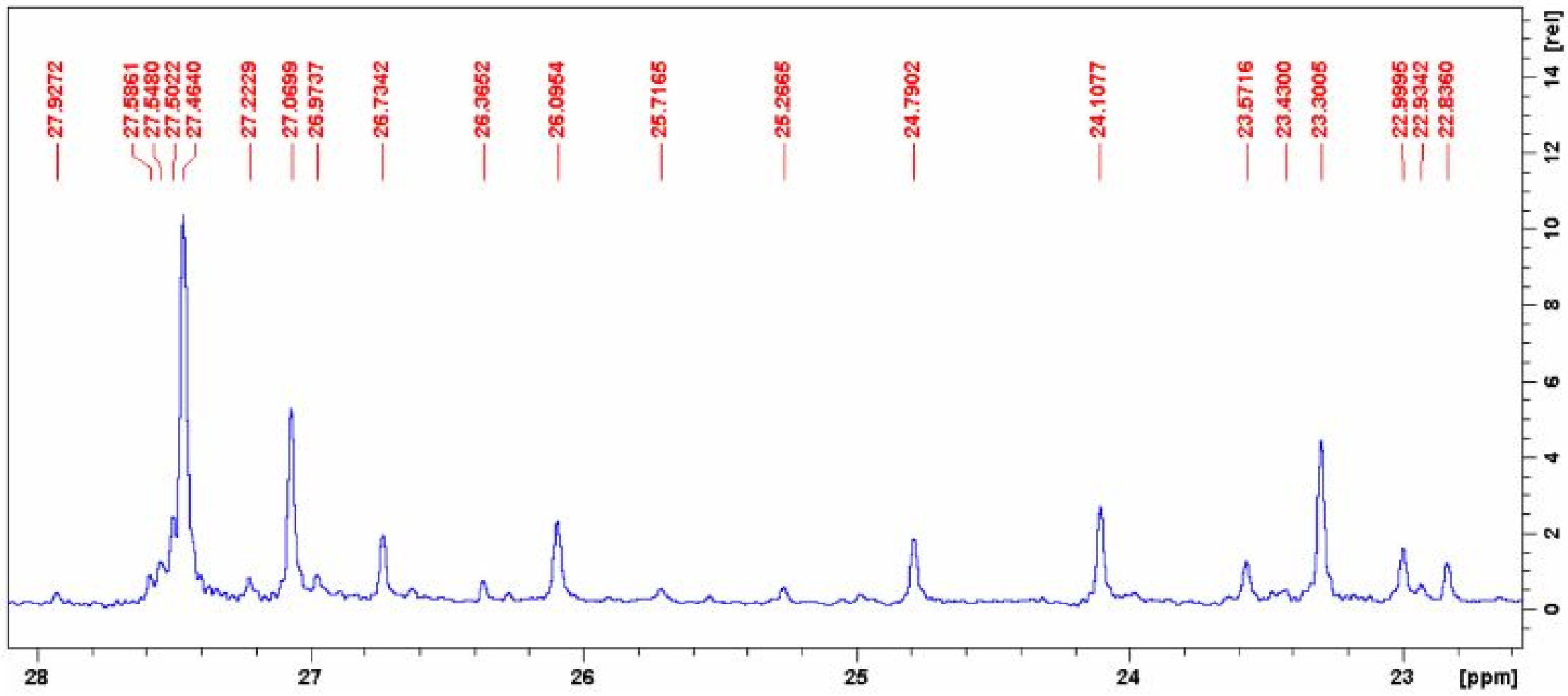

27.9272
27.5861
27.5480
27.5022
27.4640
27.2229
27.0699
26.9737
26.7342
26.3652
26.0954
25.7165
25.2665
24.7902
24.1077
23.5716
23.4300
23.3005
22.9995
22.9342
22.8360
[ppm]
[rel]

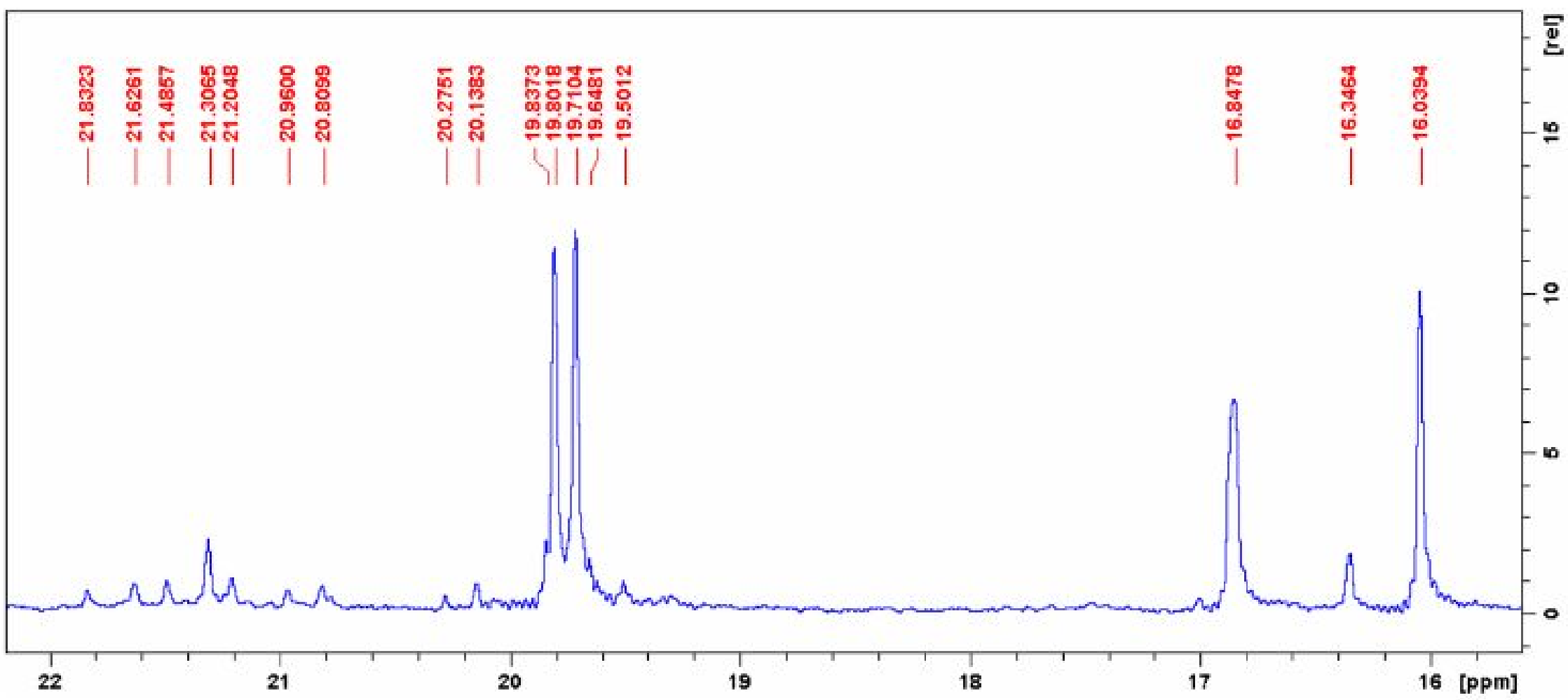

21.8323
21.6261
21.4857
21.3065
21.2048
20.9600
20.8099
20.2751
20.1383
19.8373
19.8018
19.7104
19.6481
19.5012
16.8478
16.3464
16.0394
[ppm]
[rel]

Figure S42. ^{13}C DEPT 135 NMR spectrum of EODs (125 MHz, $CDCl_3$).

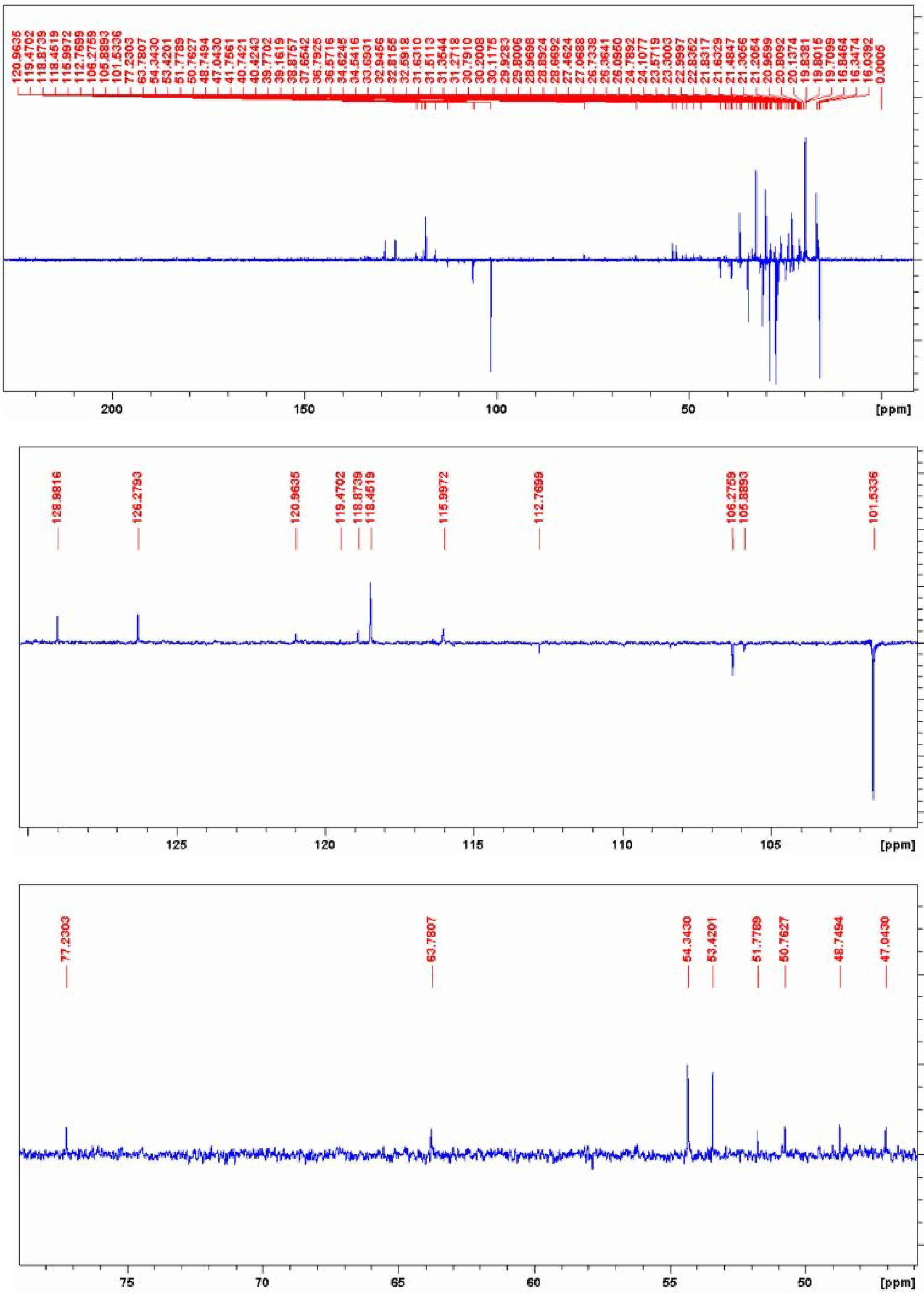

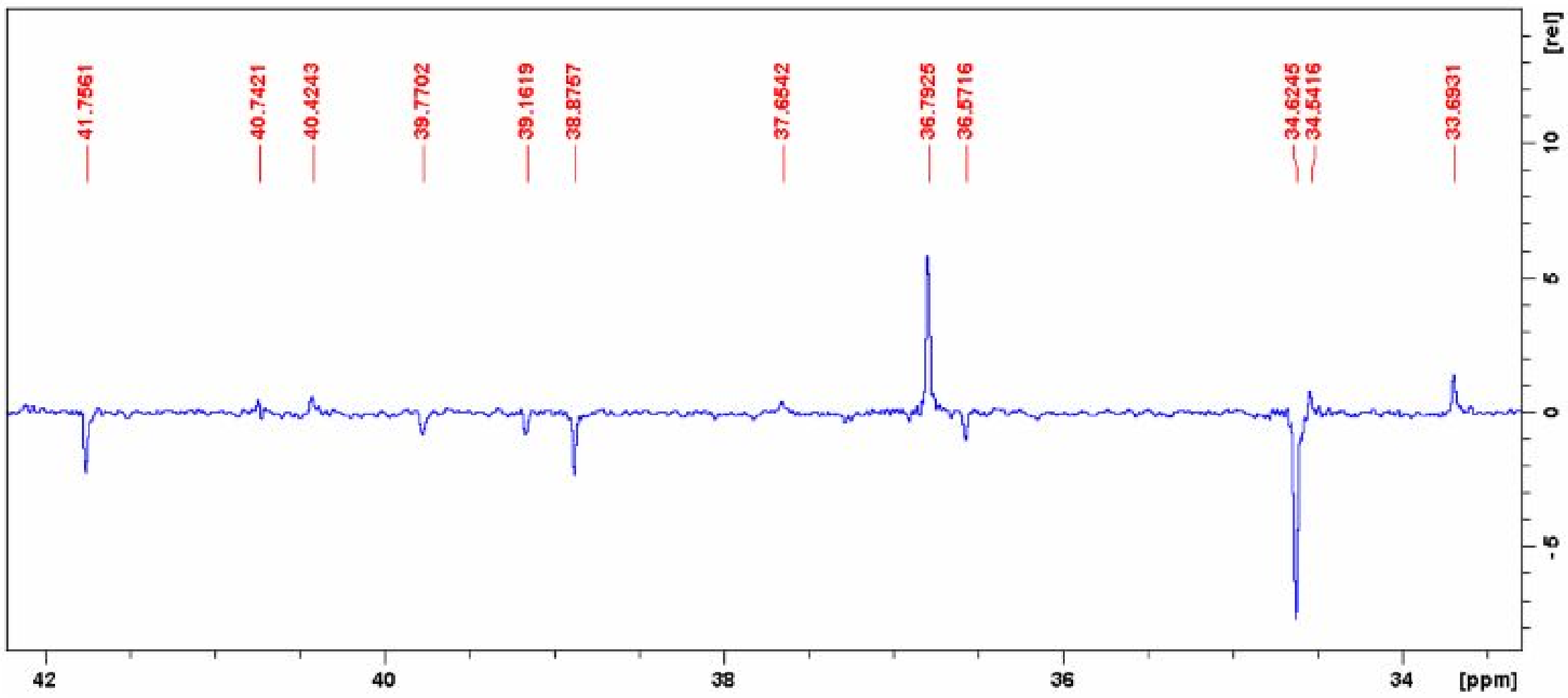
41.7561
40.7421
40.4243
39.7702
39.1619
38.8757
37.6542
36.7925
36.5716
34.6245
34.5416
33.6931
[rel]
[ppm]

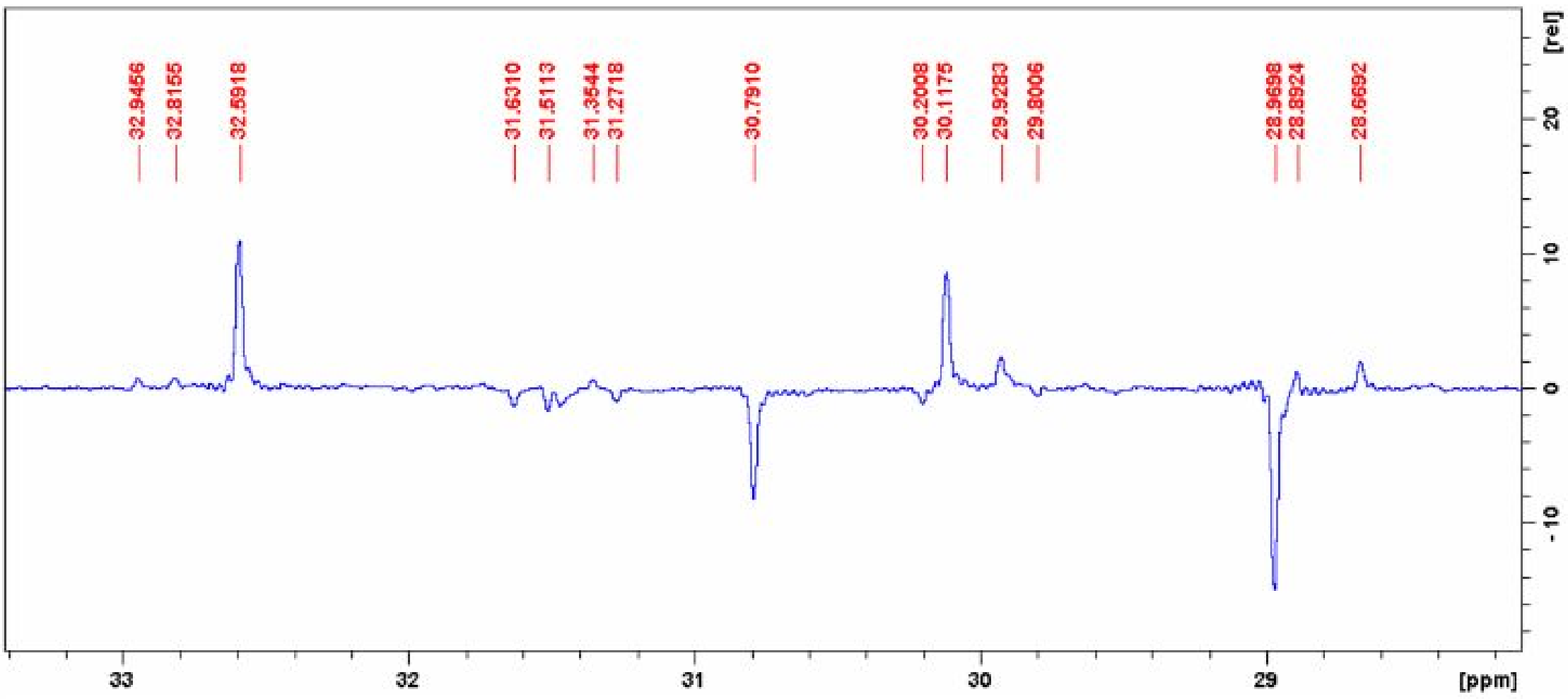
32.9456
32.8155
32.5918
31.6310
31.5113
31.3544
31.2718
30.7910
30.2008
30.1175
29.9283
29.8006
28.9698
28.8924
28.6692
[rel]
[ppm]

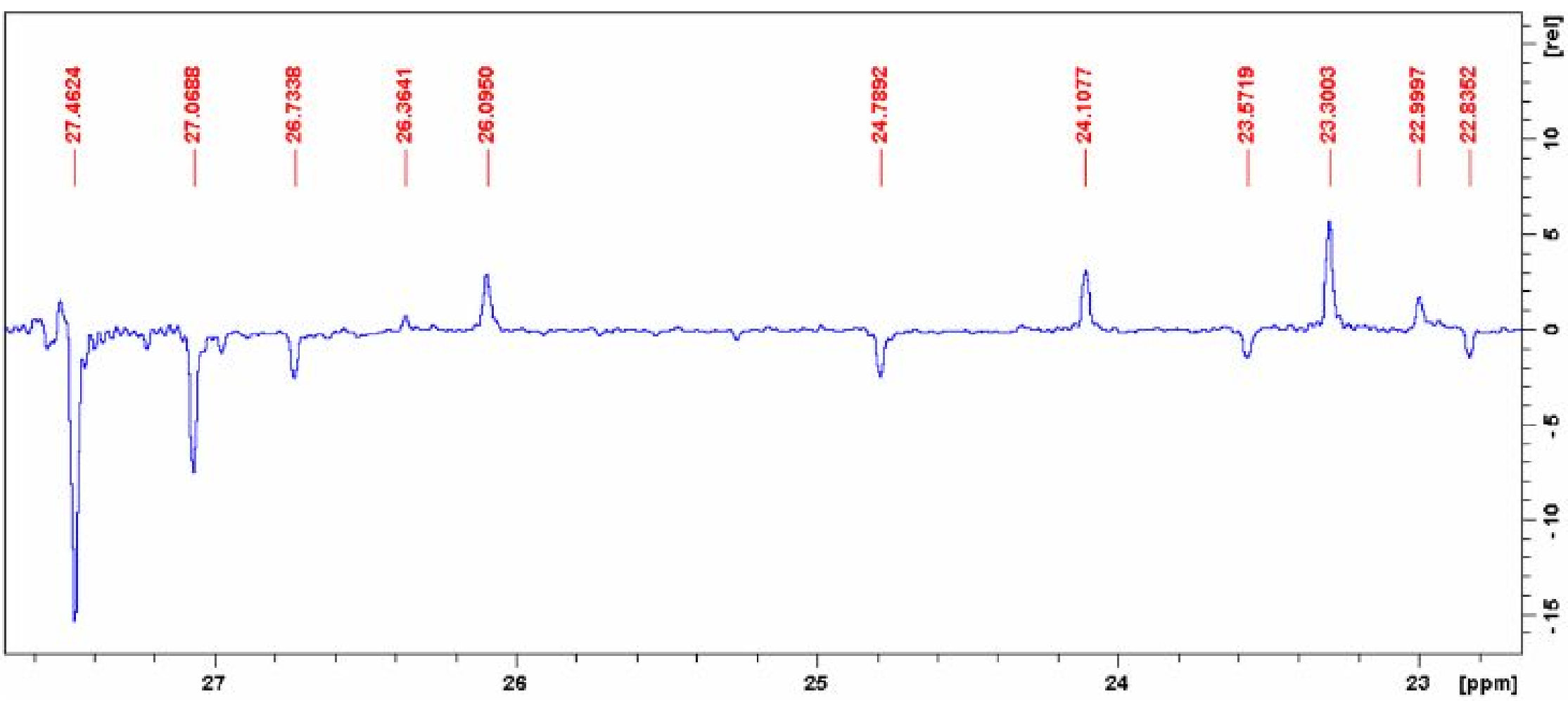
27.4624
27.0688
26.7338
26.3641
26.0950
24.7892
24.1077
23.5719
23.3003
22.9997
22.8352
[rel]
[ppm]

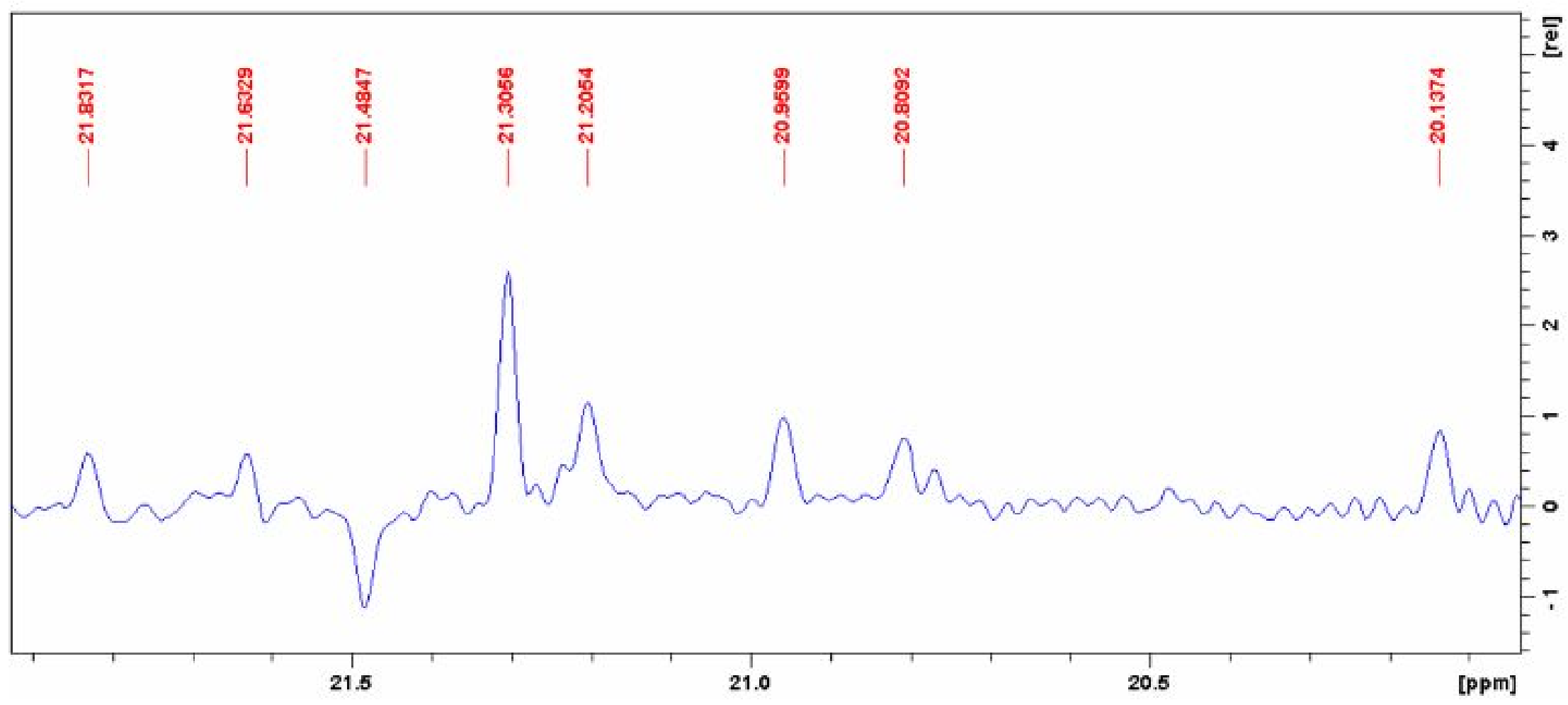
21.8317
21.6329
21.4847
21.3056
21.2054
20.9599
20.8092
20.1374
21.5
21.0
20.5
[ppm]
[rel]

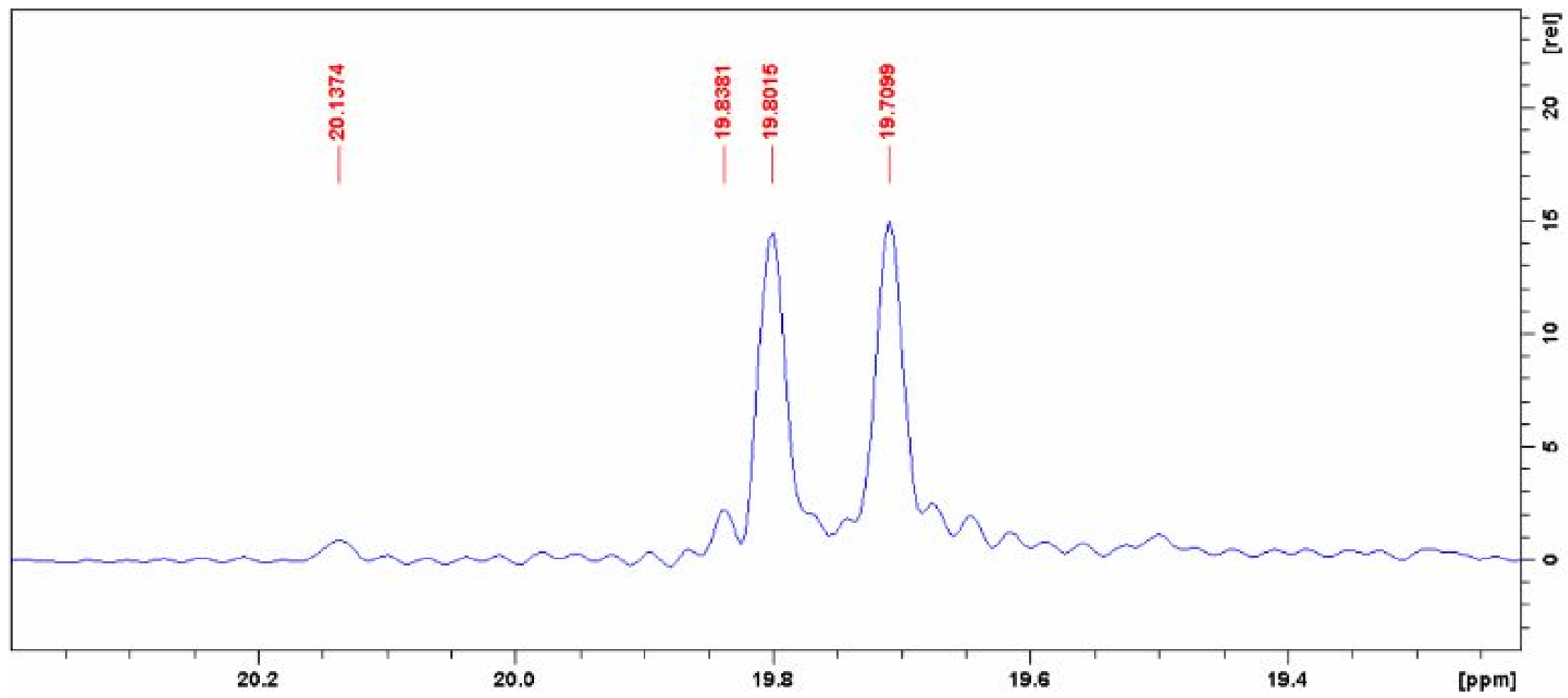
20.1374
19.8381
19.8015
19.7099
20.2
20.0
19.8
19.6
19.4
[ppm]
[rel]

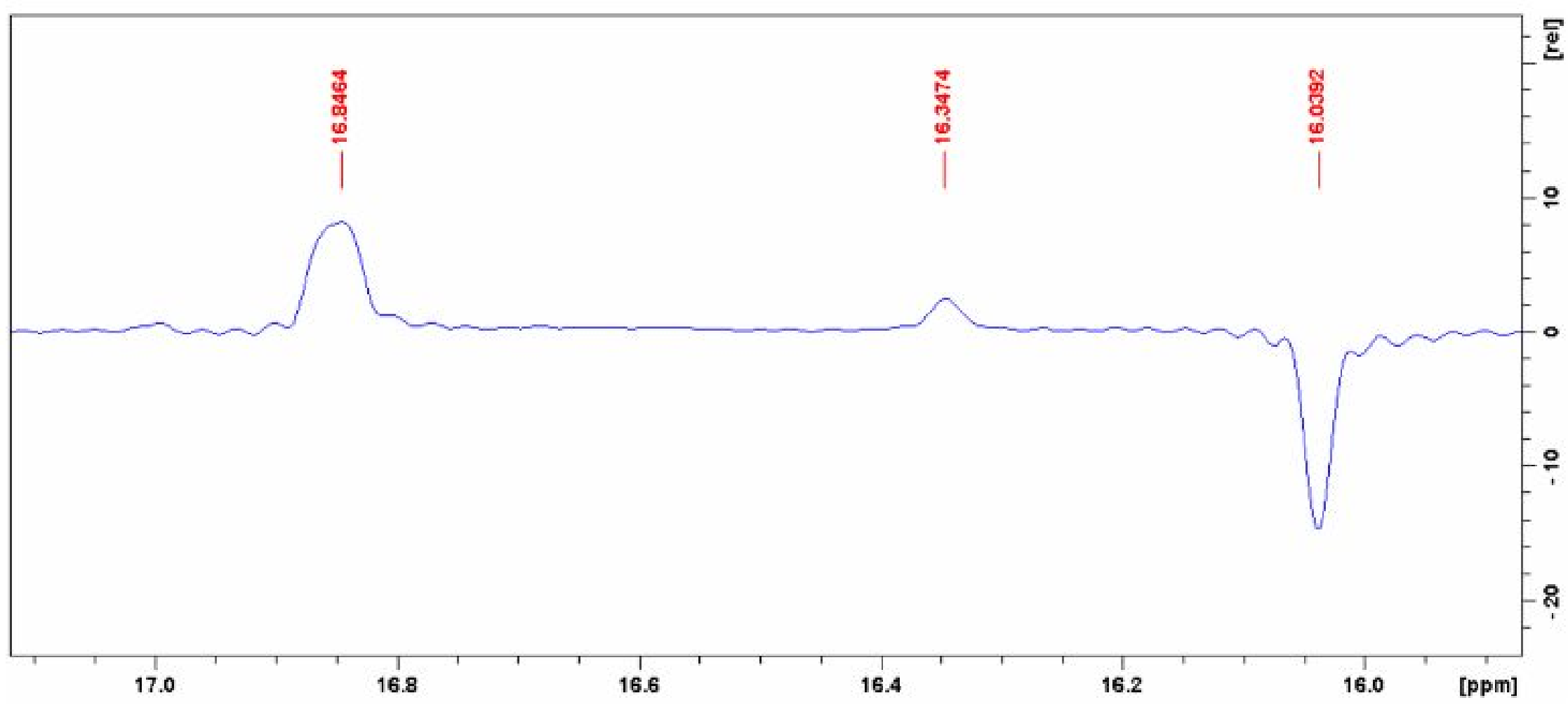
16.8464
16.3474
16.0392
17.0
16.8
16.6
16.4
16.2
16.0
[ppm]
[rel]

Figure S43. FTIR spectra of nanogel formulations. (A) Blank nanogel formulation (nG) composed of Pluronic F127 and Carbopol 974P. (B) EODs-loaded nanogel formulation (nGDs).

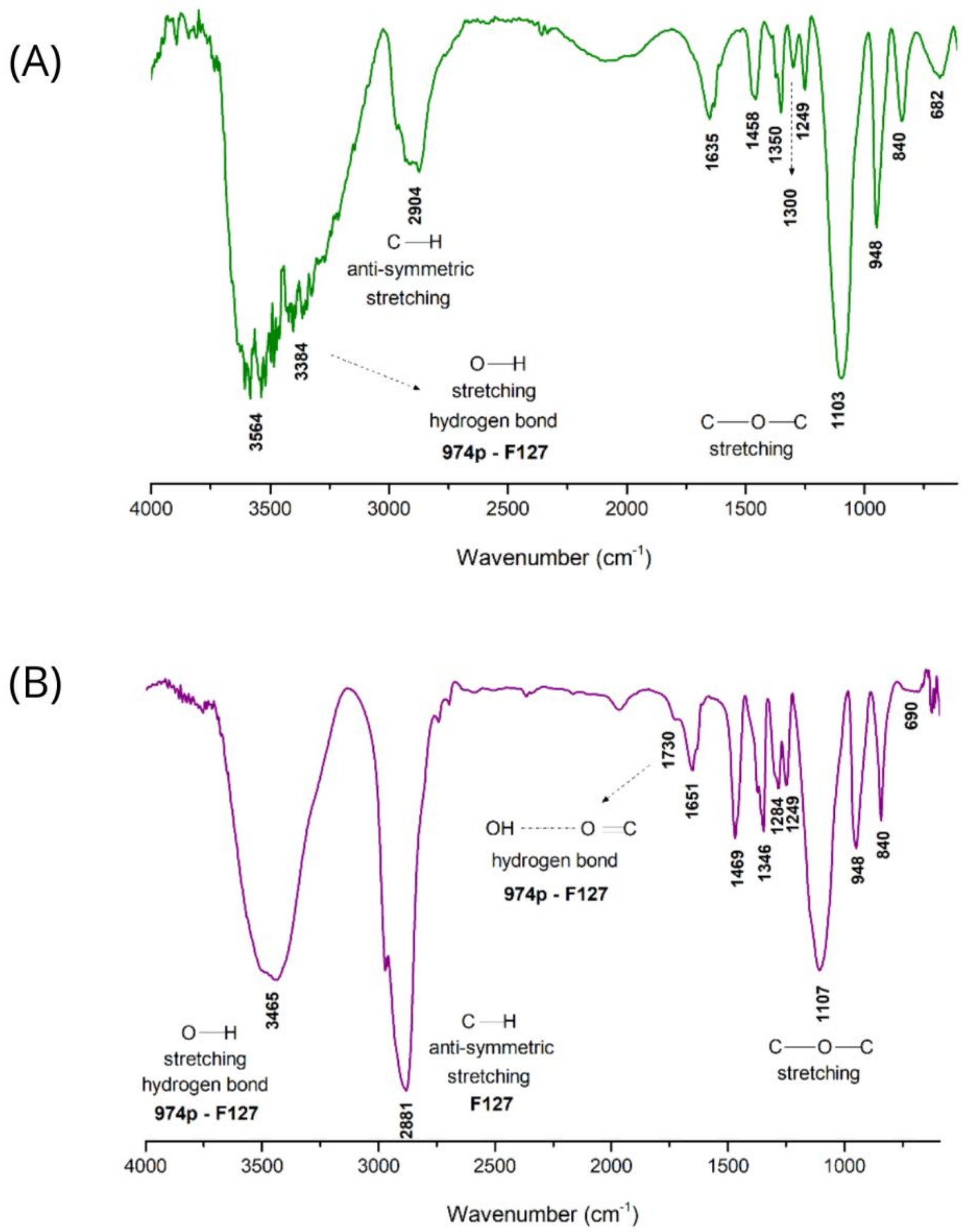

Supplement: Supplementary file 1 [file ao6c04271_si_001.pdf]
